# Loss of PPARγ activity characterizes early protumorigenic stromal reprogramming and dictates the therapeutic window of opportunity

**DOI:** 10.1073/pnas.2303774120

**Published:** 2023-10-10

**Authors:** Joseph A. Caruso, Xianhong Wang, Lyndsay M. Murrow, Carlos Ivan Rodriguez, Chira Chen-Tanyolac, Lisa Vu, Yunn-Yi Chen, Philippe Gascard, Zev J. Gartner, Karla Kerlikowske, Thea D. Tlsty

**Affiliations:** ^a^Department of Pathology, University of California, San Francisco, CA 94143; ^b^Department of Pharmaceutical Chemistry, University of California, San Francisco, CA 94143; ^c^Department of Medicine and Epidemiology and Biostatistics, University of California, San Francisco, CA 94143

**Keywords:** endothelial, ductal carcinoma in situ, invasive breast cancer, pericyte, PPARγ

## Abstract

Although robustly expressed in the disease-free (DF) breast stroma, CD36 is consistently absent from the stroma surrounding invasive breast cancers (IBCs). In this study, we primarily observed CD36 expression in adipocytes and intralobular capillaries within the DF breast. Larger vessels concentrated in interlobular regions lacked CD36 and were instead marked by the expression of CD31. When evaluated in perilesional capillaries surrounding ductal carcinoma in situ, a nonobligate IBC precursor, CD36 loss was more commonly observed in lesions associated with subsequent IBC. Peroxisome proliferator-activated receptor γ (PPARγ) governs the expression of CD36 and genes involved in differentiation, metabolism, angiogenesis, and inflammation. Coincident with CD36 loss, we observed a dramatic suppression of PPARγ and its target genes in capillary endothelial cells (ECs) and pericytes, which typically surround and support the stability of the capillary endothelium. Factors present in conditioned media from malignant cells repressed PPARγ and its target genes not only in cultured ECs and pericytes but also in adipocytes, which require PPARγ for proper differentiation. In addition, we identified a role for PPARγ in opposing the transition of pericytes toward a tumor-supportive myofibroblast phenotype. In mouse xenograft models, early intervention with rosiglitazone, a PPARγ agonist, demonstrated significant antitumor effects; however, following the development of a palpable tumor, the antitumor effects of rosiglitazone were negated by the repression of PPARγ in the mouse stroma. In summary, PPARγ activity in healthy tissues places several stromal cell types in an antitumorigenic state, directly inhibiting EC proliferation, maintaining adipocyte differentiation, and suppressing the transition of pericytes into tumor-supportive myofibroblasts.

## Significance

Stromal CD36 loss during tumor progression reflects impairment of PPARγ expression by tumor-derived factors. This PPARγ loss not only directly liberated EC proliferation but also permitted transition of pericytes towards pro-tumorigenic myofibroblast phenotypes capable of elaborating factors that enhanced both angiogenesis and tumor cell proliferation in vitro and in vivo. In mouse xenograft models, rosiglitazone, an activator of PPARγ, demonstrated anti-tumor effects as an early intervention, but not as a treatment, with tumor formation repressing PPARγ and rendering stromal cells insensitive to ligand. Thus, tumor cell signaling can impair the PPARγ-dependent, anti-tumorigenic state of certain stromal cells found in healthy tissues. This study contributes to the understanding of PPARγ as a stromal target for therapeutic intervention.

The development of a permissive microenvironment is crucial for tumor growth and progression. In a previous study of tissue microenvironments, our laboratory identified CD36 repression as a characteristic of the invasive breast cancer (IBC)-associated stroma and the stroma of tissue at high risk for future cancers (1). Paracrine cues derived from damaged or malignant epithelial cells could reduce CD36 transcription in stromal cells and enhance protumorigenic phenotypes (2). Notably, the mammary glands of cd36 null mice phenocopy the corresponding characteristics in human tissue (1). These CD36-dependent stromal characteristics can appear prior to, or in the absence of, a tumor, suggesting that activating a coordinated stromal program can be an early proactive event in the tumorigenic process rather than only a reactive response to tumor cell signals. The present study seeks to illuminate further the significance of CD36 repression in developing a tumor-supportive stroma.

CD36 is a cell surface scavenger receptor expressed by several stromal cell types, including adipocytes, endothelial cells (ECs), and macrophages. Distinct sites within its extracellular domain allow CD36 to bind a highly diverse set of ligands and participate in a wide range of cellular processes, including the uptake of long-chain fatty acids (3, 4), activation of antiangiogenic signaling in response to thrombospondin-1 (TSP-1) (5, 6), and modification of inflammatory responses when exposed to specific classes of pathogen- or danger-associated molecular patterns present on pathogens, apoptotic cells, or oxidatively modified lipoproteins. CD36 deficiency in the tumor microenvironment may contribute to multiple processes critical to tumor progression, including metabolic reprogramming, inflammation, and angiogenesis (5, 6).

CD36 transcription is regulated by the ligand-activated transcription factor peroxisome proliferator-activated receptor gamma (PPARγ). PPARγ regulates a broad transcriptional program involved in fatty acid metabolism, glucose homeostasis, inflammatory signaling, cellular antioxidant defense, angiogenesis, differentiation, and adipogenesis. Fatty acids and their metabolites are the primary endogenous ligands known to activate PPARγ. In addition, PPARγ can be activated by the thiazolidinedione class of insulin sensitizers, including rosiglitazone. In most cases, PPARγ ligands demonstrate antitumor effects in experimental models (7–9). The beneficial effects of PPARγ ligands in cancer models have been attributed to direct effects on tumor cells (7, 8) and indirect effects on components of the tumor stroma, including a reduction in angiogenesis (9, 10). Furthermore, PPARγ activation is known to suppress fibrosis and proinflammatory signaling (11, 12) and preserve adipocyte differentiation (13).

In this study, we primarily observed CD36 immunopositivity in specialized capillaries surrounding the disease-free (DF) breast epithelium and in adipocytes. CD36 loss and CD31 gain in the capillary vasculature were more commonly observed surrounding ductal carcinoma in situ (DCIS) associated with subsequent IBC. Evidence for the suppression of CD36 and other components of the PPARγ transcriptional program was observed during the progression of multiple human cancers, including breast, colon, and lung cancers. Tumor-derived growth factors and cytokines suppressed PPARγ in adipocytes, capillary ECs, and pericytes. We also demonstrated that PPARγ suppressed the transition of pericytes toward a myofibroblast-like phenotype capable of supporting tumor cell growth and angiogenesis in vitro and in vivo. A potent PPARγ activator, rosiglitazone, demonstrated antitumor activities when administered early in the progression of mouse mammary xenograft tumors but did not exhibit these activities when treatment was begun following the formation of a palpable tumor. As a cancer-preventative strategy (14) (i.e., prior to the loss of PPARγ expression), the administration of the PPARγ agonists may inhibit the development of a tumor-supportive stroma by targeting angiogenesis, pericyte-to-myofibroblast transition, and adipocyte dedifferentiation.

## Results

### CD36 Immunopositivity Distinguishes Intralobular Capillaries From Other Components of the Breast Microvasculature.

A previous report indicates that CD36 expression distinguishes healthy from tumor-associated breast stroma (1). We first examined bulk RNA-sequencing data from The Cancer Genome Atlas (TCGA) to validate and extend this observation. A reduction in CD36 transcript levels was evident in a wide range of tumor types compared to DF tissues (*SI Appendix*, Fig. S1). We confirmed a significantly lower level of stromal CD36 expression by immunohistochemical (IHC) analysis of breast, lung, colon, and esophageal carcinomas compared to DF tissues (*SI Appendix*, Fig. S2 *A*–*D*).

An initial goal of this study was to identify the cell types accounting for reduced CD36 immunopositivity in the IBC-associated stroma compared to the DF breast stroma (1). Analysis of our recently published single-cell RNA-sequencing (scRNA-seq) data from DF breast (15) and complementary data from histologically normal breast tissue adjacent to IBC (16) demonstrated that the transcript levels of CD36 were highest in ECs, perivascular cells, and macrophages (*SI Appendix*, Fig. S3). Notably, CD36 is part of a broader transcriptional program regulated by PPARγ indicated by the coincident expression of several other PPARγ target genes with CD36. Importantly, CD36^+^ adipocytes (*SI Appendix*, Fig. S4) were absent from these scRNA-seq studies due to their large size and fragility, which resulted in their loss during cell isolation.

**Fig. 1.**
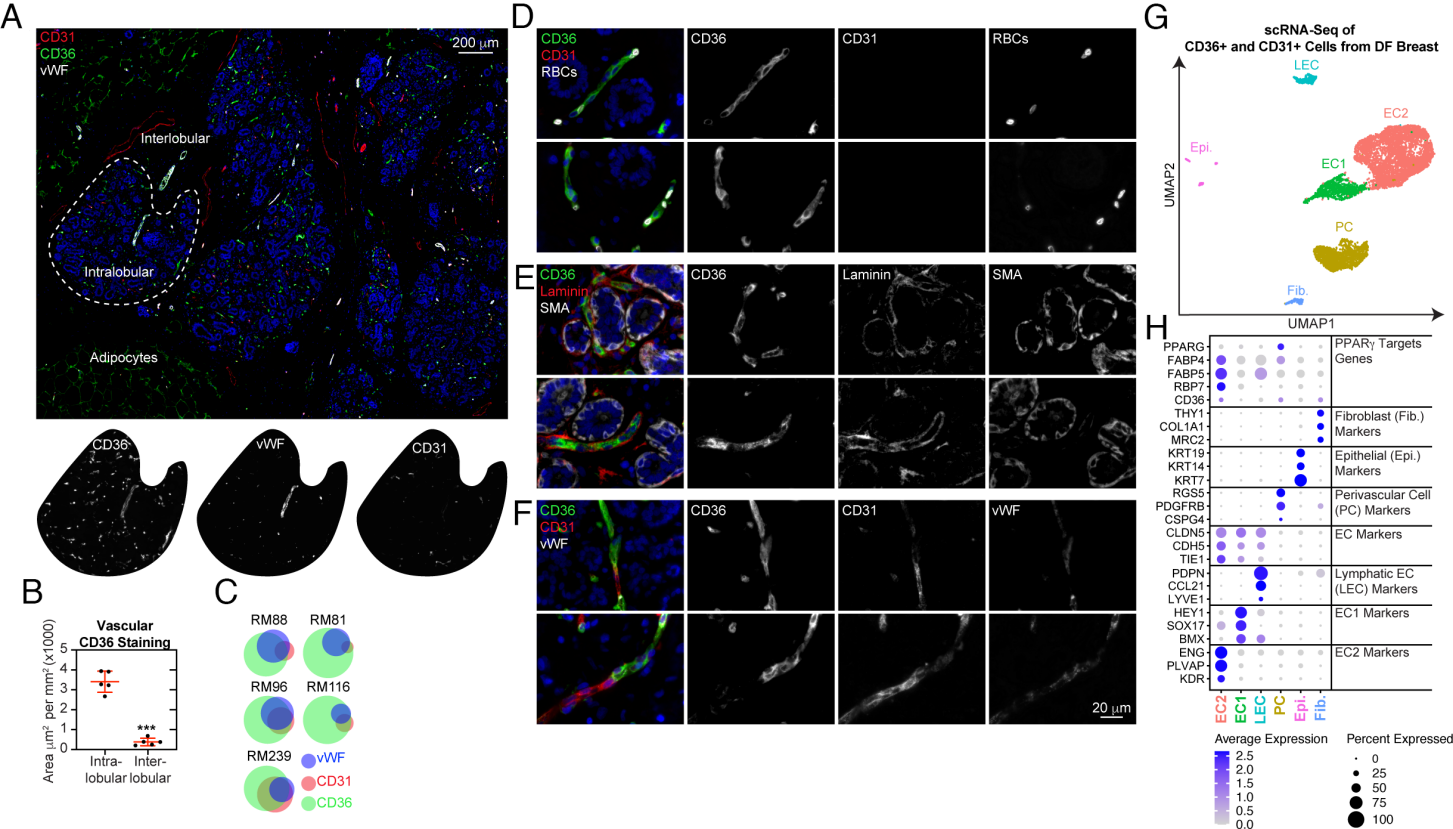
CD36 identifies intralobular capillaries lacking CD31 and vWF immunopositivity in the DF breast. (*A*) DF breast tissue from five donors aged 16–53 were subjected to mIHC analysis of CD36, CD31, and vWF. (*B*) The CD36-positive staining area, excluding adipocytes, was determined and normalized to the total area measured for the intralobular and interlobular stroma. (*C*) Following cell segmentation, vascular cells were scored and binned based on staining for each marker (CD36^+^, CD31^+^, and/or vWF^+^). Area-proportional Euler diagrams demonstrate the relationships between markers (overlaps between circles) and the proportion of each marker expressed within the intralobular vasculature (area of the circle). (*D–F*) DF breast was subjected to mIHC analysis for the additional markers validating vascular identity. (*G*) CD31^+^ and/or CD36^+^ from the DF breast (*n* = 14; median age: 24; range: 19–36) were subjected to MULTI-seq analysis. UMAP projection of cell clusters is shown. (*H*) A dot plot of selected marker genes indicative of cell cluster identity is shown. The size of the dot indicates the percentage of cells expressing the gene in each cluster, and the color of the dot represents the average expression level.

Given the abundance of CD36 transcripts observed in vascular cell types, we performed multiplex immunohistochemistry (mIHC) analysis of CD36 in combination with von Willebrand Factor (vWF) and CD31 (Fig. 1*A* and *SI Appendix*, Fig. S5), commonly used markers for the visualization and quantification of microvasculature in histological sections (17). Excluding CD36^+^ adipocytes, distinguished by their large size and octagonal shape, the remaining CD36 immunopositivity was concentrated (ninefold greater CD36^+^ staining area) in the intralobular stroma (directly surrounding the epithelial components of each lobule) compared to the interlobular stroma (the area between lobules) (Fig. 1*B*). Surprisingly, only one-third of CD36 immunoreactive structures within the intralobular stroma expressed CD31 and/or vWF (Fig. 1*C*). Despite the lack of CD31 and vWF, CD36-expressing structures were validated as vasculature since they were a) tightly juxtaposed to the acinar epithelial structures of the breast, b) contained red blood cells (RBCs) within their lumens in the single file pattern expected within narrow capillary vessels (Fig. 1*D*), c) were enveloped by basement membrane-associated laminins (Fig. 1*E*) and d) were contiguous with CD31-expressing vessels (Fig. 1*F*).

CD36^+^ and/or CD31^+^ cells from 14 donors were isolated by fluorescence-activated cell sorting (FACS) and then subjected to scRNA-sequencing to gain further insights into the vascular cell types in the DF breast. Six cell clusters (Fig. 1*G*) were identified based on differentially expressed markers (16) (Fig. 1*H*), including small subpopulations of fibroblasts, epithelial cells, and lymphatic ECs. Consistent with our mIHC analysis, we identified two distinct populations of vascular ECs. EC2 was enriched in genes associated with capillary identities (CD105/ENG, PLVAP, and VEGFR2/KDR). CD36 and several additional PPAR*γ* target genes, including fatty acid binding proteins 4 (FABP4) and 5 (FABP5) and retinol-binding protein 7 (RBP7) (18), were solely expressed in capillary ECs (EC2). In addition, we identified a population that expressed markers of pericytes (PDGFRB, RGS5, and CSPG4) (19) as well as CD36 and several other PPARγ target genes. Additional differentially expressed genes are listed in supplemental Datasets S1–S4. Notably, we rarely observed CD36 immunopositivity in CD68^+^ macrophages and never in CD4^+^ T cells (*SI Appendix*, Fig. S6), PDGFRβ^+^ fibroblasts (*SI Appendix*, Fig. S7), nor cytokeratin 7^+^ epithelial cells (*SI Appendix*, Fig. S8) when evaluated in both DF breast and IBC specimens by mIHC.

Thus, extending our previous study (1), we determined that adipocytes, intralobular capillary ECs, and pericytes were the stromal components accounting for most CD36 immunopositivity in the DF breast. Through scRNA-seq, we identified several additional PPARγ-regulated genes correlated with CD36 expression in vascular cell types.

### Confocal Microscopy Visualizes the Architecture of the CD36-Expressing Intralobular Capillary Network.

**Fig. 2.**
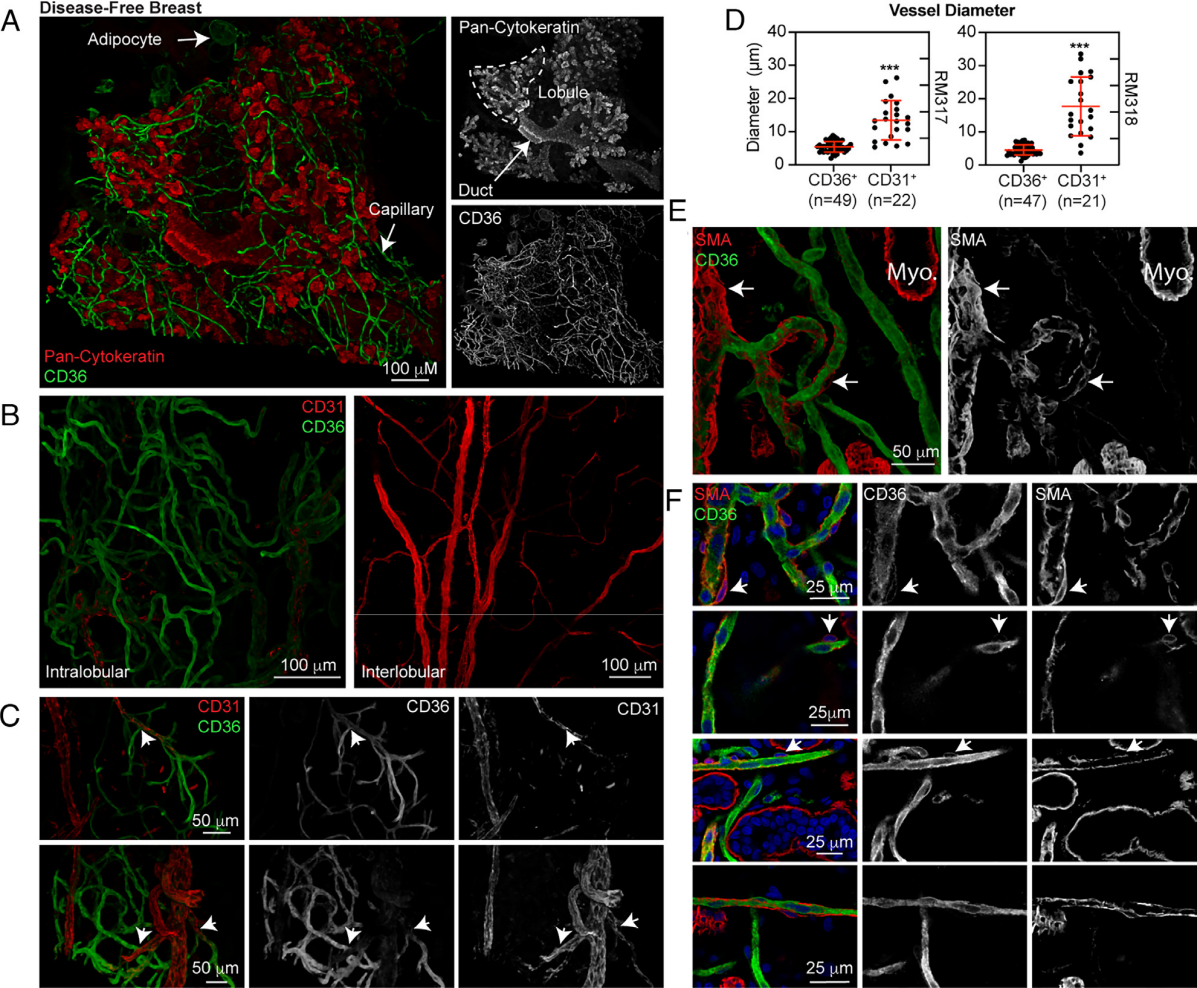
Confocal imaging of CD36 immunopositivity in intact human breast tissue reveals the architecture of the intralobular capillary network. (*A*) Tissue from a 29-y-old nulliparous woman (RM320) was immunostained for CD36 and pan-cytokeratin (250 μm Z-stack). (*B*) Tissue from the same donor was immunostained for CD36 and CD31. DAPI staining (not shown) was utilized to select an epithelial-rich area (*Left*) and an area within the interlobular stroma devoid of epithelium (*Right*) (200 μm Z-stacks). (*C*) Tissue sections from a 21-y-old nulliparous woman (RM317; *Top* row) and a 39-y-old parous woman (RM318; *Bottom* row) were immunostained for CD36 and CD31 (50−100 μm Z-stacks). The white arrows indicate branch points between larger vessels and intralobular capillaries. (*D*) Vessel diameter was measured (outer edge to outer edge). (*E*) The same tissues were immunostained for CD36 and SMA, a marker of myoepithelial cells (Myo.) and perivascular cells (arrows). (*F*) Tissue sections from RM317 and RM318 were immunostained for CD36 and SMA (50−100 μm Z-stacks). The white arrows indicate several pericytes expressing CD36.

Identifying CD36 as a specific marker of intralobular capillary ECs and surrounding pericytes presented an opportunity to visualize the three-dimensional architecture of the breast microvasculature using optical clearing and confocal microscopy. CD36^+^ capillary vessels wrapped tightly around the epithelial components (pan-cytokeratin^+^) of terminal ductal lobular units (TDLUs). CD36 expression was also apparent in adipocytes (Fig. 2*A*). Notably, CD36 expression was primarily observed in intralobular capillaries surrounding the DF breast epithelium (Fig. 2*B*). Higher magnification imaging identified branch points in and around TDLUs between CD36^+^CD31^−^ capillaries and CD36^−^CD31^+^ vessels (Fig. 2*C*). In contrast to CD31, VE-cadherin could be visualized by confocal imaging in both CD36^+^ and CD36^−^ vessels (*SI Appendix*, Fig. S9). The diameters of CD36^−^CD31^+^ vessels were confirmed to be significantly larger compared to CD36^−^CD31^−^ capillaries (Fig. 2*D*). Notably, we observed CD36 not only in ECs but also in surrounding pericytes. Immunostaining with α-smooth muscle actin (SMA) highlighted myoepithelial cells surrounding breast acini, vascular smooth muscle cells surrounding arterioles/venules, and pericytes stabilizing the capillary network (Fig. 2*E*). Although difficult to visualize due to their small cell bodies and the extension of thin processes along the capillary endothelium, pericytes expressing SMA and CD36 could be observed in the DF breast by confocal microscopy (Fig. 2*F*).

Our analysis of CD36 by confocal microscopy highlighted the full extent of the capillary network in DF breast tissue, which lacked expression of the conventional vascular marker CD31. In addition, we visualized CD36-expressing pericytes supporting the capillary network.

### Lack of CD36 in the Capillary Bed Surrounding DCIS Lesions Distinguishes Lesions Associated with Subsequent IBC.

We previously reported CD36 loss in the stroma of mammographically dense breast tissue, which is associated with a higher risk of IBC development, suggesting that CD36 loss can precede the development of a detectable invasive tumor (1). This led us to hypothesize that loss of CD36 expression in the vasculature surrounding noninvasive DCIS lesions would be associated with subsequent IBC. To analyze clinically annotated FFPE tissue sections, we developed an mIHC assay for FFPE sections to consistently quantify CD36 and CD31 immunostaining in the vasculature. This assay used vimentin as a tertiary marker to exclude immune cells (mostly CD31^+^ plasma cells). Adipocytes, primarily observed in the DF breast but absent from the invasive tumor area (*SI Appendix*, Fig. S4), were manually removed from the analysis based on their recognizable morphology. In the remaining vimentin-expressing structures, the area staining positive for CD36, CD31, or both CD31 and CD36 was determined using fluorescent intensity thresholds capturing specific staining versus primary antibody omitted controls (*SI Appendix*, Fig. S10).

**Fig. 3.**
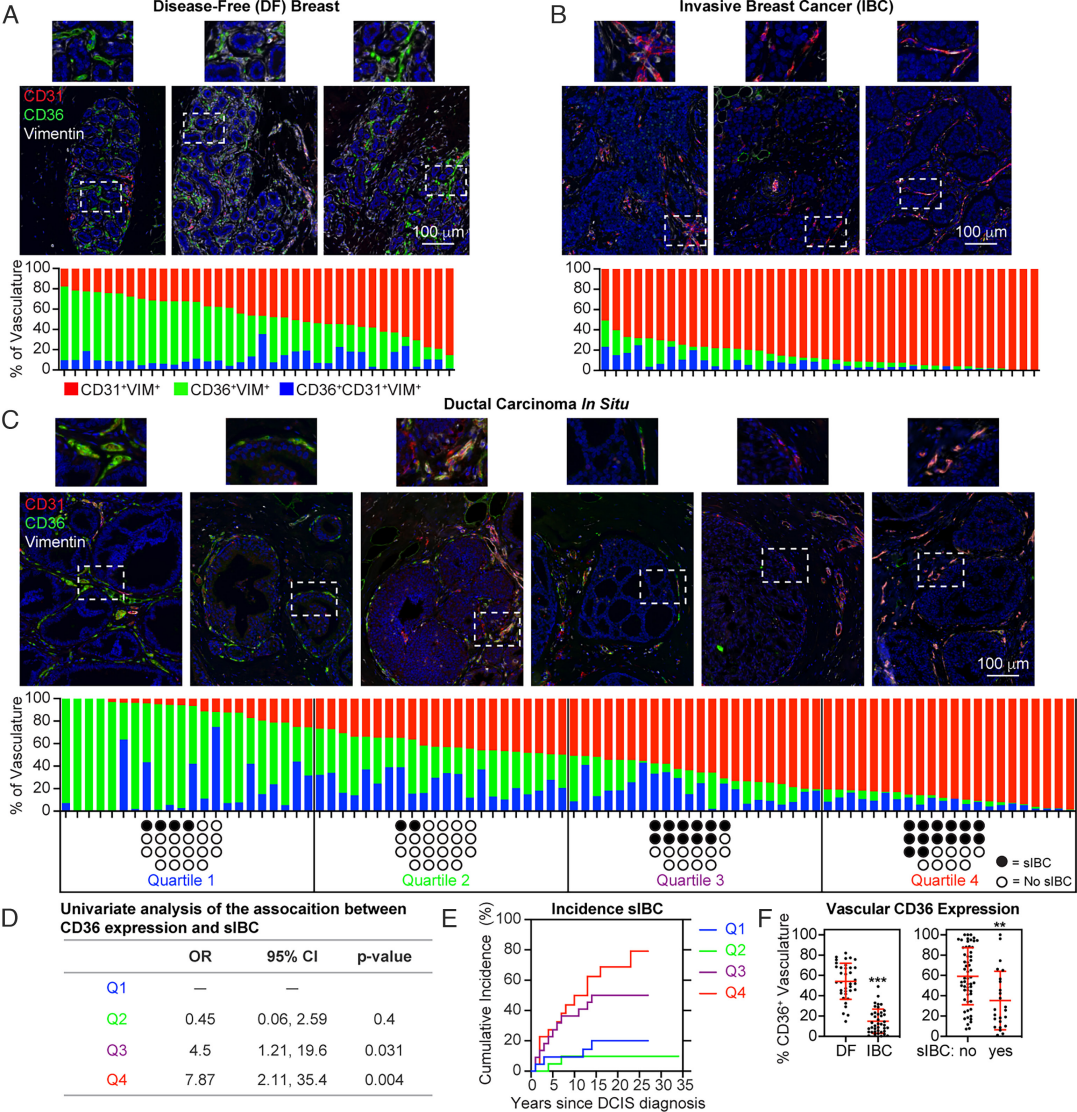
Loss of CD36 immunopositivity in the vasculature during breast cancer progression. (*A*) DF breast specimens (*n* = 36; median age: 30; range 16–78) were subjected to mIHC for CD36, CD31, and vimentin. The proportion of the total vasculature measured in the imaging area expressing CD36^+^ (green), CD31^+^ (red), or CD31^+^CD36^+^ (blue) is graphed. (*B*) 40 IBC specimens (median age: 52; range 30–79) were subjected to mIHC. (*C*) DCIS specimens (*n* = 88; median age: 57; range: 22–86) were subjected to mIHC. The patient population was split into quartiles based on the proportion of vasculature expressing CD36. Black circles indicate the development of subsequent IBC (sIBC) (median follow-up cases: 5 y, range: 1–16; controls: 19 y, range: 1–34). (*D*) Logistic regression was used to estimate associations between the proportion of breast vasculature expressing CD36 and sIBC. The odds ratio (OR) and 95% CI were calculated. (*E*) The cumulative incidence of sIBC following DCIS diagnosis is graphed for each quartile. (*F*) The total percentage of CD36-expressing vascular phenotypes (CD36^+^ and CD31^+^CD36^+^) in DF and IBC specimens, as well as DCIS cases (sIBC) and controls (no sIBC), is graphed.

DF breast tissues from 36 donors were used to establish a baseline for the expression of CD36 and CD31 in the vasculature (Fig. 3*A*). CD36 and CD31 were nearly mutually exclusive in DF breast tissue when evaluated by mIHC. Neither donor age, race, or parity were significantly correlated with the relative proportion of the vasculature expressing CD36 or CD31. Surgical specimens from 40 women diagnosed with IBC, without neoadjuvant chemotherapy or radiotherapy, were also analyzed for vascular CD36 and CD31 immunopositivity (Fig. 3*B*). Vascular CD36 expression was consistently absent within the tumor area relative to vascular CD31 expression in specimens from multiple IBC subtypes (ER^+^PR^+/−^HER2^−^ [*n* = 18], ER^+^PR^+/^^−^ HER2^+^ [*n* = 3], ER^−^PR^−^HER2^+^ [*n* = 4], ER^−^PR^−^HER2^−^ [*n* = 7], and unknown [*n* = 8]). Finally, we examined the expression of CD36 and CD31 in the vasculature surrounding noninvasive DCIS (n=88), collected following lumpectomy from 88 patients. The vasculature surrounding DCIS lesions existed in a continuum from predominately CD36^−^ expressing, resembling DF breast vasculature, to predominately CD31-expressing, resembling IBC vasculature (Fig. 3*C*).

We next examined the association between CD36 immunopositivity in the vasculature surrounding DCIS and the subsequent development of ipsilateral IBC (20). Patients were separated into four quartiles (Q1, Q2, Q3, and Q4) based on their relative expression of CD36 and CD31. The first two quartiles (Q1 and Q2) on the left half of the distribution (higher proportion of CD36^+^ vasculature) included only one-third of women developing a subsequent IBC compared to the two quartiles (Q3 and Q4) on the right half of the distribution (lower proportion of CD36^+^ vasculature), which contained two-thirds of the women developing a subsequent IBC (Fig. 3*C*). In logistic regression analysis, Q3 and Q4 were associated with a higher incidence of subsequent IBC compared to Q1, with an odds ratio of 4.5 (95% CI 0.06, 2.59) and 7.87 (95% CI 1.21, 19.6), respectively (Fig. 3*D*). The cumulative incidence of IBC was 8.8% (Q1), 8.8% (Q2), 36.1% (Q3), and 43.8% (Q4) at 10 y post-diagnosis (Fig. 3*E*).

Our analysis revealed significantly lower levels of CD36 in the IBC-associated vasculature compared to the DF vasculature. CD36 expression in the vasculature surrounding DCIS lesions of cases (associated with subsequent IBC) was also significantly lower compared to controls (Fig. 3*F*). Notably, the total amount of vasculature measured (CD36 + CD31) was not significantly different between DCIS cases and controls (*SI Appendix*, Fig. S11). Collectively, these results indicated an association between CD36 loss from the vasculature surrounding DCIS lesions and the development of subsequent IBC.

### Association of CD36 Immunopositivity in the Breast Microvasculature with PPARγ Transcriptional Activity.

**Fig. 4.**
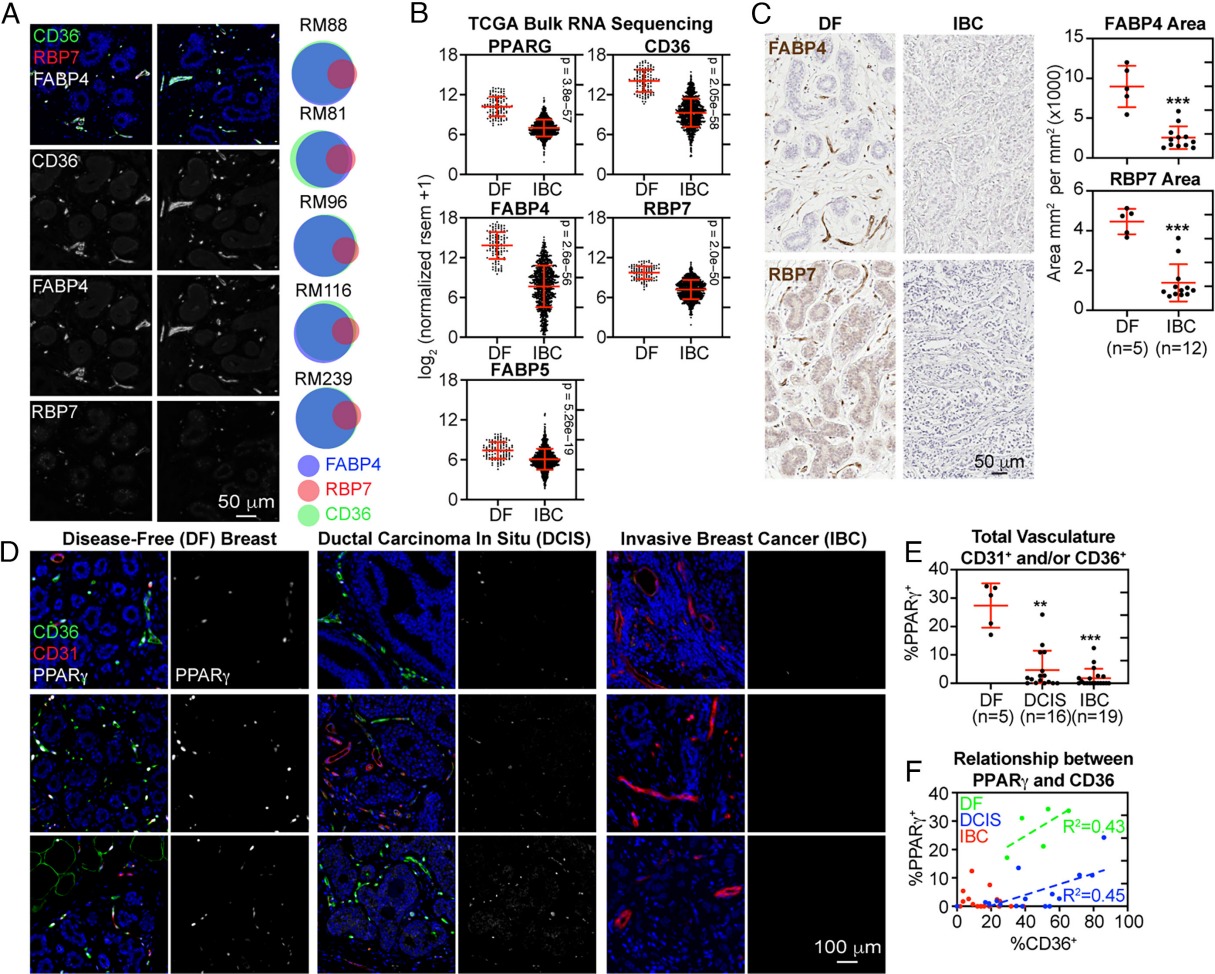
Suppression of PPARγ and its target genes in the vasculature during breast tumor progression. (*A*) FFPE sections of DF breast tissue were subjected to mIHC. For each donor tissue, cells were segmented and binned based on their positive expression of FABP4, CD36, and RBP7 as Euler diagrams. (*B*) Differential gene expression between DF breast and IBC was analyzed using bulk RNA-Seq data from TCGA. Statistical significance was determined using the Wilcoxon rank-sum test. (*C*) FFPE sections of DF breast (*n* = 5) and IBC (*n* = 12) were immunostained. The percent of the DAB^+^ area was quantified and normalized to the total area analyzed. (*D*) FFPE sections of DF breast (*n* = 5), DCIS (*n* = 16), and IBC (*n* = 19) were subjected to mIHC analysis. (*E*) For each donor, cells were segmented, and the percentage of vascular cells (CD31^+^ and/or CD36^+^) expressing PPARγ was determined. (*F*) The percentage of vascular cells expressing PPARγ (*y*-axis) and CD36 (*x*-axis). Linear regression was performed.

In our scRNA-seq analysis (Fig. 1*H*), we noted that additional PPARγ regulated genes, FABP4, FABP5, and RBP7, were coexpressed with CD36 in capillary ECs and pericytes at the transcript level. To determine whether the loss of CD36 reflected an overall dampening of the broader PPARγ program, we simultaneously evaluated CD36, FABP4, and RBP7 expression in DF human breast sections by mIHC. Following cell segmentation, immunopositivity for FABP4 and CD36 was nearly superimposed. RBP7 immunopositivity was almost exclusively observed in CD36^+^FABP4^+^ cells, however, it was more narrowly distributed (Fig. 4*A*). These data were consistent with the interpretation that CD36 expression reflected the activity of a broader PPARγ-related transcriptional program.

We next examined the integrity of the PPARγ program in tumor specimens. First, we observed that transcript levels of PPARγ, CD36, FABP4, FABP5, and RBP7 were significantly lower in malignant compared to DF breast tissues using bulk RNA sequencing data from TCGA (Fig. 4*B*). Second, as a percentage of the total area analyzed by IHC, DF breast tissue contained a significantly higher amount of FABP4 and RBP7 expression compared to IBC (Fig. 4*C*), similar to the results obtained with CD36 (*SI Appendix*, Fig. S2*A*). Third, sections of DF breast, DCIS, and IBC that were costained by mIHC for PPARγ, CD36, and CD31 showed robust PPARγ expression within the DF microvasculature components (Fig. 4*D*). In this analysis, PPARγ expression was dramatically reduced within the vasculature associated with DCIS lesions and IBC (Fig. 4 *D* and *E*, and *SI Appendix*, Fig. S12), suggesting that reduced expression of PPARγ itself determined the loss of target gene expression, including CD36 (Fig. 4*F*). In a previously published scRNA-seq analysis, PPARγ target genes were among the most down-regulated genes in ECs recovered from normal adjacent and tumor regions (16). Using the available data, we confirmed that PPARγ-regulated genes were also highly down-regulated in tumor-associated pericytes compared to pericytes recovered from normal adjacent tissue (*SI Appendix*, Fig. S13). Extending these findings, we observed dysfunction of the PPARγ program in lung and colon cancer specimens compared to DF tissues (*SI Appendix*, Fig. S14).

In summary, the reduction in CD36 expression observed in the vasculature surrounding premalignant lesions and fully invasive breast tumors coincided with a loss of PPARγ expression within the vasculature.

### Tumor-Derived Factors Repress PPARγ Expression in Stromal Cell Types.

**Fig. 5.**
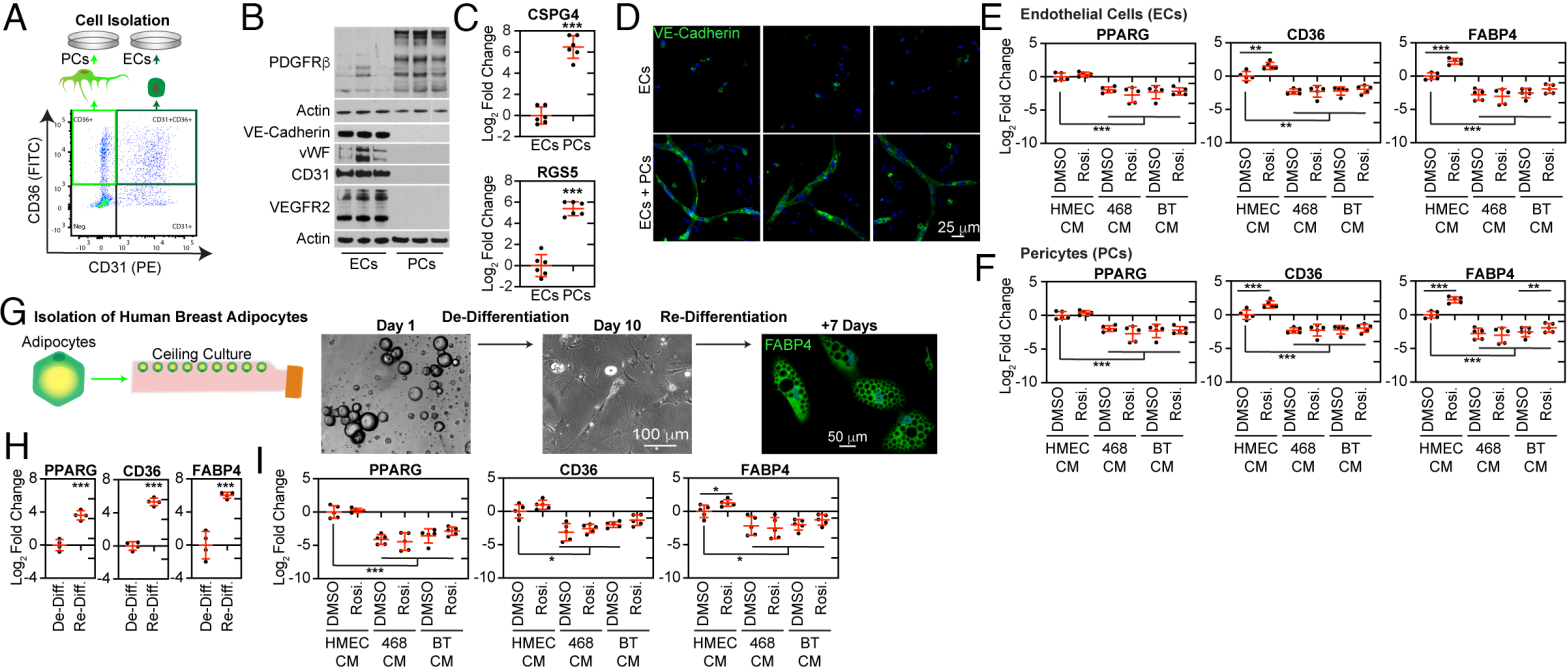
Suppression of PPARγ activity in human stromal cells by tumor cell conditioned media (CM). (*A*) Capillary ECs (CD36^+^CD31^+^) and pericytes (CD36^+^CD31^−^) populations were isolated from human breast tissues by FACS and expanded in culture for four passages (P4). (*B* and *C*) Characteristics of ECs and pericytes were assessed using (*B*) western blot and (*C*) RT-qPCR. (*D*) ECs were cultured in collagen gels for 72 h, alone or in coculture with pericytes (sourced from three donors). Subsequently, they were immunostained for VE-cadherin. (*E*) Passage 1 (P1) ECs were incubated for 72 h with CM obtained from HMECs, MDA-MB-468, or BT474 human breast cancer cells. This was done in the presence of either rosiglitazone (10 µM) or DMSO (serving as the vehicle control). Post-incubation, the cultures were analyzed via RT-qPCR for PPARG, CD36, and FABP4 expression levels. (*F*) Passage 4 (P4) pericytes were treated and analyzed in a manner similar to *E*. (*G*) Adipocytes were isolated using the ceiling culture method. While in culture, attached adipocytes underwent dedifferentiation, evident from the phase-contrast images. However, they were capable of redifferentiating, leading to the accumulation of lipid droplets. This redifferentiation was confirmed by immunofluorescence staining. (*H*) RT-qPCR was employed to determine the expression levels of PPARG, CD36, and FABP4 in undifferentiated and differentiated adipocytes. (*I*) Adipocyte differentiation was induced in the presence of CM, following the protocol described in *E*.

To study the modulation of PPARγ and its target genes in stromal cell types we first isolated ECs and pericytes from human breast tissue fragments by FACS (Fig. 5*A*). Using this technique, we detected a greater proportion of CD36^+^CD31^+^ ECs in DF breast tissues (*SI Appendix*, Fig. S15*A*) than what we expected based on our mIHC analysis (Fig. 3*A*). However, when isolated ECs were examined by immunocytochemistry, CD31 was mostly absent from FABP4^+^ capillary ECs (*SI Appendix*, Fig. S15*B*), matching the staining of intact tissue (*SI Appendix*, Fig. S15*C*). These results suggest that highly sensitive flow cytometry-based techniques were more adept at detecting lower levels of CD31 expression in capillary ECs. Notably, we observed a reduction in CD36^+^ ECs isolated from IBC specimens compared to DF breast specimens using flow cytometry (*SI Appendix*, Fig. S15 *D* and *E*), consistent with mIHC results (Fig. 3*F*).

First, we validated the identity of cultured ECs and pericytes by western blot analysis of selected EC markers, VE-Cadherin, vWF, CD31, and VEGFR2, and the pericyte/fibroblast marker, PDGFRβ (Fig. 5*B*). We were unable to identify high-quality antibodies to pericyte-specific markers. Therefore, we confirmed the pericyte-specific expression (19) of CSPG4 and RGS5 by RT-qPCR (Fig. 5*C*). CM from isolated ECs and pericytes contained a complementary set of factors required for vascular function, including ANGPT2, PDGF-BB, and EGF, which are expressed by ECs to promote vascularization and/or pericyte recruitment, and ANGPT1, HGF, and SDF-1, which are elaborated by pericytes to stabilize vascular networks (*SI Appendix*, Fig. S16*A* and Dataset S7). Notably, pericytes were the primary source of the critically important basement membrane components, collagen IV and laminin (*SI Appendix*, Fig. S16*B*). ECs were completely dependent on pericytes for in vitro tube formation in collagen matrices (Fig. 5*D*), reflecting the importance of pericyte-derived growth factors and basement membrane components in vascular organization. Remarkably, the matrix derived from pericytes facilitated proper acinar morphogenesis of breast epithelial cells (*SI Appendix*, Fig. S16 *C* and *D*).

Several cytokine and growth factor signaling pathways have been shown to negatively regulate PPARγ activity (21, 22) and repress CD36 transcription. Notably, growth factors in culture media reversibly suppressed PPARγ activity in early passage pericytes and permanently repressed PPARγ activity in early passage ECs (*SI Appendix*, Fig. S17 *A*–*G* and Datasets S5 and S6).PPARγ suppression following expansion in growth factor-containing media rendered ECs unresponsive to the antiangiogenic effect of rosiglitazone that was evident in minimally passaged ECs (*SI Appendix*, Fig. S17*H*). VEGF was necessary for EC growth (*SI Appendix*, Fig. S17*I*) and suppressed PPARγ activity in this culture system (*SI Appendix*, Fig. S17 *J* and *K*). In early passage pericytes, EGF and FGF2 were responsible for growth (*SI Appendix*, Fig. S17*L*) and the reversible suppression of PPARγ activity (*SI Appendix*, Fig. S17*M*). In ECs, PPARγ activity was incompatible with expansion in culture, such that we could not expand ECs overexpressing PPARγ (*SI Appendix*, Fig. S17 *N* and *O*), consistent with previous reports (5, 6, 9, 10).

To determine whether human tumor cells secreted factors that suppress the expression of PPARγ and activation of its target genes, CD36 and FABP4, we collected CM from human mammary epithelial cells (HMECs) and two breast cancer cell lines, MDA-MB-468 (triple-negative) and BT474 (HER2^+^) and evaluated their activity on cultured ECs and pericytes. Due to the repression of PPARγ observed during expansion in growth factor-supplemented media, we used minimally passaged ECs and pericytes and excluded supplemental growth factors. In this experiment, CM from breast cancer cell lines suppressed PPARγ and its target genes FABP4 and CD36 in both ECs (Fig. 5*E*) and pericytes (Fig. 5*F*) compared to CM from HMECs. Notably, the addition of rosiglitazone could not rescue the repression of this pathway.

In addition to ECs and pericytes, we observed robust CD36 expression in adipocytes. Although adipocytes are mostly absent from the invasive tumor microenvironment, they interact with tumor cells at the earliest stages of tumorigenesis (23). PPARγ is essential to adipocyte differentiation and cell identity. Therefore, we isolated adipocytes from DF breast tissue digests using well-established ceiling culture protocols (24). In these cultures, adipocytes attach to the cell culture surface and dedifferentiate but can be readily redifferentiated into adipocytes that accumulate lipids (Fig. 5*G*). Redifferentiation was associated with robust upregulation of PPARγ and its target genes, CD36 and FABP4 (Fig. 5*H*). In this system, PPARγ and its target genes were suppressed in the presence of CM from breast cancer cell lines compared to CM from HMECs (Fig. 5*I*). In addition, suppression of this pathway correlated with impaired lipid uptake (*SI Appendix*, Fig. S18 *A* and *B*).

Collectively, these in vitro results suggested that exposure to tumor cell-secreted factors contributed to the loss of PPARγ and its target genes observed in human breast cancer specimens (Figs. 3 and 4).

### PPARγ Suppresses the Acquisition of Protumorigenic Myofibroblastic Characteristics by Isolated Pericytes.

**Fig. 6.**
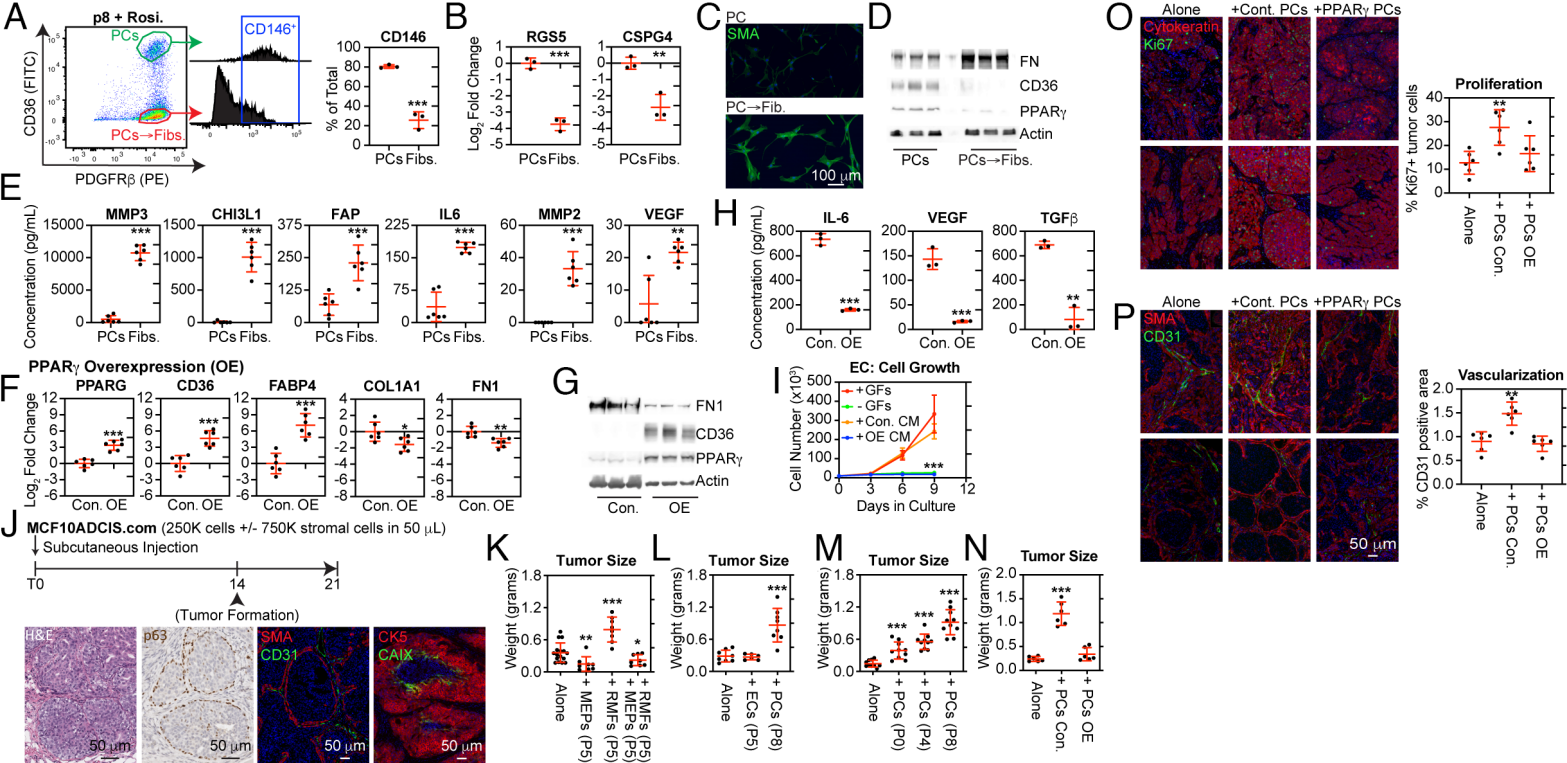
PPARγ activation suppressed protumorigenic phenotypes associated with pericyte-to-myofibroblast transition. (*A*) Pericytes isolated from the human breast vasculature were expanded for eight passages (P8), treated with 10 μM rosiglitazone for 24 h, and then fractionated into CD36^+^PDGFRβ^+^ pericytes (PCs) and CD36^−^PDGFRβ^+^ pericyte-derived myofibroblasts (PCsFibs.) using FACS. The CD146^+^ percentage was quantified. (*B*–*D*) Distinct characteristics of the populations were analyzed by (*B*) RT-qPCR, (*C*) immunocytochemistry, and (*D*) western blot. (*E*) CM collected from both populations after 24 h was analyzed by multiplex ELISA. (*F* and *G*) P4 pericytes were transduced with a lentivirus encoding human PPARγ or GFP (control) and cultured for an additional four passages (P8). PPARγ activity was validated by (*F*) RT-qPCR and (*G*) western blot. (*H*) CM was collected after 24 h from GFP control (Con.) and PPARγ-overexpressing (OE) pericytes and analyzed by ELISA. (*I*) ECs were cultured in a medium containing growth factor (+GFs), without growth factors (−GFs), with CM from control (Con. CM) or PPARγ-OE (OE CM) pericytes. (*J*) MCF10ADCIS.com tumors were characterized using H&E staining, IHC for p63, and mIHC for SMA and CD31 and for cytokeratin 5 (CK5) and Carbonic anhydrase IX (CAIX). (*K*–*N*) MCF10ADCIS.com were injected in mice by themselves (Alone) or coimplanted with a threefold excess of the indicated cell type. After 3 wk, the tumors were excised and weighed. The following cell types were cotransplanted: (*K*) myoepithelial cells (MEPs), reduction mammary fibroblasts (RMF), or a combination of both MEP and RMF; (*L*) ECs and pericytes (PCs); (*M*) uncultured (P0) pericytes directly isolated from human breast tissue, P4 pericytes, or P8 pericytes. (*N*) GFP control pericytes (P8) or PPARγ-OE pericytes (P8). (*O*) The proliferative index was determined by the percentage of cytokeratin 5 + MCF10ADCIS.com cells expressing Ki67. (*P*) Vascularization was compared between groups by determining the percentage of the tumor area expressing CD31.

The role of PPARγ in pericytes has not been extensively investigated. We observed that sustained growth factor stimulation of isolated pericytes instigated a change in cell state, which irreversibly suppressed CD36 expression, even in the presence of rosiglitazone. By passage 8, this altered population predominated. Consistent with a pericyte-to-myofibroblast transition (25), the resulting CD36^-^ cells now lacked expression of the pericyte markers CD146 (Fig. 6*A*), RGS5, and CSPG4 (Fig. 6*B*) and showed an increase in SMA^+^ filaments (Fig. 6*C*), a phenotype associated with activated myofibroblasts. Further supporting a pericyte-to-myofibroblast transition hypothesis, we found that cells lacking CD36 expression, indicating reduced PPARγ activity, deposited more ECM components, such as fibronectin (Fig. 6*D*). In addition, cells lacking CD36 elaborated greater levels of factors commonly associated with the myofibroblast phenotypes (Fig. 6*E* and Dataset S8).

The observed correlation between the loss of PPARγ activity in pericytes and the acquisition of a myofibroblast-like phenotype led us to hypothesize a role for PPARγ in opposing aspects of this transition. Constitutive overexpression of PPARγ enhanced CD36 and FABP4 transcription while decreasing transcription of fibrotic markers COL1A1 and FN1 compared to controls overexpressing GFP (Fig. 6*F*). In addition, the protein level of CD36 was increased by PPARγ overexpression, whereas the protein level of fibronectin was markedly reduced (Fig. 6*G*). Notably, we also found that long-term rosiglitazone treatment of isolated pericytes increased the expression of PPARγ target genes, CD36 and FABP4, and reduced the transcript levels of ECM components, COL1A1 and FN1 (*SI Appendix*, Fig. S19*A*). Increased CD36 and reduced fibronectin expression in rosiglitazone-treated pericytes were also confirmed at the protein level (*SI Appendix*, Fig. S19*B*). Significantly reduced IL-6, VEGF, and TGFβ levels were also observed in the CM from PPARγ-overexpressing pericytes compared to controls (Fig. 6*H*). Similar results were obtained with prolonged rosiglitazone treatment (*SI Appendix*, Fig. S19*C*). Unsurprisingly, given the dramatic difference in VEGF elaboration, CM from control populations (or growth factor supplemented medium) stimulated EC proliferation. In contrast, CM from PPARγ-overexpressing populations (or medium lacking supplemental growth factors) failed to stimulate EC proliferation (Fig. 6*I*). These findings suggest that PPARγ potentially modulates angiogenesis through its influence on the pericyte phenotype.

Analysis of decellularized ECM deposited by PPARγ-overexpressing populations demonstrated consistently lower levels and alignment of type I collagen and fibronectin fibers than controls (*SI Appendix*, Fig. S20*A*). The effect of these decellularized matrices on epithelial cell phenotype was profound. Indeed, ECM deposited by PPARγ-overexpressing pericytes could polarize MCF-10A breast epithelial cells into small acini circumscribed by α6-integrin. In contrast, ECM deposited by control pericytes (PPARγ-low) failed to polarize these cells, resulting in flat, disorganized structures and elongated cell morphologies (*SI Appendix*, Fig. S20 *B* and *C*). Significantly, the vascular basement membrane influences epithelial architecture (26). Loss of apical-basal polarity plays a significant role in tumorigenesis.

Pericytes are an important source of myofibroblasts, which can promote tumor growth in experimental models (25). Therefore, we next wanted to determine whether PPARγ activity in pericytes could limit their tumor growth-promoting potential. To answer this question, we cocultured patient-derived breast cancer organoids, PDM92 and PDM250, with either control pericytes (GFP^+^, low PPARγ expression) or PPARγ-overexpressing pericytes (PPARγ-OE). In these experiments, pericytes and epithelial cells were cultured on top of collagen gels previously polymerized within a transwell insert without supplemental growth factors for 2 wk. We found that control, but not PPARγ-OE, populations provided factors that supported the proliferation, measured by Ki67 staining, and, therefore, the expansion of both breast cancer cell models on collagen gels (*SI Appendix*, Fig. S20*D*).

To validate our hypothesis in vivo, we used the MCF10DCIS.com xenograft model, known to produce DCIS-like lesions upon subcutaneous injection in immunocompromised mice (27). These lesions are surrounded by a layer of p63/SMA myoepithelial cells and demonstrate evidence of comedo necrosis visualized by the expression of the hypoxia-related marker carbonic anhydrase IX (CAIX) in the central area of the lesions (Fig. 6*J*). We could reproduce the initially published data (27), which demonstrated that myoepithelial cells could suppress and fibroblasts could increase primary tumor growth (Fig. 6*K*). In this system, we found that coinjection of cultured pericytes but not ECs promoted the expansion of MCF10DCIS.com xenograft tumors (Fig. 6*L*). Coinjection of isolated pericytes directly sorted from human breast tissue (P0) could also enhance tumor growth, although not to the same extent as P4, or even more significantly, P8 cells, which had mostly transitioned to a myofibroblast phenotype at the time of coimplantation (Fig. 6*M*). This experiment indicated that once isolated away from the endothelium, pericytes developed a protumorigenic myofibroblast phenotype either in vitro due to constant exposure to supplemental growth factors or in the tumor microenvironment as a result of tumor cell-secreted factors.

Next, we coimplanted control pericytes (GFP^+^, low PPARγ expression, *SI Appendix*, Fig. S21) or PPARγ-overexpressing pericytes (PPARγ-OE) with MCF10DCIS.com cells. In this experiment, control populations dramatically enhanced MCF10DCIS.com tumor size, whereas PPARγ overexpressing populations led to the formation of tumors comparable in size to mice injected with MCF10DCIS.com alone (Fig. 6*N*). The proliferation index, measured by coimmunostaining for Ki67 and cytokeratin, was significantly higher in tumors formed by coinjecting MCF10DCIS.com cells with control pericytes compared to MCF10DCIS.com cells injected alone or with PPARγ-OE pericytes (Fig. 6*O*). Finally, we found that vascularization significantly increased in the MCF10ADCIS.com tumor when coinjected with control pericytes compared to PPARγ-overexpressing pericytes or no pericytes (Fig. 6*P*).

In pericytes isolated from human breast tissue, the acquisition of a myofibroblast phenotype coincided with the loss of PPARγ and its target genes (i.e., CD36), as well as alterations in the secretome. We found that activation of PPARγ transcriptional activity could oppose this transition of pericytes into myofibroblastic-like cells capable of promoting angiogenesis and the growth of breast cancer cells in vitro and in vivo.

### The Efficacy of Early Rosiglitazone Intervention in Inhibiting Tumor Growth and Progression Diminished upon the Development of a Palpable Tumor Mass.

Given that PPARγ was suppressed in stromal cell types by tumor-secreted factors in our in vitro models, we hypothesized that tumor development in a mouse xenograft model would limit the antitumor efficacy of rosiglitazone, a potent PPARγ agonist. Our initial experimental design utilized the injection of triple-negative MDA-MB-468 and HER2^+^ BT474 breast cancer cell lines into the inguinal mammary fat pads of NSG mice (Fig. 6*A*). The xenografted mice were randomized into two treatment groups. For the early intervention group, we initiated treatment with rosiglitazone or vehicle control seven days after xenotransplantation (prior to forming a palpable tumor). For the second group, we waited until the formation of palpable lesions to begin treatment. For both the MDA-MB-468 and BT474 models, early intervention with rosiglitazone significantly reduced primary tumor growth as assessed by tumor volume and tumor weight. In contrast, treatment with rosiglitazone after tumor formation did not significantly alter tumor growth (Fig. 6*B*). To obtain further insight into the inability of rosiglitazone to alter primary tumor growth, we xenografted another set of mice with MDA-MB-468 and BT474 and treated them with rosiglitazone or vehicle after the formation of a palpable tumor for five days. The tumors and contralateral mammary glands were excised and subjected to RT-qPCR analysis to measure the expression of mouse Pparg, Cd36, and Fabp4 transcripts. In this experiment, the induction of Cd36 and Fabp4 transcription by rosiglitazone was significantly inhibited in both models, suggesting impairment of the Pparg pathway within the stroma of tumor-bearing mammary glands (Fig. 6*C*).

To provide greater insight into the potential benefit of early intervention with rosiglitazone, MDA-MB-468 and BT474 cells were injected intraductally through cleaved nipples. The mice were randomized and, from the 14th day onward, received either rosiglitazone (10 mg/kg/day) or vehicle (Fig. 6*D*). We observed the formation of noninvasive DCIS-like lesions, indicated by the expansion of actively proliferating tumor cells (identified as Ki67^+^ and human-specific Lamin A/C^+^) within myoepithelial lined (SMA^+^) ducts. These lesions could progress in vivo, with progression marked by the loss of the surrounding myoepithelial layer and invasion into the mammary fat pad. We chose to analyze an early time point where discrete lesions could be clearly identified (*SI Appendix*, Figs. S22 and S23). A statistically significant reduction in the number of mammary glands with invasive tumor growth was observed for both cell models in rosiglitazone-treated mice (Fig. 6*E*).

In the vehicle-treated MDA-MB-468 controls, which contained invasive lesions large enough to evaluate adjacent stromal cells, we observed a significantly lower percentage of Pparγ^+^ adipocytes near invading tumor cells compared to unaffected regions of the mouse mammary gland (*SI Appendix*, Fig. S24*A*). These adipocytes were smaller than adipocytes in the unaffected areas of the mouse mammary gland based on analysis of H&E-stained sections (*SI Appendix*, Fig. S24*B*). Notably, MDA-MB-468 and BT474 cells did not exhibit PPARγ or FABP4 expression in the xenografts (*SI Appendix*, Fig. S25), suggesting that the antitumor activity of rosiglitazone in these models was primarily due to an effect on the mouse stroma.

Together, these experiments indicated that the antitumor activity of PPARγ ligands on the tumor stroma (9) might be limited in established tumor microenvironments, rich in secreted growth factors that repressed PPARγ.

## Discussion

**Fig. 7.**
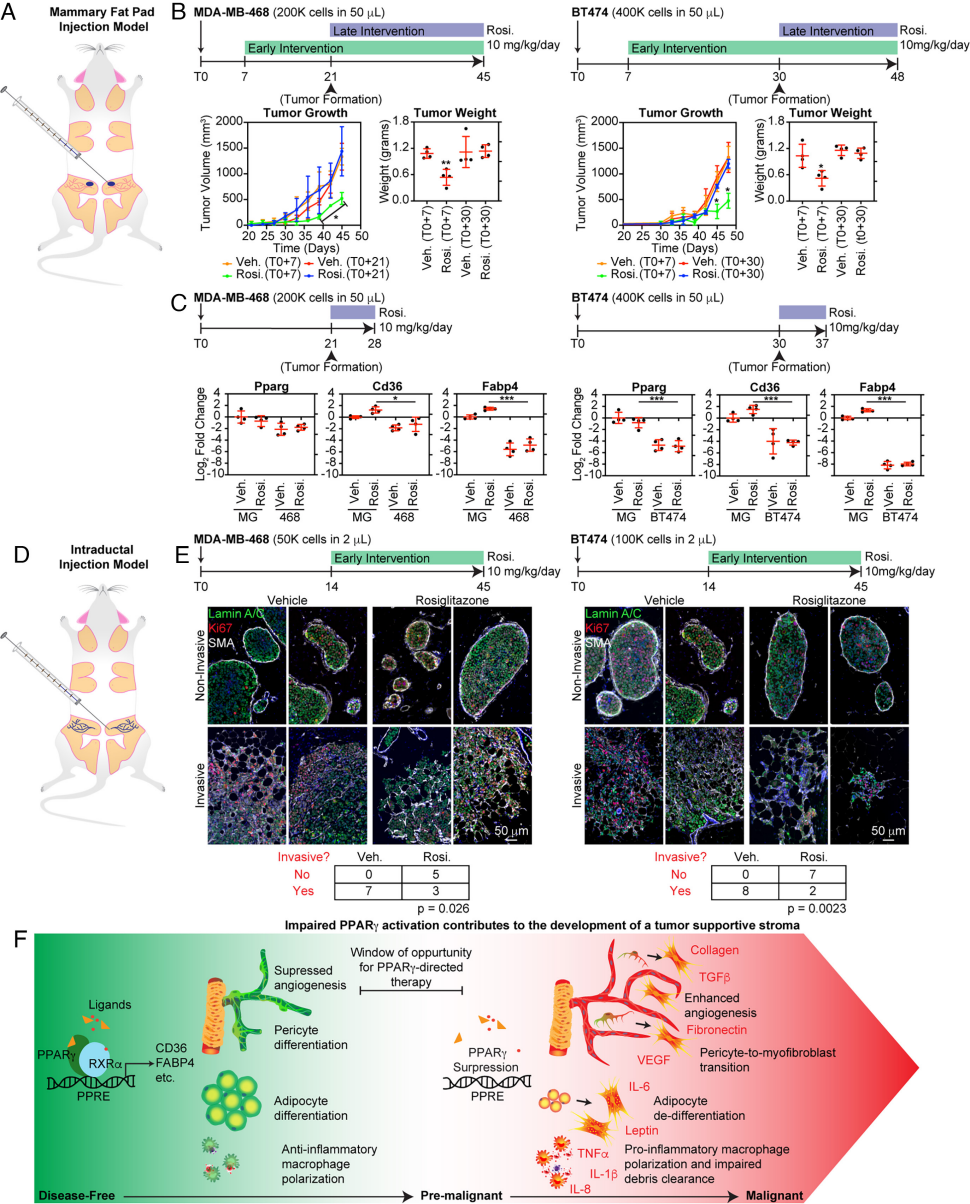
The antitumor efficacy of the PPARγ agonist rosiglitazone required early intervention. (*A*) MDA-MB-468 or BT474 cells were xenotransplanted into the mammary fat pads of NSG mice. (*B*) Rosiglitazone (10 mg/kg/day) or vehicle treatment was initiated either 7 d post-xenotransplantation, before the formation of a palpable tumor (Early Intervention), or after the formation of a palpable lesion (Late Intervention). Tumor volume (measured every other day) and final tumor weights were determined. (*C*) After forming a palpable lesion, mice were treated with rosiglitazone or vehicle for 5 d. The tumors and contralateral mammary glands were analyzed by RT-qPCR for mouse Pparg, Cd36, and Fabp4 transcripts. (*D*) MDA-MB-468 or BT474 cells were injected intraductally through cleaved nipples. Starting at day 14, the mice were randomized and treated with rosiglitazone (10 mg/kg/day) or vehicle for 31 d. (*E*) mIHC for Ki67, human-specific Lamin A/C^+^, and SMA. The number of mammary glands containing invasive events was determined by examining sections taken approximately every 200 μm through the entire tissue and compared by Fisher’s exact test. (*F*) A diagram summarizing the effect of proper (*Left*) or impaired (*Right*) PPARγ activation based on evidence from this study and previous publications.

In an effort to further illuminate the significance of CD36 repression in the development of a tumor-supportive stroma (1), we uncovered a broader impairment of PPARγ-activated transcriptional programs affecting several stromal cell types, including pericytes, ECs, and adipocytes. Factors elaborated by malignant cells mediated impaired PPARγ activity and CD36 loss, and in previous work (2), such factors were also identified from damaged cells. PPARγ ligands are known to demonstrate antitumor effects, partly through the modulation of stromal phenotypes (9, 10). Given that we observed a loss of PPARγ in stromal cell types during breast cancer progression, we hypothesized that the timing of intervention was critical to the efficacy of PPARγ-directed therapy. Indeed, early intervention with rosiglitazone was necessary in xenograft mouse models to effectively suppress tumor growth and progression. Treatment of established xenograft tumors with rosiglitazone failed to activate PPARγ target genes and no longer suppressed tumor growth. In addition, our study indicates that PPARγ plays an underrecognized role in maintaining pericyte phenotype, such that early exposure to PPARγ-directed therapy could potentially limit their contribution to the pool of tumor-supporting myofibroblasts in developing tumor microenvironments. From prior publications, prevention of adipocyte dedifferentiation into protumorigenic myofibroblast-like, precursor cells (28), inhibition of angiogenesis (9), and polarization of macrophages toward an anti-inflammatory, debris-clearing state (29) stand out as critical antitumor properties of PPARγ-directed therapy that may also be compromised during tumor progression (Fig. 7*F*). Several findings presented in this study warrant further discussion.

First, we identified specialized capillaries surrounding DF breast epithelium that robustly expressed CD36. Previous studies largely overlooked these capillaries due to their inconsistent expression of commonly utilized vascular markers such as CD31 and vWF. Although they are among the most widely used markers in histological studies, heterogeneity in the expression of CD31 and vWF is commonly observed in microvascular beds (30, 31). This observation has important implications for measures of vascular quantity or microvessel density, which have primarily been evaluated based on single markers (17). Multiplexed immunofluorescence is an invaluable tool for analyzing vessel quantity in at-risk or cancer tissues and can provide additional information about vessel type, quality, and angiogenic potential.

Since CD36 is a negative regulator of angiogenesis (5, 6), it may represent an important sensor capable of discriminating between an antiangiogenic stroma (CD36^high^) and a proangiogenic stroma (CD36^low^). This feature could have significant value in risk stratification models. Indeed, CD36 loss was more often observed in the perilesional capillaries surrounding DCIS lesions associated with subsequent IBC. However, several limitations of the DCIS cohort used in this study prevented us from interrogating the significance of CD36 expression in multivariate risk stratification models. First, the number of FFPE specimens available was small. Second, tissues were included in the study based on their case or control status and did not reflect the composition of the patient population affected by DCIS (i.e., one in seven patients progressing to IBC). Finally, we did not have information on known predictors of risk (20) for all patients. CD36 loss warrants further investigation in a cohort better suited for multivariate risk analysis.

Pericytes have many essential roles in capillary networks (32) and contribute to organ development and tissue homeostasis through interactions with the broader cellular community (26, 32). In disease states, pericytes are also an important source of myofibroblasts (33). Notably, this pericyte-to-myofibroblast transition, which follows detachment from the microvasculature and growth factor stimulation, was previously shown to promote tumor growth and progression (25). Several published studies suggest that PPARγ activation and CD36 expression in fibroblast suppresses the acquisition of a myofibroblast phenotype (1, 11, 12). However, because most studies identify any cell type that expands in a “fibroblast-specific” medium (e.g., RPMI + 10% FBS) as fibroblasts, a significant portion of these cells may be derived from pericytes rather than resident fibroblasts. In our study and a published scRNA-seq dataset derived from multiple human organs, (34) resident fibroblasts did not appear to express PPARγ.

Our findings using mouse xenograft models support the hypothesis that PPARγ activation by rosiglitazone required early intervention to achieve a reduction in tumor growth or progression. In murine models of cancer, depleting PPARγ from the adipocyte, immune, endothelial, and perivascular compartments animal is an attractive next step toward understanding the relative contribution of stromal cell types to the antitumor effect of PPARγ activation (13). Our results do not challenge previous studies reporting the antitumor efficacy of PPARγ ligands. However, several notable differences in experimental design should be noted when comparing findings between studies. First, we chose human cancer cell lines that did not demonstrate PPARγ expression or a response to rosiglitazone treatment in order to concentrate on stromal effects in vivo. Previous studies have achieved antitumor effects by altering tumor cell differentiation (8), which was not considered in our study. Second, we used orthotopic injections, which exposed tumor cells to a different initial mixture of host stromal cells compared to the subcutaneous injections performed in other studies (7–9). Finally, we used immunocompromised mice. Therefore, the effect of PPARγ on the antitumor activity of immune cells was not considered.

In this study, while we concentrated on genes directly regulated by PPARγ through canonical PPREs, such as CD36 and FABP4, it's worth noting several limitations. PPARγ binding sites are often located outside proximal gene promoters and are not necessarily associated with canonical PPREs. In addition, PPARγ often affects gene transcription by (1, 11) modifying the activity of other transcription factors, such as NF-κB (29). Therefore, another important future effort is to map PPARγ binding sites in the genomes of adipocytes, pericytes, and ECs to develop a more comprehensive understanding of how PPARγ differentially regulates gene transcription in each cell type.

In conclusion, factors present in the tumor microenvironment can reprogram the tissue stroma toward a protumorigenic state. As a result of this reprogramming, PPARγ expression, and thus CD36 expression, was almost completely lost. The generality of this observation is important. PPARγ-regulated transcriptional networks are impaired in several tissues, giving rise to multiple tumor types, including breast, colon, and lung cancer. PPARγ loss undermines the therapeutic potential of PPARγ ligands (9). In the relatively few clinical trials published, synthetic PPARγ ligands do not demonstrate significant therapeutic benefits in cancer patients (35). Our findings suggest that synthetic agonists may demonstrate more significant therapeutic benefits in cancer-prone individuals at high risk of developing cancer prior to a tipping point that leads to the irreversible repression of PPARγ and the development of a supportive tumor microenvironment. Alternatively, the identification of therapeutic strategies designed to reactivate PPARγ could contribute to the maintenance of adipocyte phenotype and the normalization of intratumoral vascular networks by opposing proangiogenic signals and thus reducing endothelial proliferation as well as limiting the tumor-supportive phenotype associated with pericyte-derived myofibroblasts.

## Materials and Methods

### Human Specimens.

All specimens were acquired with patient consent, studied under Institutional Review Board-approved protocols # 10-01532 and 10-01496, and deidentified prior to use in this study. The Cooperative Human Tissue Network (CHTN) Western Division, Nashville, TN, and Kaiser Foundation Research Institute, Oakland, CA, provided DF breast tissue specimens obtained from women undergoing reduction mammoplasty. The UCSF Department of Pathology and CHTN provided IBC specimens. DCIS specimens and outcome data were part of a previously described retrospective cohort (20).

### Imaging.

mIHC was performed using Tyramide Signal Amplification (Akoya), imaged using the Nuance FX multispectral imaging system (PerkinElmer), and analyzed with InForm (Version 2.1; PerkinElmer) or imaged with BZ-X800 (Keyence) analyzed with ImageJ (version v2.1.0/1.53c) and Qupath (v0.2.0-m3). For confocal imaging, tissue was delipidated using the ScaleCubic protocol, immunostained, cleared with Rapiclear 1.52 (SunJin Lab), and imaged at 1,024 × 1,024 at the optimized z-resolution using Leica SP8 laser scanning confocal microscope and LASX software.

### Stromal Cell Isolation and Culture.

Human breast tissue was enzymatically dissociated with 200 U·ml^−1^ Collagenase Type-2 (Worthington, Newark, NJ) and 100 U·ml^−1^ hyaluronidase (Sigma-Aldrich) and dissociated to single cells using 5 U·ml^−1^ Dispase and 1 μg·ml^−1^ DNAse I (Stem Cell Technologies) and then 0.05% trypsin–EDTA. Unique lipid-modified oligonucleotides (LMOs) barcodes were added to distinguish donors in the scRNA-seq analysis. CD36^+^ and CD31^+^ cells were subjected to scRNA-seq using 10X Genomics Single Cell V2 platform and sequenced on an Illumina HiSeq4500. ECs and pericytes were isolated by FACS based on their expression of CD36 and CD31, then cultured in EGM2-MV medium (Lonza) with 1 mg·ml^−1^ Primocin (Invivogen). Adipocytes were isolated using the ceiling culture (24) method in DMEM/F12 with 1x GlutaMAX, 10% FBS, and 1 mg·ml^−1^ Primocin and redifferentiated in vitro using PGM-2 Preadipocyte Growth Medium-2 (Lonza).

### Mouse Xenografts.

MCFDCIS.com cells (250,000) were injected subcutaneously into 7- to 10-wk-old female NSG mice alone or together with a threefold excess (750,000) of myoepithelial cells (MEPs), reduction mammoplasty fibroblasts (RMF), pericytes or ECs in 50% Matrigel. The tumors were allowed to grow for 3 wk. MDA-MB-468 cells (200,000) and BT474 cells (400,000) were injected into the mammary fat pad of 6- to 8-wk-old NSG mice in 50% Matrigel. Rosiglitazone was administered intraperitoneally at 10 mg/kg/day in 10% DMSO, 40% PEG-300, 5% Tween-80, and 45% 0.9% Saline. MDA-MB-468 cells (50,000) and BT474 cells (100,000) were injected intraductally into 5- to 6-wk-old NSG mice using a Hamilton syringe after first cutting the nipple. The cells were injected in 2 μL of PBS containing 0.9% trypan-blue. Xenograft tumors were excised, weighed, and formalin-fixed and paraffin-embedded. Protocols were approved by the University of California, San Francisco—Institutional Animal Care Use Committee.

### Statistical Analysis.

Pairwise comparisons were performed using the Welch’s T test (GraphPad Prism Software) unless otherwise indicated in the figure legend. Levels of significance used were *<0.01, **<0.001, and ***<0.0001. We applied logistic regression to determine odds ratios (ORs) using the Survival package (version 2.44-1.1) in R (version 3.6.1). Cell Ranger software (10x Genomics, version 1.31), deMULTIplex package (version 1.0.2), and Seurat R package (version 1.2) in R (version 3.6.1) were used for scRNA-seq analysis.

A detailed description of the procedures used in this study is included in *SI Appendix*, *Supplementary Materials and Methods*.

## Supplementary Material

Appendix 01 (PDF)

Dataset S01 (XLSX)

Dataset S02 (XLSX)

Dataset S03 (XLSX)

Dataset S04 (XLSX)

Dataset S05 (XLSX)

Dataset S06 (XLSX)

Dataset S07 (XLSX)

Dataset S08 (XLSX)

Dataset S09 (XLSX)

## Data Availability

scRNA-seq data have been deposited in Gene Expression Omnibus (GSE190592) (36).
