# Supplementary material for: Loss of PPARγ activity characterizes early protumorigenic stromal reprogramming and dictates the therapeutic window of opportunity
Source: Proc Natl Acad Sci U S A. 2023 Oct 10;120(42):e2303774120. doi: 10.1073/pnas.2303774120 (PMC10589683; doi:10.1073/pnas.2303774120)
Supplement: Supplementary file 1 — Appendix 01 (PDF) [file pnas.2303774120.sapp.pdf]

PNAS

Supplementary Information for

Loss of PPARγ activity characterizes early pro-tumorigenic stromal reprogramming and dictates the therapeutic window of opportunity.

Authors: Joseph A. Caruso[1*], Xianhong Wang[1], Lyndsay M Murrow[2], Carlos Ivan Rodriguez[1], Chira Chen-Tanyolac[1], Lisa Vu[3], Yunn-Yi Chen[1], Philippe Gascard[1], Zev J Gartner[2], Karla Kerlikowske[3] and Thea D. Tlsty[1*]

Authors' Affiliations: Departments of (1) Pathology, (2) Pharmaceutical Chemistry, (3) Medicine and Epidemiology and Biostatistics, University of California San Francisco, San Francisco, CA 94143.

*To whom correspondence may be addressed. Email: Thea.Tlsty@ucsf.edu; Joseph.Caruso2@ucsf.edu

This PDF file includes:

Supplemental Materials and Methods
Supplementary Figures 1 to 27

Other supplementary materials for this manuscript include the following:

Datasets 1 to 9

Supplemental Materials and Methods:

Reagents were obtained from ThermoFisher Scientific unless otherwise stated. Antibodies utilized for each application were listed in Supplementary Dataset 9.

Immunohistochemistry: Sections (5 µm thick) were cut from FFPE tissue blocks and placed on positively charged Superfrost Plus microscopy slides. The slides were baked at 60ºC overnight, deparaffinized in two changes of xylene, and rehydrated in graded ethanols (100%, 100%, 95%, 85%, 70%) to distilled H_2O for five minutes at each step. Endogenous peroxidases were quenched with 3% H_2O_2 (Sigma-Aldrich) diluted in phosphate-buffered saline (PBS). Heat-induced antigen retrieval was performed in citrate buffer pH 6.0 (Sigma-Aldrich) at 95ºC for 15 minutes. Non-specific antibody binding was blocked using Background Sniper (Biocare Medical). The tissue sections were incubated for one hour at room temperature (RT) with each primary antibody in 1% BSA and 30 minutes in pre-diluted MACH 2 conjugated anti-mouse or anti-rabbit secondary antibody (Biocare Medical). The slides were washed in TNT buffer (0.1 M TRIS-HCL pH 7.5, 0.15M NaCl, and 0.05% Tween-20) following blocking and three times for five minutes each after applying both primary and secondary antibodies. For standard IHC, the slides were developed with DAB substrate (GeneMed) for three minutes, washed in distilled H_2O, counterstained with Mayer's hematoxylin (Sigma-Aldrich), rinsed in a solution of 0.2% ammonia in water for 1 minute to blue the hematoxylin stain, and mounted with Permount. For mIHC, the signal was developed using Tyramide Signal Amplification (TSA) solution (Akoya): FITC (two minutes), Cy3 (three minutes), or Cy5 (seven minutes). To facilitate multiplex staining, the antibody complex was removed by heating in 95ºC citrate buffer pH 6.0 for 5 minutes. Nuclei were counterstained with 3 µM DAPI in PBS for 5 minutes, washed in distilled H_2O, and mounted with Vectashield HardSet Mounting Medium (Vector Labs). Standard IHC-stained slides were imaged (20x) using an Aperio AT2 whole slide scanner (Leica). Analysis was performed using Qupath software (v0.2.0-m3) (1). Positive pixel count was performed using a threshold for DAB signal based on primary antibody omitted controls. Slides stained with the CD36, CD31, and vimentin mIHC module were imaged using an Eclipse Ti microscope (Nikon Instruments). Fluorescence spectra were recorded from 420 nM to 720 nM at 20 nM increments with a Nuance FX multispectral imaging system (PerkinElmer) using Nuance software (PerkinElmer, version 3.0.2). For analysis of these multiplex IHC-stained slides, a spectral library was generated by collecting fluorescence spectra from slides singly stained with each fluorophore and then subtracting the background fluorescence spectra (generated from an unstained slide). Spectral processing using this library was performed with the InForm™ software (Version 2.1; PerkinElmer), which separated DAPI, FITC, Cy3, and Cy5 signals. At least five fields were analyzed from each specimen to determine positive pixel count. In addition, some mIHC-stained slides were imaged using a BZ-X800 fluorescence microscope (Keyence). Stitching of overlapping 20x fields was performed using the BZ-X800 analyzer. Images were prepared for analysis using Image J (version 2.1.0/1.53c). Two approaches were used to analyze these images in Qupath: (1) Positive pixel count was performed using fluorescence intensity thresholds based on primary antibody omitted controls. (2) Cells were segmented based on DAPI staining using the built-in watershed-based cell segmentation algorithm. Individual classifiers were trained for each marker and combined to create a composite classifier for scoring cells within the annotated regions.

Hematoxylin and Eosin (H&E) Staining: As described for IHC, sections of 5µm thickness were mounted on glass slides, deparaffinized in xylene, and rehydrated through a descending series of ethanol solutions (100%, 100%, 95%, 85%, 70%) to distilled H_2O. The rehydrated sections were stained with Harris Hematoxylin solution (Sigma-Aldrich) for 10 minutes. The slides were rinsed in running tap water for 5 minutes. After ten dips in 95% ethanol, the slides were counterstained with 1% Eosin Y solution (Sigma-Aldrich) for one minute. The tissue sections were then dehydrated in ethanol (95%, 95%, 100%, and 100%) and cleared in xylene. Finally, the slides were then mounted using permount and coverslipped.

Confocal imaging: Tissues were cut into 2 mm^3 pieces and fixed in 4% paraformaldehyde (Electron Microscopy Sciences) for 24 hours at 4ºC and washed for 6-12 hours in PBS containing 0.1% Tween-20 (PBST) at RT. For frozen sections, the fixed tissue was cryopreserved in 30% Sucrose (Sigma-Aldrich) for 72 hours at 4ºC, then frozen in optimal cutting temperature (O.C.T.) using a dry ice-methanol bath, stored at -80ºC, and sectioned to 100-200 µm using a Leica CM 1950 cryostat. The tissue was delipidated using ScaleCubic one reagent one, a mixture of 15% Triton, 25% N,N,N′,N′-Tetrakis(2-

Hydroxypropyl)ethylenediamine (Sigma), 25% Urea and 35% ddH2O (2). The tissue was first incubated at 37 °C in ScaleCubic-one reagent one diluted 1:1 in ddH_2O for 12 hours (one hour for frozen sections), followed by undiluted ScaleCubic-one reagent one for three days (one hour for frozen sections), changing daily. Note that the ScaleCubic-one reagent one was required for CD36 immunoreactivity, and other methods tested, lacking this delipidation step, produced inferior signal intensity. After 6-12 hours of washing in PBST, non-specific binding was blocked with 10% donkey serum, 0.3M glycine, and 1% Triton X-100 in PBS for 8 hours (1 hour for frozen sections) at 4^oC. Primary antibodies were applied for 72 hours (O/N for frozen sections) in 1% donkey serum and 0.2% Triton X-100 in PBS. Secondary donkey anti-mouse 488 and anti-rabbit 555 diluted 1:250 were applied in the same diluent for 48 hours (O/N for frozen sections). Following antibody staining steps, the tissue was washed for 6-12 hours in PBST. DAPI (10 μg/mL in PBS) was added for three hours at room temperature (30 minutes for frozen sections). The tissue was cleared in Rapiclear 1.52 (SunJin Lab) for 24-48 hours (one hour for frozen sections), mounted with 0.25 - 3 mm spacers (SunJin Lab), and coverslipped. Note that the Alexa488 fluorophore was only stable in Rapiclear 1.52 for one day, then progressively faded and formed bright aggregates. Three-dimensional confocal imaging was performed using a Leica SP8 laser scanning confocal microscope. Tile scans of Z-stacks were acquired at a resolution of 1,024 x 1,024 using the optimized Z resolution determined by the software. Imaging was performed with a 20x 0.95 NA or a 63x 1.2 NA objective. Image stacks were merged and processed using LASX software (Leica Microsystems).

Fluorescence-activated cell sorting (FACS): Human breast tissue was minced and enzymatically dissociated in Roswell Park Memorial Institute medium (RPMI)-1640 with 2.1 mM L-glutamine and 25 mM HEPES supplemented with 10% (v/v) fetal bovine serum (FBS; Atlanta Biologicals), 100 $U \cdot ml^{-1}$ penicillin, 100 $mg \cdot ml^{-1}$ streptomycin, 0.25 $mg \cdot ml^{-1}$ fungizone, 50 $\mu g \cdot ml^{-1}$ gentamycin, 200 $U \cdot ml^{-1}$ Collagenase Type-2 (Worthington, Newark, NJ) and 100 $U \cdot ml^{-1}$ hyaluronidase (Sigma-Aldrich) at 37°C for 12-16 h. The digest was centrifuged at 300 x g for 10 min and washed with RPMI 1640 + 10% FBS. Large epithelial-enriched structures (referred to as tissue organoids) were recovered by filtration through a 150 μm nylon mesh filter. Tissue organoids were dissociated to single cells using first 5 $U \cdot ml^{-1}$ Dispase containing 1 $\mu g \cdot ml^{-1}$ DNAse I (Stem Cell Technologies) for 5-10 minutes and then 0.05% trypsin-EDTA for 5-10 minutes. The cells were washed with PBS + 1% BSA, filtered through a 40-μm cell strainer, resuspended at a concentration of $1x10^6$ cells in 100 uL of PBS + 1% BSA, and stained for 15 minutes at room temperature with fluorophore and/or biotin-conjugated antibodies. The cells were again washed with PBS + 1% BSA and stained for 15 minutes at room temperature with BV785-streptavidin (concentration: 1:100; Biolegend) and 0.5 $\mu g \cdot ml^{-1}$ DAPI. The cells were again washed, resuspended at $1x10^6$ in 100 μL PBS + 1% BSA, filtered through a 40-μm cell strainer, and loaded onto a FACS Aria II Cell Sorter (Becton Dickinson) fitted with a 100 μm nozzle. After gating out debris, doublets, dead ($DAPI^+$), and unwanted cell types (streptavidin-BV785), the cells expressing the desired immunoprofiles were collected into a 1.5 mL tube containing 0.5 mL cell culture media. A similar protocol was followed for analytical flow cytometry analysis, except the cells were collected from tissue culture plates by incubating them for 10-15 minutes with TrypLE express and analyzed using LSRFortessa flow cytometer (Becton Dickinson).

MULTI-seq: Cell dissociation was performed as described above. Unique lipid-modified oligonucleotides (LMOs) barcodes (3) were stably incorporated into the plasma membrane of dissociated human breast cells from 14 donors by first incubating cells with 10X Anchor-Barcode for 5 minutes and then Co-Anchor for five minutes in basal media. Cells were pooled and prepared for FACS as described above, except that the antibody staining was performed on ice for 30 minutes instead of room temperature for 15 minutes to preserve the integrity of the LMO barcode labeling. Barcoded cells expressing CD31 and/or CD36 were sorted into a single tube. Single-cell cDNA libraries were prepared using the 10X Genomics Single Cell V2 platform as described by the manufacturer, except for adding 1 μL 2.5μM MULTI-seq primer to the cDNA amplification master-mix. The library concentration was quantified using a DNA Bioanalyzer chip (Agilent) and the Illumina Library Quantification Kit (Kapa Biosystems) and sequenced on one lane of an Illumina HiSeq4500 sequencer with an average depth of 21,805 reads/cell. The Cell Ranger software (version 1.31) was utilized for sample pre-processing, including cell barcode classification, quality control, sequence alignment (human reference genome GRCh37), and counting of unique molecular identifiers (UMIs). The deMULTIplex package (version 1.0.2) in R (version 3.6.1) was utilized for sample barcode classification. The Seurat R package (version 1.2) was used to filter, reduce dimensionality, unsupervised clustering, and identify differentially expressed genes (4). Genes had to be

expressed in at least three cells for inclusion in the analysis. In the distribution of genes detected per cell, outliers greater than four standard deviations from the mean and cells with less than 750 identified genes were removed. Cells with greater than 10 percent of counts aligned to the mitochondrial genome were also removed. Following filtering, the feature expression measurements for each cell were normalized to the total expression, multiplied by a scale factor of 10,000 (default), and log-transformed. Principal component analysis was performed on the 2,000 genes with the highest standardized variance. As determined by visual inspection of the elbow plot, the first 13 principal components explained most variance in the data. Significant principal components were utilized to cluster single-cell data using the KNN (k-nearest neighbor) algorithm at a resolution of 0.1. For visualization, the data were subjected to dimensional reduction using the Uniform Manifold Approximation and Projection (UMAP). Clustering was performed based on a shared nearest neighbor modularity optimization algorithm implemented through Seurat's FindClusters function. Clusters enriched for epithelial and fibroblast markers were removed from downstream analysis. Differential gene expression analysis was performed using the *FindMarkers* function in Seurat using the default non-parametric *Wilcoxon rank-sum test.*

Immunofluorescence: Cells were cultured on four-well glass chamber slides (Millipore). The cells were fixed using 4% paraformaldehyde in PBS for 15 minutes at room temperature and permeabilized for 10 min in PBS containing 0.25% Triton X-100. Non-specific binding was blocked with 10% donkey serum, 0.3M glycine, and 0.1% Tween-20 in PBS for 1 hour. Primary antibodies were applied overnight in 1% donkey serum and 0.1% Tween-20 in PBS. Secondary donkey anti-mouse 488 and anti-rabbit 555 diluted 1:500 were applied in the same diluent for 1 hour. DAPI (0.2 µg/mL in PBS) was added for 5 minutes at room temperature. The slides were washed between each step in PBS containing 0.1% Tween-20 following antibody staining steps. Images were acquired using a Leica SP8 laser scanning confocal microscope at a resolution of 1,024 x 1,024 and processed using LASX software (Leica Microsystems) or using a BZ-X800 fluorescence microscope (Keyence) and processed using Image J (version 2.1.0/1.53c).

Quantitative PCR: Total RNA was isolated from cells and DNase I was treated using the RNeasy mini kit (Qiagen). cDNA synthesis was performed using the High-Capacity cDNA Reverse Transcription Kit. Quantitative PCR was performed on a CFX-96 (Biorad) thermocycler using 2x SsoFast Master Mix (Bio-Rad Laboratories) and analyzed by the $2^{-\Delta\Delta CT}$ method (5). The primer-probe set for human transcripts: COL1A1 (Hs00164004_m1), FN1 (Hs01549976_m1), CSPG4 (Hs00361541_g1), RGS5 (Hs01591223_s1), PPARG (Hs01115513_m1), CD36 (Hs00354519_m1), and FABP4 (Hs01086177_m1). The primer-probe set for mouse transcripts: Cd36 (Mm00432403_m1), Pparg (Mm00440940_m1), and Fabp4 (Mm00445878_m1). For normalization of human samples: GUSB: Forward Primer: CTCATTTGGAATTTTGCCGATT, Reverse Primer: CCGAGGAAGATCCCCTTTTTA, and Probe: FAM-TGAACAGTCACCGACGAGAGTGCTGGTA-TAM from Integrated Device Technology (IDT). For normalization of mouse samples: Gapdh (Mm99999915_g1). The expression of GUSB or Gapdh was used to normalize variances in input cDNA.

Western Blot: Cells were washed with PBS and lysed using radioimmunoprecipitation assay buffer (RIPA buffer) containing the HALT protease and phosphatase inhibitor cocktail. For each sample, a cell extract (corresponding to 50 µg of protein as determined by a Micro BCA Protein Assay Kit) was prepared in NuPAGE LDS Sample Buffer (4X) with Reducing Agent (10X), heated at 95°C for 15 minutes and electrophoresed in each lane of a NuPAGE 4-12% Bis-Tris or 7% Tris-Acetate (for larger proteins) gel along with a BenchMark Pre-stained Protein Ladder. Proteins were transferred onto a nitrocellulose membrane (Biorad, Hercules, CA) overnight at 4°C at 36 mV. The membrane was washed in TBS-T (0.05 M Tris-HCl pH 7.5, 0.15M NaCl, 0.05% Tween-20), blocked for one hour at room temperature in blotto (5% nonfat dry milk in TBS-T), incubated with primary antibodies overnight at 4°C, washed in TBS-T, incubated with goat anti-rabbit horseradish peroxidase conjugate secondary antibody (Jackson Immunoresearch) 1:5000 in Blotto for one hour, washed in TBS-T and developed with ECL Western Blotting Substrate. A chemiluminescent signal was detected by exposing CL-XPosure Film to the membranes (the blots with the gray-colored backgrounds) or using the KwikQuant Digital Western Blot Detection System (the blots with the white backgrounds). Images of Western blots are shown in Figures S26-S29. The blots with gray backgrounds were scanned from the film, and those with white backgrounds were imaged with the KwikQuant system.

Bulk RNA sequencing: Total RNA was isolated from cells and DNase I was treated using the RNeasy mini kit (Qiagen). RNA concentration was determined using a Qubit Fluorometer and RNA Assay Kit. RNA quality was measured using a Bioanalyzer 2100 and RNA chips (Agilent). For samples demonstrating an RNA Integrity Number (RIN) >7, mRNA was enriched using poly-T oligonucleotide-coated magnetic beads (New England Biolabs, Ipswich, MA). Sequencing libraries were generated using a NEBNext Ultra™ RNA Library Prep Kit (New England Biolabs). An AMPure XP system (Beckman Coulter, Brea, CA) was utilized for fragment selection (150-200 bp) and purification of PCR-amplified libraries. Final library quality was assessed using a Bioanalyzer 2100 DNA chip. The libraries were sequenced on an Illumina HiSeq 4000 platform, generating 150 bp paired-end reads. Reads were aligned to the human reference genome GRCh37 using STAR (v2.5). Quantification of read numbers was mapped to each gene by HTSeq v0.6.1 in FPKM (fragments per kilobase of exon model per million mapped reads). Differential gene expression analysis was performed using DESeq2 (v2_1.6.3). The p-values were adjusted using Benjamini and Hochberg's False Discovery Rate (FDR).

Cell Culture: Dissociated human breast tissue (digested with Collagenase Type-2 and hyaluronidase as described above) was used to isolate endothelial cells (ECs) and pericytes (PCs) by FACS. Doublets, dead cells (DAPI$^+$), and immune cells (CD3$^+$, CD4$^+$, CD8$^+$, CD16$^+$, CD64$^+$ and/or CD45$^+$) were removed and cells staining positive for CD31 and/or CD36 were isolated. For most experiments, ECs and PCs were sorted directly from the tissue into a 6-well plate based on their expression of CD36, cultured together in EGM2-MV medium (Lonza) with 1 mg ml^{-1} Primocin (Invivogen) for 7-10 days, and finally separated based on their expression of CD31 (ECs: CD31$^+$ and PCs: CD31$^-$). We were able to achieve the greatest purity using this method. Of note, relative to ECs, PCs expanded more rapidly in culture; therefore, achieving high purity was particularly important for EC cultures. To obtain early passage ECs, tissue fragments were cultured in bulk (all cell types) on 75 cm^2 flasks for 5-7 days in EGM2-MV with 1 mg ml^{-1} Primocin. From these bulk cultures, ECs were isolated based on their expression of both CD36 and CD31, then seeded into six-well plates containing EGM-2 MV medium (Lonza) and used after 5-7 days of additional culture (Passage 1) or transferred to a P75 flask and cultured until 70% Confluent (Passage 2). ECs and PCs were passaged using TrypLE Express. In experiments requiring growth factor removal, EBM-2 (basal media; Lonza) was supplemented with 1% FBS, R3-IGF-1, ascorbic acid, and hydrocortisone, omitting FGF2, FGF, and VEGF. Rosiglitazone (Sigma Aldrich) was dissolved in DMSO (Sigma Aldrich) and added to the media at a final concentration of 10 μM. Purified virus containing PPARγ (Accession: BC006811), and GFP cDNA was obtained from Applied Biological Materials and transduced into target cells according to manufactures instructions. Cells were selected with blasticidin at 10 μg ml^{-1}.

Adipocytes were isolated by skimming off the white adipocyte layer from the digested breast tissue and culturing the cells in DMEM/F12 with 1x glutamax, 10% FBS, and 1 mg ml^{-1} Primocin. The flask was filled with media and turned upside down in the incubator so that the floating cells (adipocytes) could attach to the tissue culture-treated surface of the flask, referred to as ceiling culture (6). Over ten days, the adipocytes attached/de-differentiated, and the flasks were flipped to their normal side. The isolated pre-adipocytes were redifferentiated by culturing cells in PGM-2 Preadipocyte Growth Medium-2 from Lonza. HCS LipidTOX Green Neutral Lipid Stain was used for staining according to the manufacturer's instructions, and the cells were imaged using a BZ-X800 fluorescence microscope (Keyence). Mean pixel intensity was qualified using Image J (version 2.1.0/1.53c).

PDM-92 and PDM250 breast tumor organoids (American Type Culture Collections, ATCC) were resuspended in 50 μL of 10 mg·ml^{-1} matrigel (Corning) and solidified on prewarmed 24-well culture plates (Corning) for 15 minutes at 37°C. After solidification, 500 μL of epithelial media containing 20 ng·ml^{-1} Heregulin β-1, 5 ng·ml^{-1} FGF 7, 20 ng·ml^{-1} FGF10, 1 ng·ml^{-1} EGF (Peprotech), 500 nM A83-01, 5 mM Y-27632, 500 nM SB202190 (Tocris), 1.25 mM N-Acetylcysteine, 5 mM Nicotinamide (Sigma), 1x B27 supplement, 1x Glutamax, 1x HEPES and 100 mg·ml^{-1} Primocin in Advanced DMEM/F12. Organoids were passaged by mechanical shearing with TrypLE.

MCF-10A cells were cultured in DMEM/F12 containing 5% horse serum, 20 ng ml^{-1} EGF, 0.5 μg ml^{-1} hydrocortisone, 100 ng ml^{-1} cholera toxin, 10 μg ml^{-1} insulin, 100 units ml^{-1} penicillin, 100 mg ml^{-1} streptomycin.

MCF-10ADCIS.com cells were cultured DMEM/F12 containing 5% horse serum, 100 units ml^{-1} penicillin, and 100 mg ml^{-1} streptomycin.

BT474 and MDA-MB-468 cells were cultured in Eagle's Minimum Essential Medium, 0.1 mg·ml^{-1} human recombinant insulin, 1x HEPES, and 10% FBS.

Human mammary epithelial cells (HMECs) and myoepithelial cells (MEPs) were expanded in 20 ng·ml^{-1} Ampheregulin, 5 ng·ml^{-1} FGF 7, (Peprotech), 500 nM A83-01, 5 mM Y-27632, 500 nM SB202190 (Tocris), 1.25 mM N-Acetylcysteine, 5 mM Nicotinamide (Sigma), 1x B27 supplement, 1x Glutamax, 1x HEPES and 100 mg·ml^{-1} Primocin in Advanced DMEM/F12. MEPs were sorted from human breast tissue using FACS. Doublets, dead cells ($DAPI^+$), and immune cells ($CD3^+$, $CD4^+$, $CD8^+$, $CD16^+$, $CD64^+$, and/or $CD45^+$) were removed, and cells staining negative for EpCAM and positive for both CD10 and CD49f were isolated.

Reduction mammary fibroblasts (RMF) were cultured in FGM-2 medium from Lonza supplemented with 100 mg·ml^{-1} Primocin (Invivogen). Human breast tissue fragments were adhered to tissue culture flasks. FGM-2 promotes preferential fibroblastic outgrowth.

The identity of tumor cell lines was validated by Short Tandem Repeat (STR) analysis before freezing stocks in liquid nitrogen. Cell number was calculated using a Countess Automated Cell Counter. All cells were maintained in a humidified tissue culture incubator at 37°C and 6.5% CO_2.

In vitro Tubular Network Formation: The surface of a 24-well plate was coated with 100 µL of reduced growth factor BME (Trevigen) diluted to 10 mg/ml with basal EBM-2 medium. ECs or PCs were cultured in basal medium with 1% FBS for 24 hours, then labeled with 1 µM Calcein AM in basal medium containing 0.1% BSA for 15 minutes at 37°C, collected using TrypLE Express, filtered through a 40-µm cell strainer, and transferred to the BME-coated plates in basal medium containing 1% FBS for 12 hours. The cellular networks were imaged using the 4x objective of a Nikon Eclipse TE300 Inverted Microscope and the NIS elements software (Nikon). The images were converted to binary using ImageJ (version 1.52a) and analyzed with the Angiogenesis Analyzer plugin (7).

Vasculogenic Assay: Type I collagen (Advanced Biomatrix) diluted from a 3.8-4 mg/mL stock solution to 2.5 mg/mL with sterile water, and 10x M199 was prepared and neutralized with the 1N NAOH. ECs at a concentration of 2×10^6 cells/mL and PCs at a concentration of 0.4×10^6 cells/mL were added to the prepared collagen solution and allowed to polymerize in a transwell insert containing a PET membrane with a 0.4 μM pore size (Corning) for one hour. M199 media containing 1:250 dilution of Serum Plus II Medium Supplement (Sigma) with 50 ng ml^{-1}FGF2, 40 ng ml^{-1} SCF, 40 ng ml^{-1} IL-3, and 40 ng ml^{-1} SDF (Peprotech) was added to the receiver plate (500μL) and transwell insert (200μL) and replaced once during 72 hours of culture (8).

Co-Culture Assay: Transwell inserts containing a PET membrane 0.4 μM pores (Corning) were prepared by adding 200 μL of type I collagen prepared as above. Breast organoids PDM92 or PDM250 were processed and added (20,000 cells) to collagen-containing transwells with 60,000 PCs or PC-derived myofibroblasts. The co-cultures were maintained for two weeks in Advanced DMEM/F12 with 1.25 mM N-Acetylcysteine, 5 mM Nicotinamide (Sigma), 1x B27 supplement, 1x Glutamax, 1x HEPES and 100 mg·ml^{-1} Primocin.

Decellularized Matrix: 500,000 cells per well of a six-well plate were cultured for five days. The cultures were decellularized with PBS containing 0.5% Triton X-100 and 20 mM NH_4OH (Sigma-Aldrich) for 5-10 min at 37°C. Matrices were immunostained and imaged or prepared for cell culture. For cell culture, matrices were washed twice with PBS and treated with DNase (10 Kunitz units per ml; Qiagen) for 2 hours at 37°C. As a positive control, additional wells were coated with 400 uL of BME diluted to a 10 mg/mL protein concentration. To the prepared matrices, 25,000 MCF10A cells were added to the growth factor containing MCF-10A medium for three days. The medium was replaced for an additional six days, with the medium omitting supplemental EGF (9).

Conditioned media (CM): CM was generated by culturing 300,000 cells per well of a six-well plate in 1 mL of serum-free basal media for 24 hours. According to the manufacturer's protocols, IL-6, VEGFA, and TGFβ were measured in three biological and technical replicates using ELISA Kits (Raybiotech). Each factor's concentration in the CM was extrapolated from the corresponding standard curve. The manufacturer also analyzed conditioned media using the Quantibody Human Cytokine Array (Raybiotech).

Mouse xenograft experiments: MDA-MB-468 cells (200,000) and BT474 cells (400,000) were injected into the inguinal mammary fat pad of 6–8-week-old NSG mice in a mixture of 50% Matrigel and 50% PBS. This consistently formed a palpable tumor at 21 and 30 days, respectively. The mice were randomly assigned to be treated with Rosiglitazone at a concentration of 10 mg/kg/day or an equivalent volume of vehicle control starting at either day 7 (early intervention) or following palpable tumor formation (treatment). Rosiglitazone was stored at -80°C at a concentration of 5.25 mg/50μL of DMSO. The stock solution was diluted in 10% DMSO, 40% PEG-300, 5% Tween-80, and 45% Saline (0.9%). Rosiglitazone and all components of the aqueous solution were purchased from Sigma-Aldrich. All components were pre-warmed to 37oC to preserve solubility and maintained at that temperature until injection. Rosiglitazone was administered intraperitoneally. In addition, MDA-MB-468 cells (50,000) and BT474 cells (100,000) were injected intraductally into 6-week-old NSG mice using a Hamilton syringe after first cutting the nipple. The cells were injected in 2 μL of PBS containing 0.9% trypan-blue to visualize the solution entering the ductal system. We selected 45 days for our experiment because it allows sufficient time for focal areas of tumor invasion to form without filling up the entire mammary gland. Palpable tumors did not develop during the experimental time course. The mice were randomly assigned to be treated with Rosiglitazone at a concentration of 10 mg/kg/day or an equivalent volume of vehicle control starting on day 14 (early intervention). 250,000 MCFDCIS.com cells were injected subcutaneously into 7- to 10-week-old female NSG mice alone or together with a 3-fold excess (750,000) of myoepithelial cells (MEPs), reduction mammary fibroblasts (RMF), PCs or ECs in 50% Matrigel. The tumors were allowed to grow for three weeks. At the conclusion of the study, the xenograft tumors were weighted, then formalin-fixed and paraffin-embedded by the Histology & Biomarker Core. Animal experiments were conducted following protocols approved by the University of California, San Francisco – Institutional Animal Care Use Committee.

Statistical analysis: The statistical comparisons of the different pairs of data sets were conducted using Welch's t-test, a statistical method to evaluate the differences between two means that might have different variances (GraphPad Prism Software). Levels of significance used were *<0.01, **<0.001, and ***<0.0001. The staining, imaging, and scoring procedures of the Ductal Carcinoma *In Situ* (DCIS) patient cohort were blinded to ensure the impartiality and accuracy of the results. The researchers were unaware of the patient's outcomes during these procedures, minimizing potential bias. An "event" in this context was specifically defined as the occurrence of Invasive Breast Cancer (IBC) on the same side (ipsilateral) where the initial DCIS diagnosis was made. This event had to occur at least six months following the first DCIS diagnosis to qualify under this definition. Cumulative incidence, the probability of occurrence of a particular event over time, was graphed using GraphPad Prism Software. Contingency analysis was performed using Fisher's exact test (GraphPad Prism Software). This test is primarily used for categorical data (in this case, evidence of invasion versus no evidence of invasion in treated versus untreated groups). A logistic regression model was applied to determine the odds ratios (ORs), which provide a measure of association between exposure and an outcome. The logistic regression was performed using the Survival package (version 2.44-1.1) in R (version 3.6.1). Euler diagrams were generated to visualize the overlaps between different data sets. These diagrams were created using the Venneuler package (version 1.1-0) for R.

References:

1. P. Bankhead *et al.*, QuPath: Open source software for digital pathology image analysis. *Sci Rep* **7**, 16878 (2017).
2. E. A. Susaki *et al.*, Advanced CUBIC protocols for whole-brain and whole-body clearing and imaging. *Nat Protoc* **10**, 1709-1727 (2015).
3. C. S. McGinnis *et al.*, MULTI-seq: sample multiplexing for single-cell RNA sequencing using lipid-tagged indices. *Nat Methods* **16**, 619-626 (2019).
4. A. Butler, P. Hoffman, P. Smibert, E. Papalexi, R. Satija, Integrating single-cell transcriptomic data across different conditions, technologies, and species. *Nat Biotechnol* **36**, 411-420 (2018).
5. X. Rao, X. Huang, Z. Zhou, X. Lin, An improvement of the 2^(-delta delta CT) method for quantitative real-time polymerase chain reaction data analysis. *Biostat Bioinforma Biomath* **3**, 71-85 (2013).
6. L. Kou *et al.*, The phenotype and tissue-specific nature of multipotent cells derived from human mature adipocytes. *Biochem Biophys Res Commun* **444**, 543-548 (2014).
7. K. L. DeCicco-Skinner *et al.*, Endothelial cell tube formation assay for the in vitro study of angiogenesis. *J Vis Exp* 10.3791/51312, e51312 (2014).
8. S. S. Kemp, K. N. Aguera, B. Cha, G. E. Davis, Defining Endothelial Cell-Derived Factors That Promote Pericyte Recruitment and Capillary Network Assembly. *Arterioscler Thromb Vasc Biol* **40**, 2632-2648 (2020).
9. J. Debnath, S. K. Muthuswamy, J. S. Brugge, Morphogenesis and oncogenesis of MCF-10A mammary epithelial acini grown in three-dimensional basement membrane cultures. *Methods* **30**, 256-268 (2003).

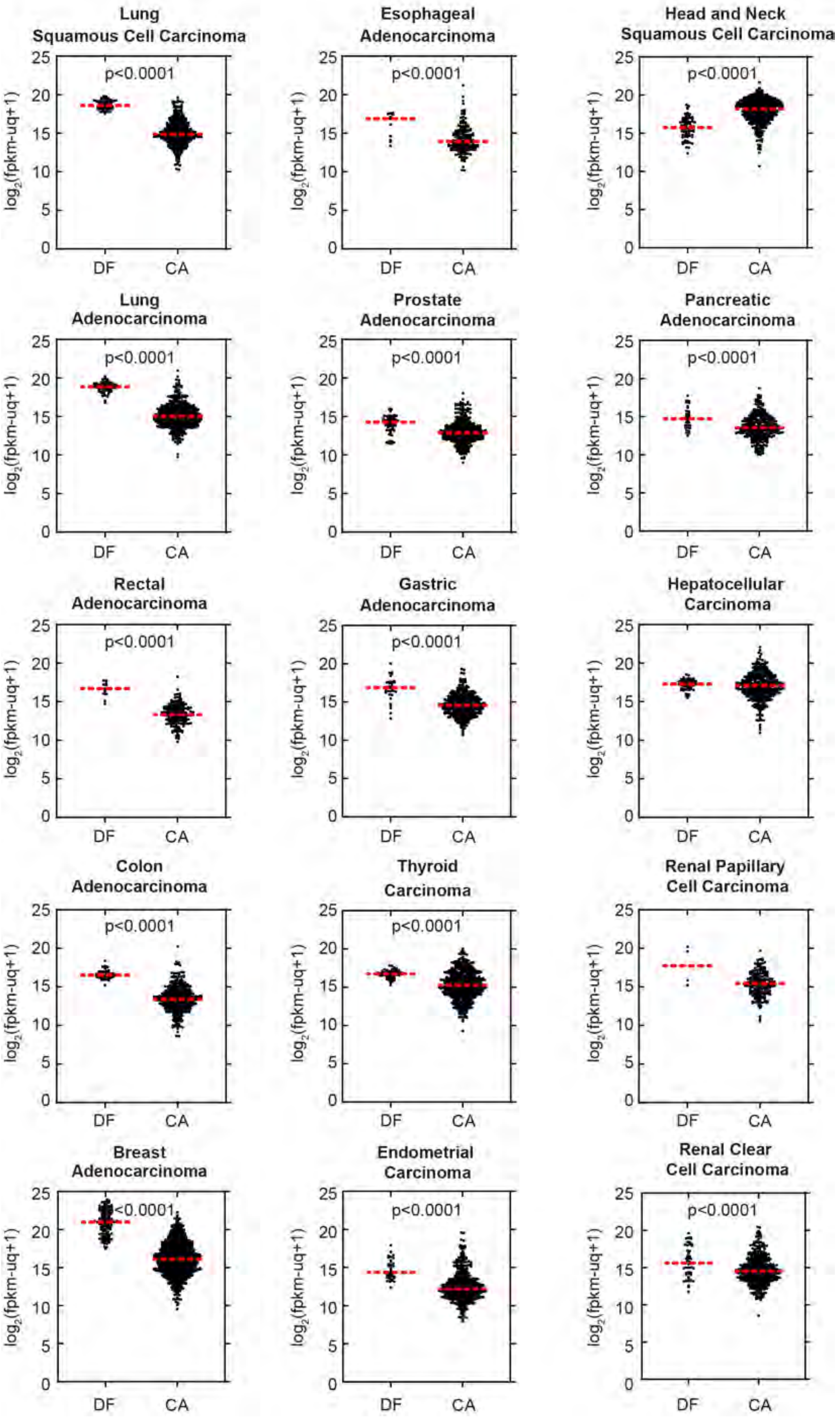

Lung Squamous Cell Carcinoma
p<0.0001
Esophageal Adenocarcinoma
p<0.0001
Head and Neck Squamous Cell Carcinoma
p<0.0001
Lung Adenocarcinoma
p<0.0001
Prostate Adenocarcinoma
p<0.0001
Pancreatic Adenocarcinoma
p<0.0001
Rectal Adenocarcinoma
p<0.0001
Gastric Adenocarcinoma
p<0.0001
Hepatocellular Carcinoma
Colon Adenocarcinoma
p<0.0001
Thyroid Carcinoma
p<0.0001
Renal Papillary Cell Carcinoma
Breast Adenocarcinoma
<0.0001
Endometrial Carcinoma
p<0.0001
Renal Clear Cell Carcinoma
p<0.0001
log2(fpkm-uq+1)
0
5
10
15
20
25
DF
CA

Supplemental Figure 1: Tumor tissues exhibited lower levels of CD36 expression compared to disease-free tissues in TCGA RNA-Seq-based transcriptome data. Normalized mRNA expression levels were available for 15 tumor types in the TCGA database (downloaded from: https://portal.gdc.cancer.gov). For each tumor type, normalized CD36 expression levels were plotted for disease-free (DF) and cancer (CA) tissues. The red dotted line indicates the mean. Welch's t-test determined statistical significance. Although the number of disease-free samples was low for several of the tissues analyzed (Renal Papillary Cell Carcinoma), significantly lower levels of CD36 were evident in most cancer types, including breast, colorectal, lung, and esophagus, when compared to their disease-free counterparts. A significant increase in CD36 levels was evident only in Head and Neck Squamous Cell Carcinoma compared to disease-free tissue. No difference was observed in CD36 levels when Hepatocellular Carcinoma was compared to disease-free tissue.

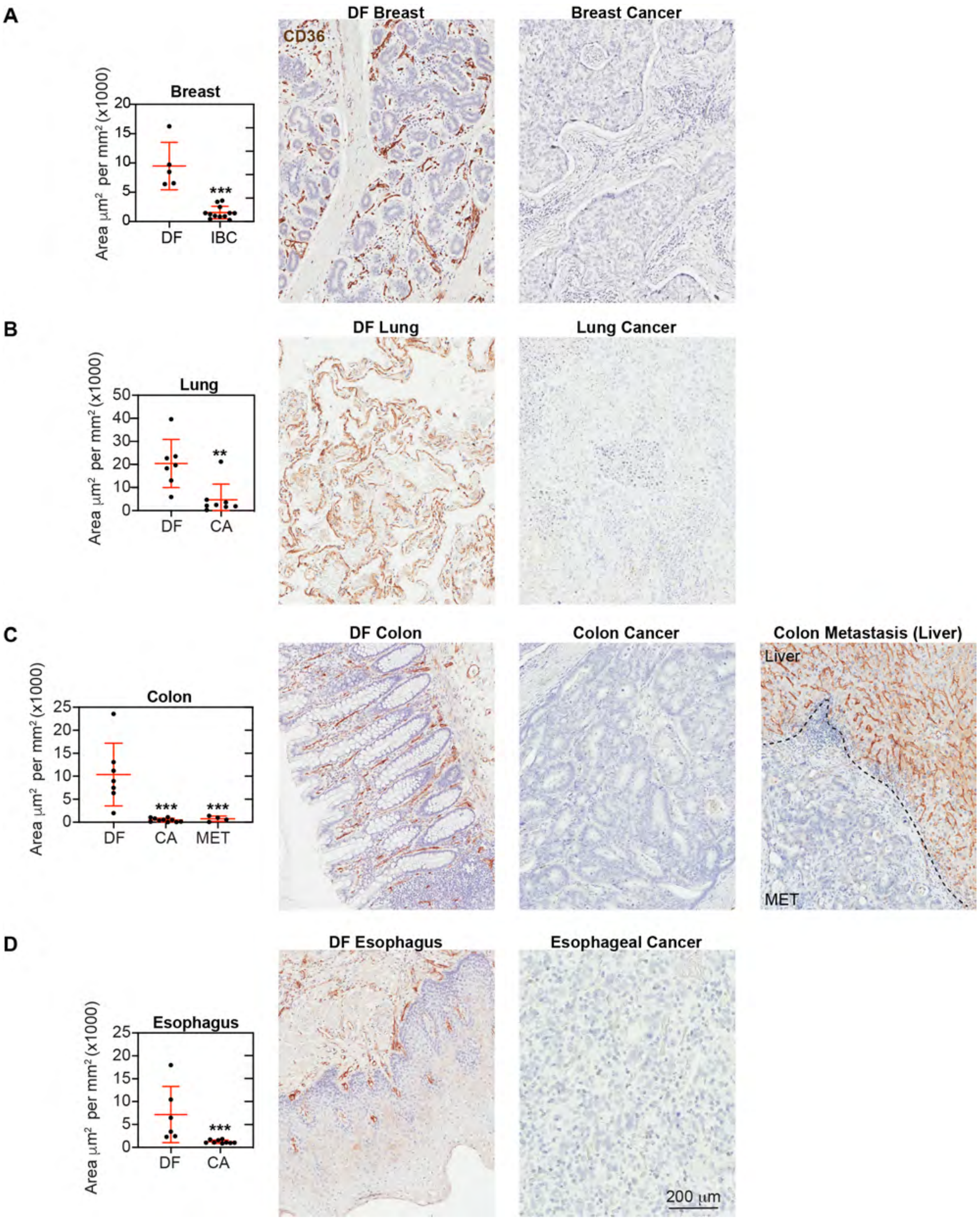
A
CD36
DF Breast
Breast Cancer
Breast
Area µm² per mm² (x1000)
DF
IBC
***
B
DF Lung
Lung Cancer
Lung
DF
CA
**
C
DF Colon
Colon Cancer
Colon Metastasis (Liver)
Liver
MET
Colon
DF
CA
MET
***
D
DF Esophagus
Esophageal Cancer
Esophagus
DF
CA
***
200 µm

Supplemental Figure 2: Reduced CD36 immunoreactivity observed in the stroma surrounding breast, colorectal, lung, and esophageal tumors compared to their disease-free counterparts. FFPE sections of disease-free and tumor tissues were probed with an antibody against CD36 (DAB, brown), counterstained with hematoxylin (light blue), and scanned (20x). The CD36-positive area (exceeding the positive signal threshold, set using antibody omitted controls) was quantified and normalized to the total area analyzed for each specimen. (A) Disease-free (DF) breast (n=7) and invasive breast cancer (IBC) (n=7). (B) Disease-free colon (n=7), colorectal cancer (n=10), and colorectal cancer metastases to the liver (n=4). (C) Disease-free lung (n=6) and lung adenocarcinoma (n=6). (D) Disease-free esophagus (n=6) and esophageal adenocarcinoma (n=9). The mean and standard deviation are shown in red. Welch's t-test determined significance. Cancer samples demonstrated lower CD36 expression in the selected tissues compared to disease-free samples. Colon metastasis to the liver demonstrated a particularly striking loss of CD36 compared to the robust expression of CD36 in the liver sinusoidal ECs.

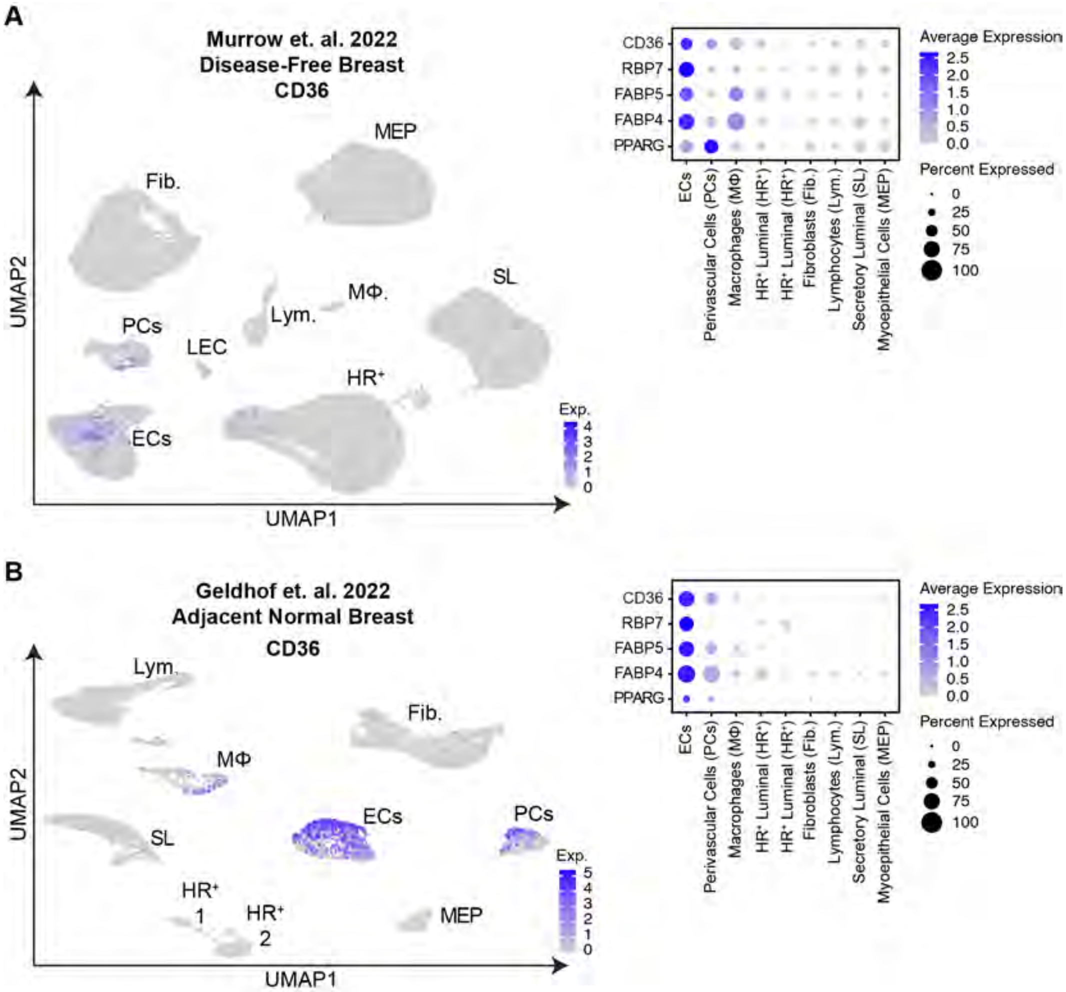
A
Murrow et. al. 2022
Disease-Free Breast
CD36
MEP
Fib.
SL
MΦ.
Lym.
PCs
LEC
HR+
ECs
UMAP2
UMAP1
Exp.
CD36
RBP7
FABP5
FABP4
PPARG
ECs
Perivascular Cells (PCs)
Macrophages (MΦ)
HR+ Luminal (HR+)
HR+ Luminal (HR+)
Fibroblasts (Fib.)
Lymphocytes (Lym.)
Secretory Luminal (SL)
Myoepithelial Cells (MEP)
Average Expression
Percent Expressed
B
Geldhof et. al. 2022
Adjacent Normal Breast
CD36
Lym.
Fib.
MΦ
ECs
PCs
SL
HR+ 1
HR+ 2
MEP
UMAP2
UMAP1
Exp.

Supplemental Figure 3: Enrichment of CD36 transcript in disease-free breast endothelial cells (ECs), pericytes (PCs), and macrophages. (A) Excluding adipocytes lost during tissue processing, the full range of cell types in human breast tissue specimens (from reduction mammoplasty) was analyzed using scRNA-seq and published in Murrow et al. 2022. CD36 expression is shown on a per-cell basis in the UMAP projection. In addition, the averaged expression of CD36, PPARγ, FABP4, FABP5, and RBP7 are shown for each cell type identified in the dot plot. (B) Geldhof et al. 2022 presented scRNA-seq data of histologically normal breast tissue adjacent to breast cancer. Their data was downloaded from the gene expression omnibus database: accession code GSE155109. Only cells isolated from normal adjacent tissue were analyzed. CD36 and several other PPARγ target genes were primarily observed in ECs, perivascular cells, and macrophages in these cohorts.

Disease-Free Breast

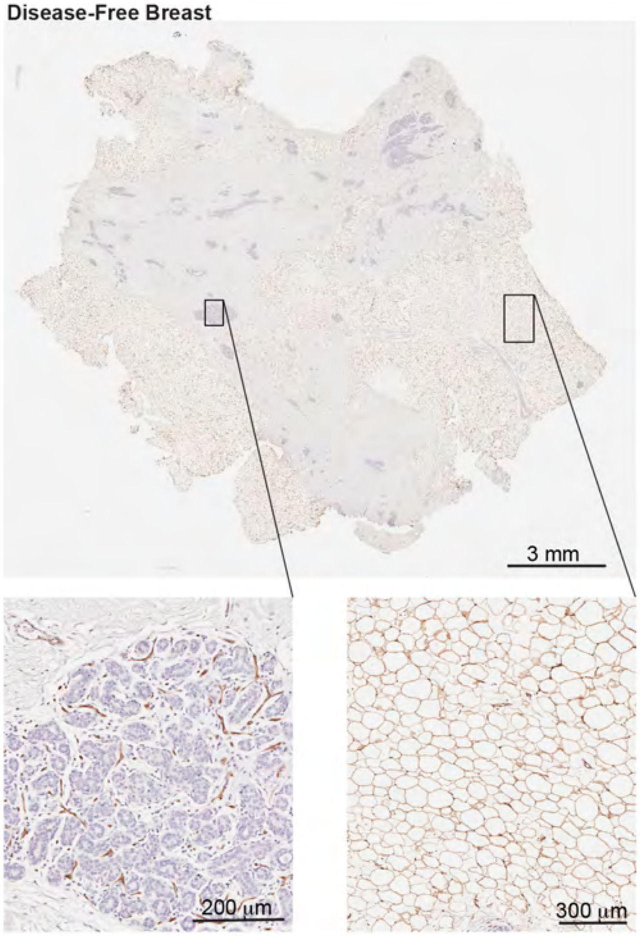

Invasive Breast Cancer

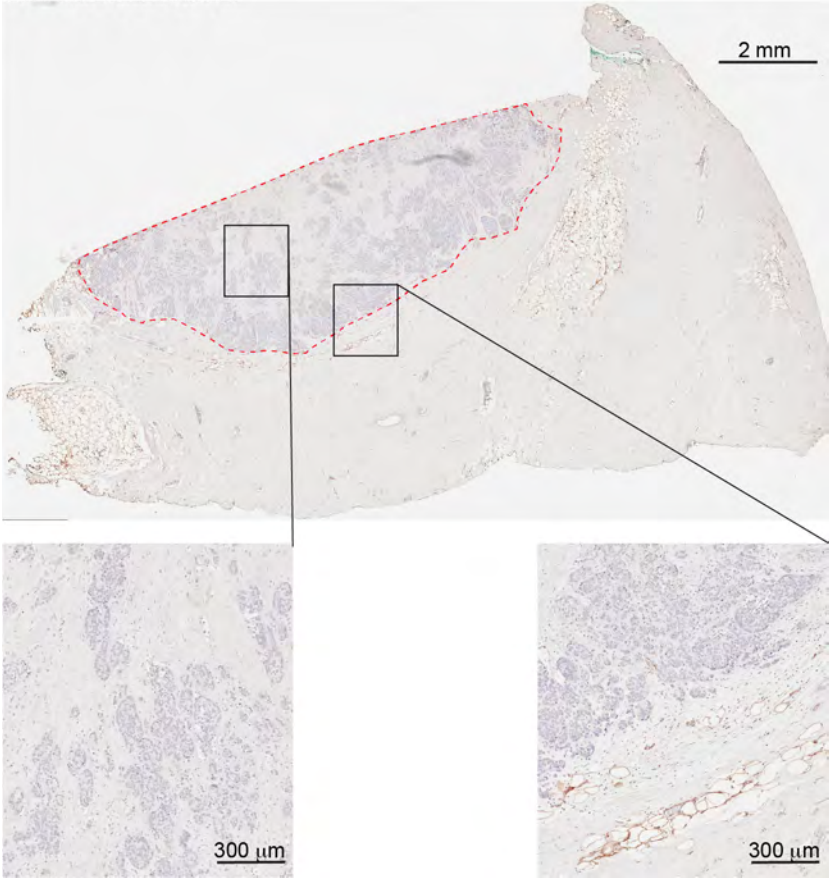

Supplemental Figure 4: **CD36 immunoreactive adipocytes are abundant in disease-free breasts but lacking in the tumor microenvironment.** FFPE sections of disease-free breast and invasive breast cancer (IBC) were probed with an antibody against CD36 (DAB, brown), counterstained with hematoxylin (light blue), and scanned (20x). CD36 immunopositive adipocytes are abundantly present in the disease-free breast intermixed with a collagenous extracellular matrix and disease-free epithelium; however, adipocytes are primarily absent from the stromal microenvironment directly adjacent to breast tumor cells.

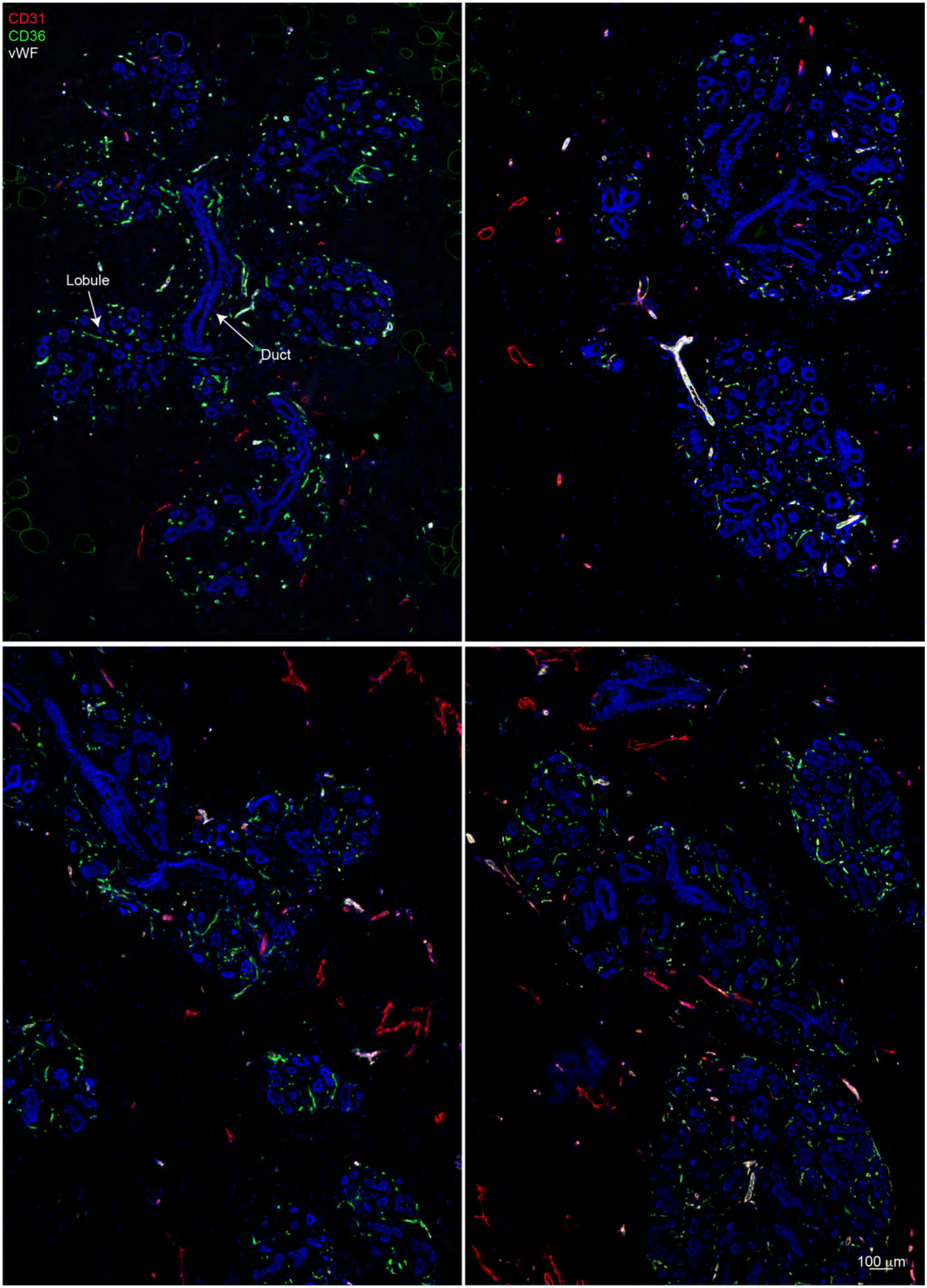
CD31
CD36
vWF
Lobule
Duct
100 μm

Supplemental Figure 5: **CD36 immunoreactive capillaries concentrated in the stroma surrounding disease-free terminal ductal lobular units.** FFPE sections of disease-free breast tissue from four donors were subjected to mIHC analysis using antibodies against vWF, CD36, and CD31. In all cases examined, $CD36^+$ vessels were concentrated proximal to the lobule and/or terminal duct; however, their abundance varies between tissues. Using this mIHC technique, these $CD36^+$ vessels were inconsistently immunopositive for CD31 and/or vWF, but the extent of overlap varies between tissue donors. Larger vessels predominately expressing CD31 and/or vWF tended to lack CD36 expression and were primarily located in the interlobular stroma. Lymphatic vessels in the interlobular stroma also express CD31.

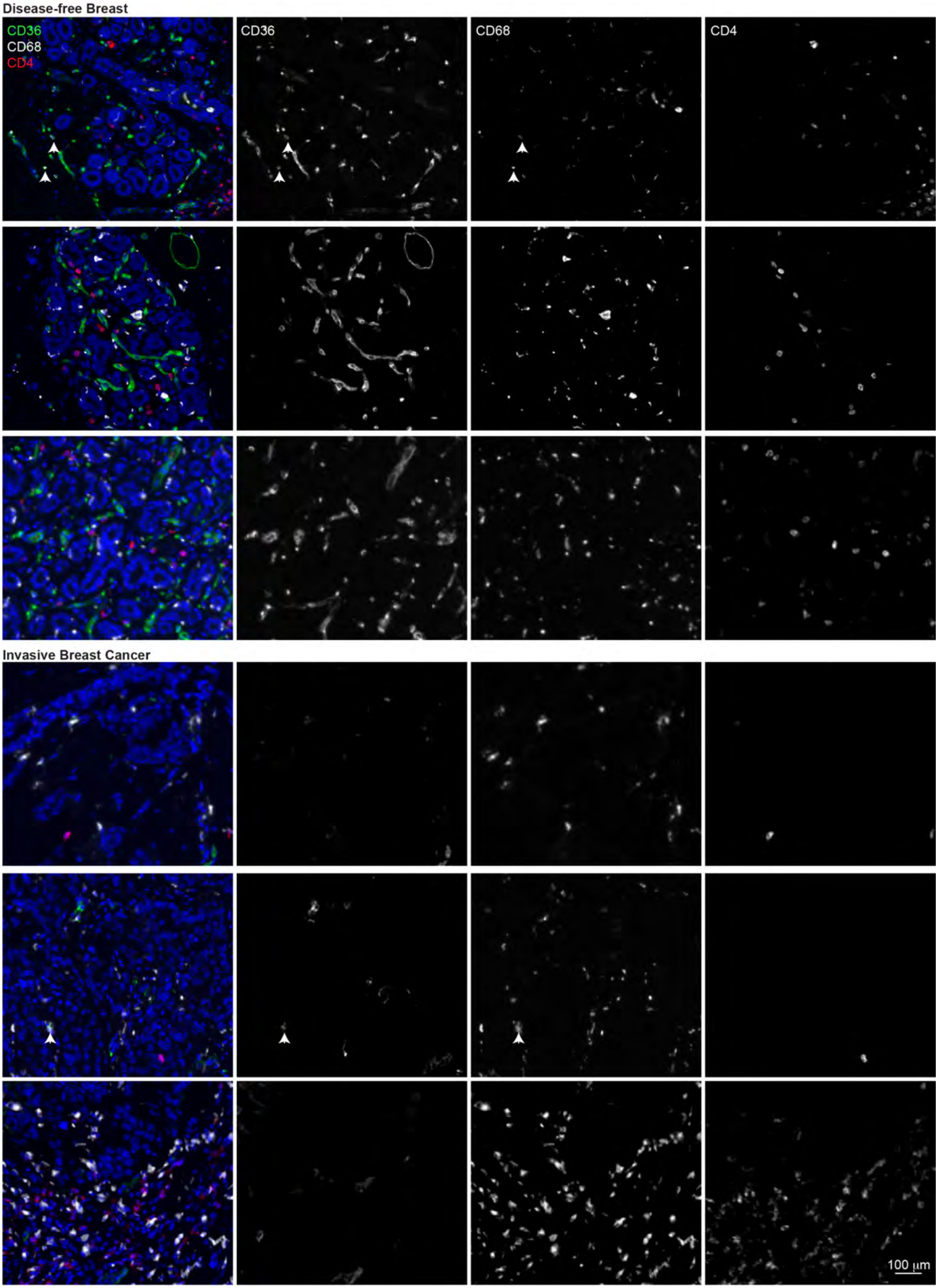
Disease-free Breast
CD36
CD68
CD4
CD36
CD68
CD4
Invasive Breast Cancer
100 μm

Supplemental Figure 6: CD36 immunoreactivity observed in rare CD68$^+$ macrophages but not CD4$^+$ T-cells. FFPE sections of three disease-free breast tissues and three invasive breast cancer tissues were subjected to mIHC analysis using antibodies against CD36, CD68, and CD4 and imaged with a 20x objective. Overall, CD68$^+$ macrophages were rarely immunopositive for CD36 using this mIHC technique but can be observed in some tissues (white arrows). Given their rarity, meaningful quantification would be difficult.

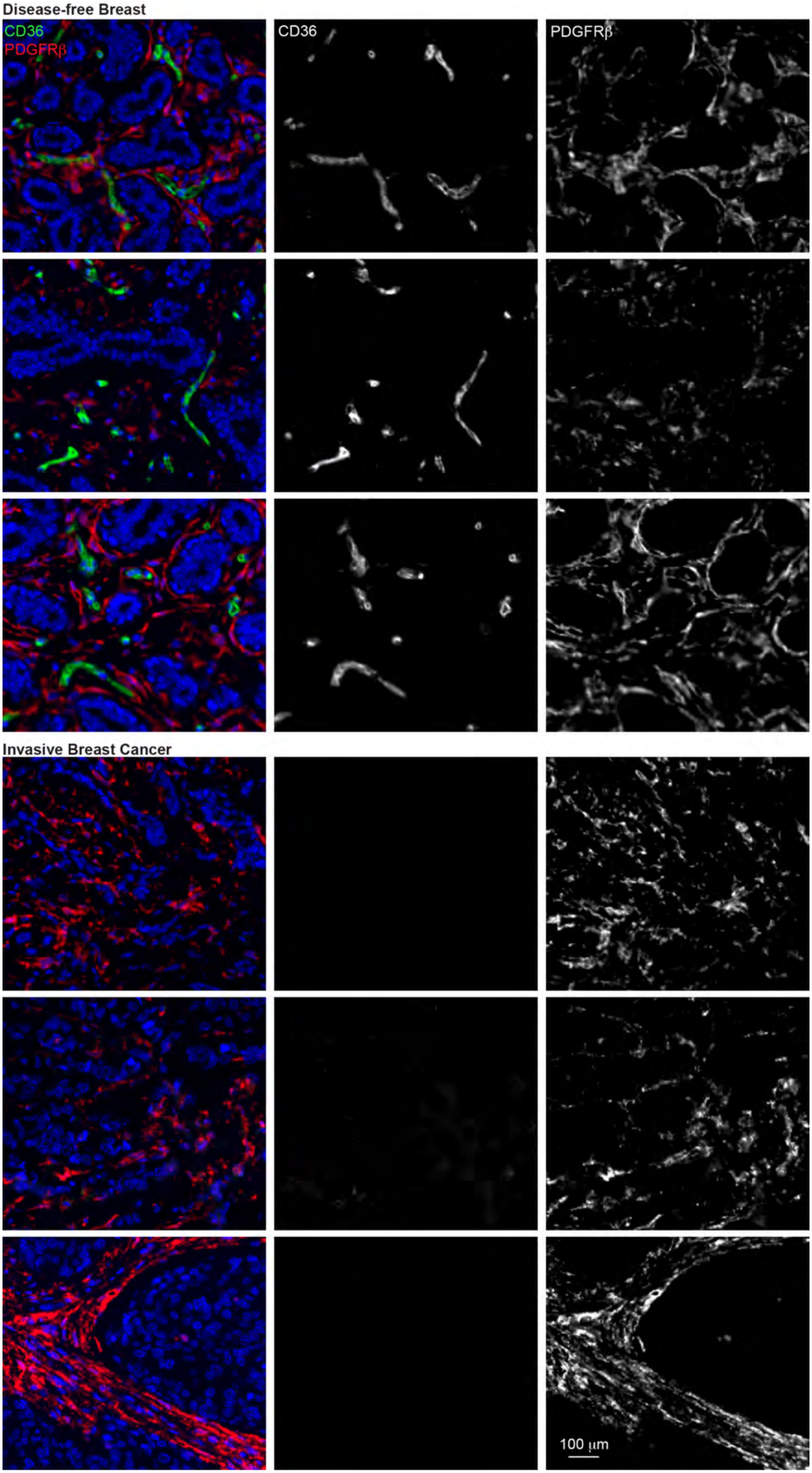
Disease-free Breast
CD36
PDGFRβ
CD36
PDGFRβ
Invasive Breast Cancer
100 μm

Supplemental Figure 7: CD36 immunoreactivity absent from PDGFRβ$^+$ fibroblasts. FFPE sections of three disease-free breast tissues and three invasive breast cancer tissues were subjected to mIHC analysis using antibodies against CD36 and PDGFRβ and imaged with a 20x objective. Fibroblasts surrounding the disease-free breast epithelium robustly express PDGFRβ but lack CD36. PDGFRβ fibroblasts in IBC samples were CD36 negative. Notably, pericytes also express PDGFRβ; however, these cells were difficult to visualize in thin FFPE sections due to their small cell bodies and thin processes wrapping around capillaries.

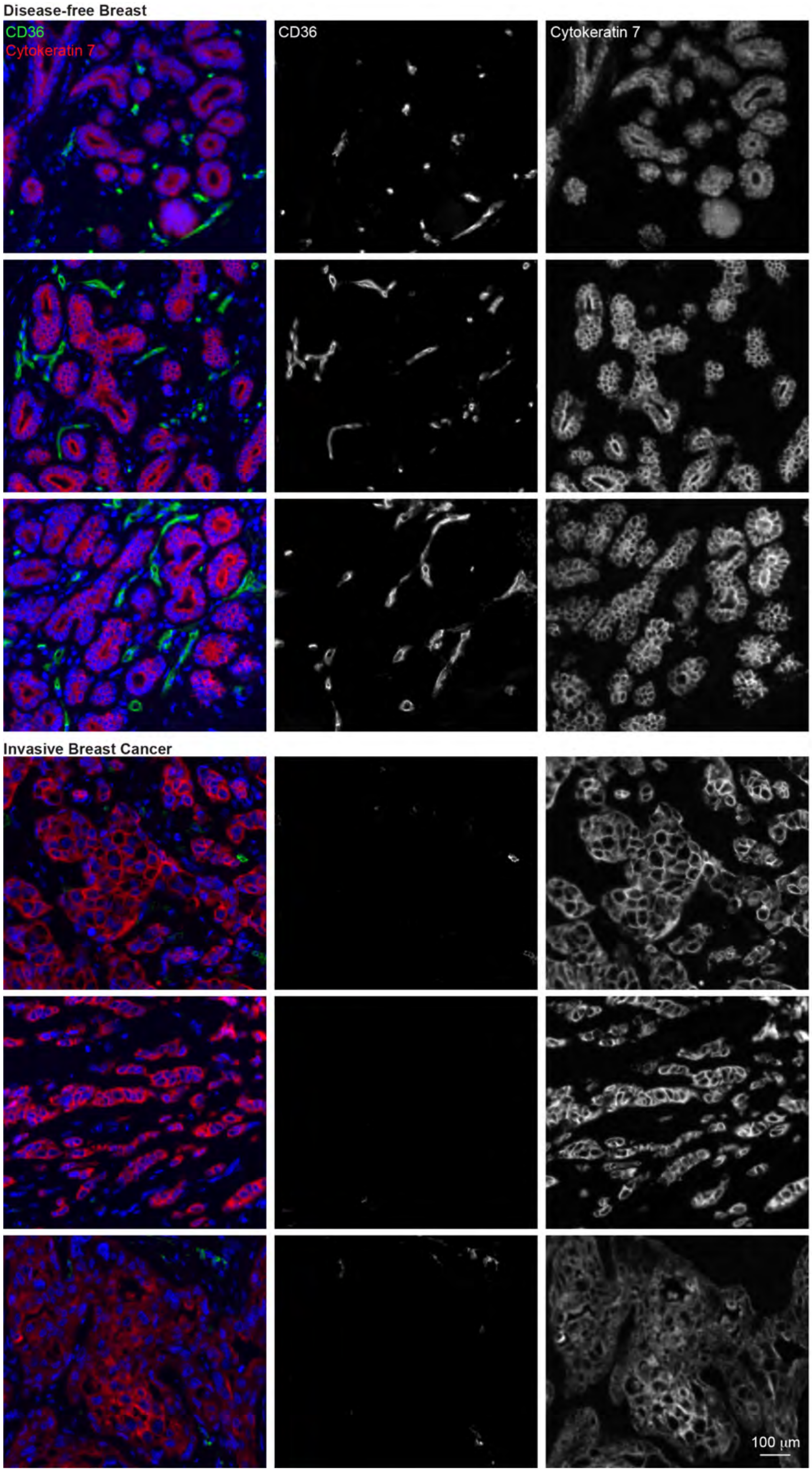
Disease-free Breast
CD36
Cytokeratin 7
CD36
Cytokeratin 7
Invasive Breast Cancer
100 μm

Supplemental Figure 8: **CD36 immunoreactivity absent from *cytokeratin 7^{+} epithelial cells.*** FFPE sections of three disease-free breast tissues and three invasive breast cancer tissues were subjected to mIHC analysis using antibodies against CD36 and Cytokeratin 7 and imaged with a 20x objective. In resting disease-free breast tissue and IBC specimens, we did not observe co-localization of CD36 and the primarily luminal cytokeratin 7.

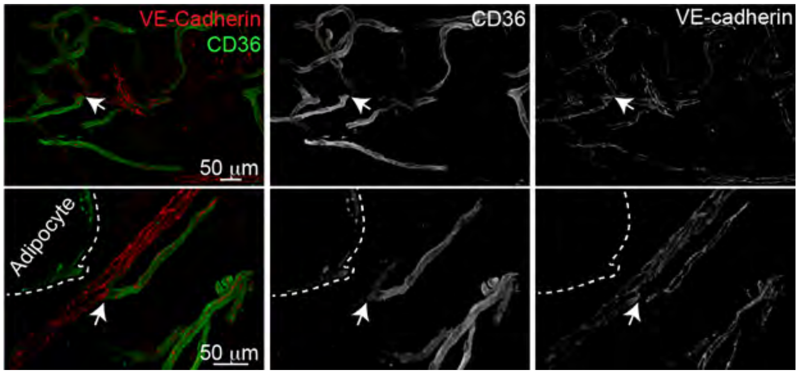

VE-Cadherin
CD36
50 μm
CD36
VE-cadherin
Adipocyte
50 μm

Supplemental Figure 9. Confocal imaging visualizes VE-cadherin positive endothelial adherens junctions within the intralobular capillary network. Tissue sections (200 μM thick) from (RM317; top row) and (RM318; bottom row) were immunostained for CD36 and VE-cadherin (50-100 μm Z-stacks). The white arrows indicate branch points between larger capacity components of the breast microvasculature (i.e., arterioles or venules) and intralobular capillaries.

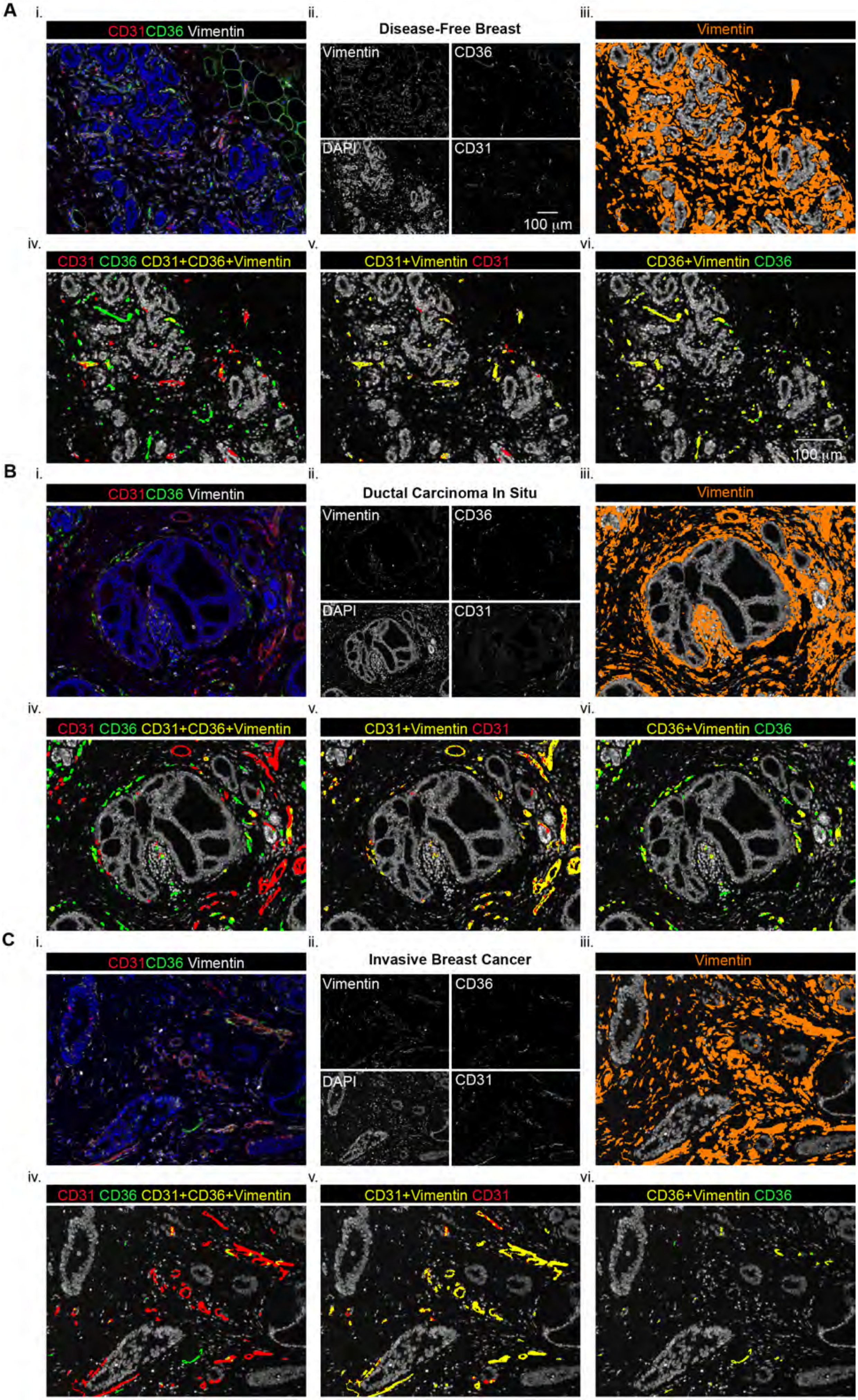

A
i.
CD31CD36 Vimentin
ii.
Disease-Free Breast
Vimentin
CD36
DAPI
CD31
100 μm
iii.
Vimentin
iv.
CD31 CD36 CD31+CD36+Vimentin
v.
CD31+Vimentin CD31
vi.
CD36+Vimentin CD36
100 μm
B
i.
CD31CD36 Vimentin
ii.
Ductal Carcinoma In Situ
Vimentin
CD36
DAPI
CD31
iii.
Vimentin
iv.
CD31 CD36 CD31+CD36+Vimentin
v.
CD31+Vimentin CD31
vi.
CD36+Vimentin CD36
C
i.
CD31CD36 Vimentin
ii.
Invasive Breast Cancer
Vimentin
CD36
DAPI
CD31
iii.
Vimentin
iv.
CD31 CD36 CD31+CD36+Vimentin
v.
CD31+Vimentin CD31
vi.
CD36+Vimentin CD36

***Supplemental Figure 10. Scoring methodology for CD36 and CD31 mIHC images*.** FFPE sections of (A) Disease-free breast, (B) DCIS, and (C) IBC were subjected to mIHC with antibodies against CD36, CD31, and vimentin. (A, C) (i) Pseudo-colored composite image of CD36 (green), CD31(red), vimentin (white), and DAPI (blue) following spectral deconvolution. (ii) Monochromatic images of CD36, CD31, vimentin, and DAPI signals. (iii) Threshold-based scoring of the non-immune stroma based on vimentin signal. (iv) Threshold-based scoring showing CD36 signal alone (green), CD31 signal alone (red), and colocalization of CD36, CD31, and vimentin signal on a monochromatic image of DAPI. (v) same as in iv, showing CD31 signal alone (red) and colocalization of CD31 and vimentin signal (yellow). (vi) same as in iv, showing CD36 signal alone (green) and colocalization of CD36 and vimentin signal (yellow).

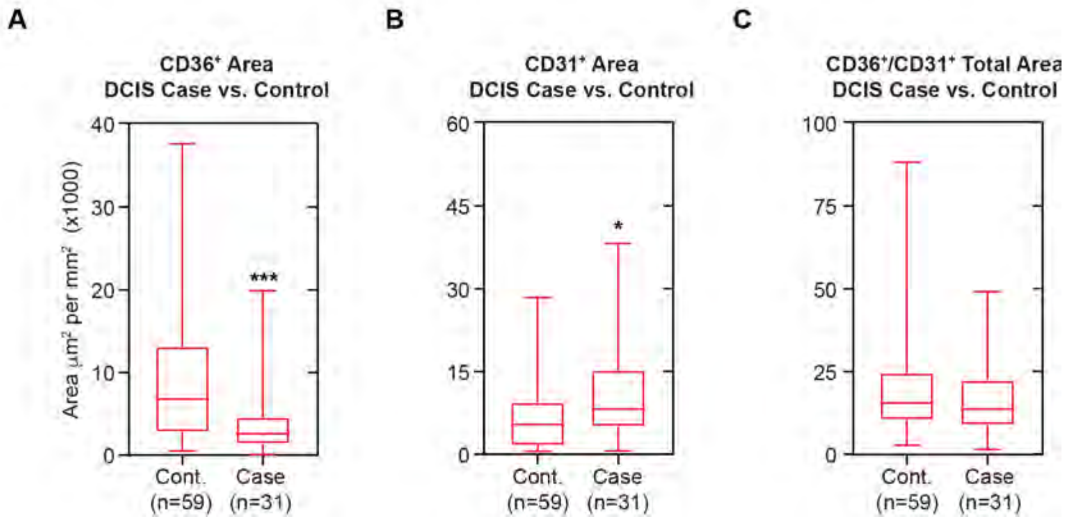
A
CD36+ Area
DCIS Case vs. Control
Area µm² per mm² (x1000)
40
30
20
10
0
***
Cont.
(n=59)
Case
(n=31)
B
CD31+ Area
DCIS Case vs. Control
60
45
30
15
0
*
Cont.
(n=59)
Case
(n=31)
C
CD36+/CD31+ Total Area
DCIS Case vs. Control
100
75
50
25
0
Cont.
(n=59)
Case
(n=31)

Supplemental Figure 11. A similar total vascular area ($CD36^+$ and/or $CD31^+$) was observed in DCIS cases and controls. (A) Total area scoring positive for CD36 in DCIS controls vs. cases. (B) Total area scoring positive for CD31 expression in DCIS controls vs. cases. (C) Total area scoring positive for CD36 and/or CD31 combined for DCIS controls vs. DCIS cases. This figure shows that the vascular staining area (μm^2) for CD36 normalized to the total area analyzed (mm^2) was significantly lower in DCIS lesions of cases compared to controls. In contrast, the CD31 area in DCIS lesions of cases was significantly higher compared to controls. Therefore, the total vascular area measured (CD36 + CD31) was not significantly different between DCIS cases and controls. The boxplot shows the median and range of the data. Welch's t-test determined significance.

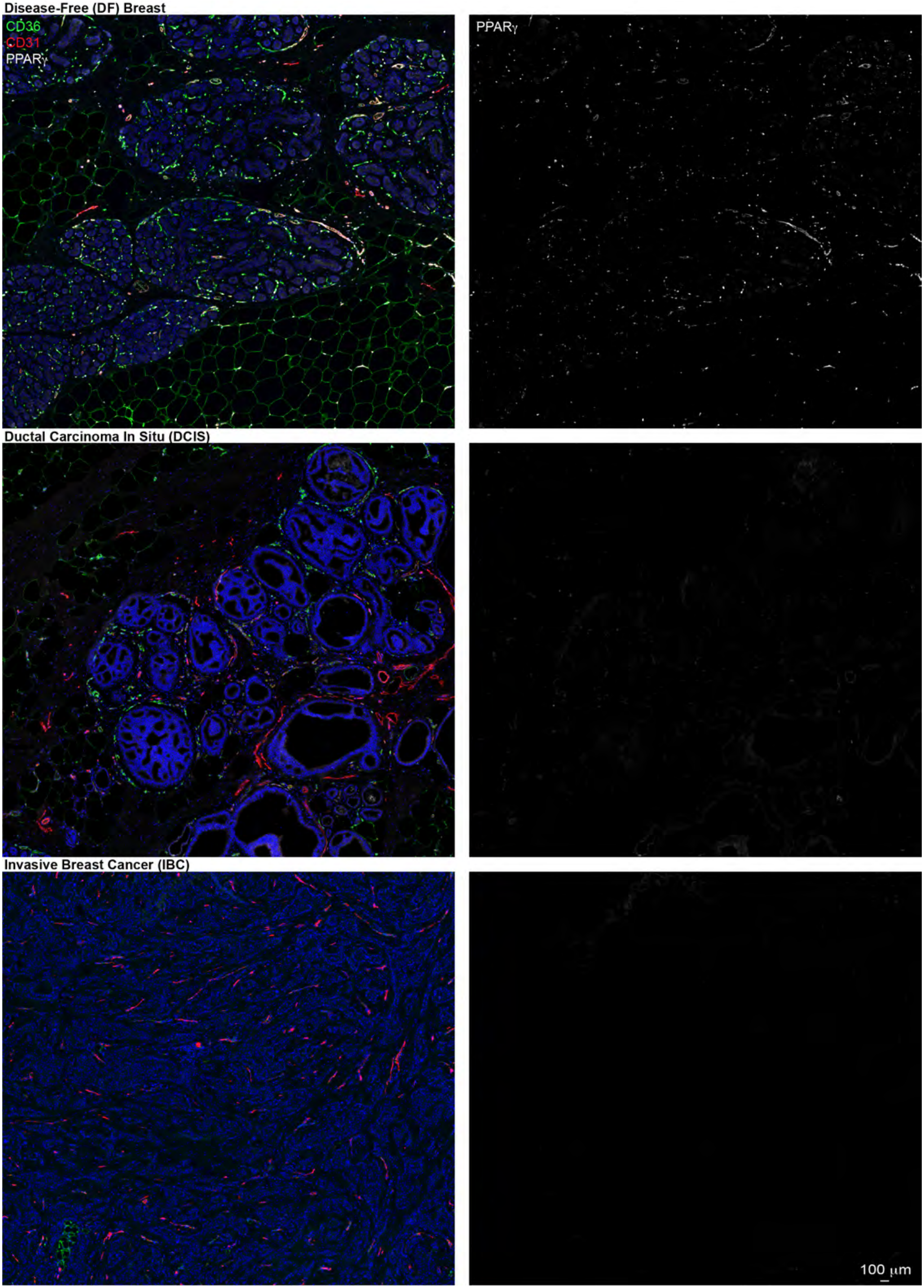

Disease-Free (DF) Breast
CD36
CD31
PPARγ
PPARγ
Ductal Carcinoma In Situ (DCIS)
Invasive Breast Cancer (IBC)
100 μm

Supplemental Figure 12*: *PPARγ immunoreactivity lost during breast tumor progression. FFPE sections of disease-free breast, DCIS, and IBC tissue were subjected to mIHC for PPARγ (white), CD36 (green), and CD31 (red) and counterstained with DAPI (blue). A significant reduction in PPARγ expression can be observed in the vasculature and proximal adipocytes surrounding DCIS and IBC compared to the disease-free breast. PPARγ immunopositivity exhibited a nuclear localization in most adipocytes and CD36-expressing capillaries. Notably, CD31-expressing vessels in some disease-free specimens exhibited cytoplasmic staining of PPARγ. The high level of PPARγ immunopositivity in disease-free breast tissue starkly contrasted with the low levels detected in DCIS and a near complete lack of PPARγ in IBC tissues.

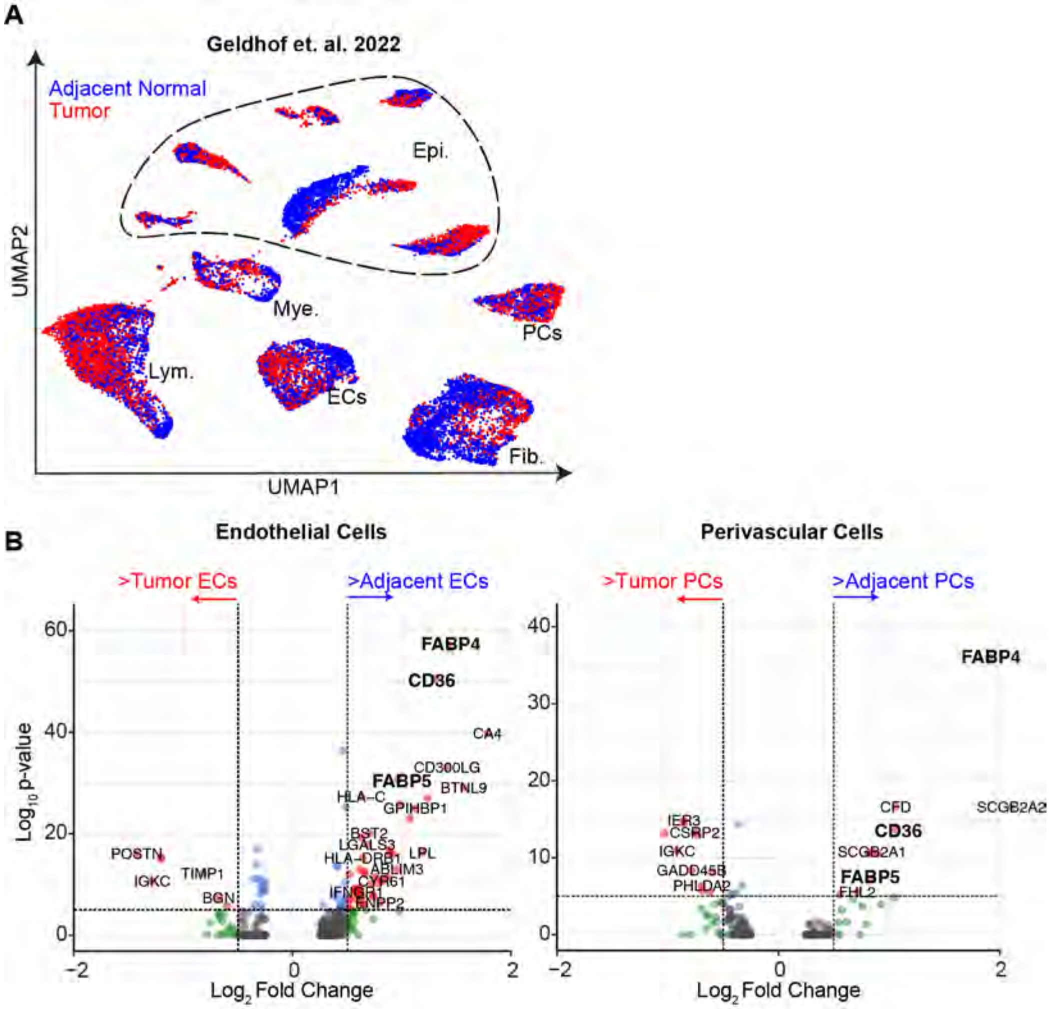
A
Geldhof et. al. 2022
Adjacent Normal
Tumor
Epi.
Mye.
PCs
Lym.
ECs
Fib.
UMAP2
UMAP1
B
Endothelial Cells
>Tumor ECs
>Adjacent ECs
FABP4
CD36
CA4
CD300LG
FABP5
BTNL9
HLA-C
GPIHBP1
BST2
LGALS3
LPL
HLA-DRB1
ABLIM3
POSTN
IGKC
TIMP1
CYR61
BGN
Log2 Fold Change
Log10 p-value
Perivascular Cells
>Tumor PCs
>Adjacent PCs
FABP4
IER3
CSRP2
CFD
SCGB2A2
CD36
IGKC
SCGB2A1
GADD45B
FABP5
PHLDA2
FHL2
Log2 Fold Change

Supplemental Figure 13. Lower transcript levels of PPARγ target genes were observed in both tumor-associated ECs and PCs compared to their disease-free counterparts. (A) Geldhof et al. 2022 presented scRNA-seq data of stroma cell data from both breast tumors and adjacent normal breast tissue. The data was downloaded from the gene expression omnibus database: accession code GSE155109. PPARγ-target genes were among the most downregulated genes in tumor ECs, as discussed in their paper. (B) In their dataset, we analyzed differential gene expression in both endothelial cells and PCs. In addition to ECs, PPARγ-target genes (e.g., FABP4, FABP5, and CD36) were also among the most downregulated by PCs within the tumor region compared to the normal adjacent region.

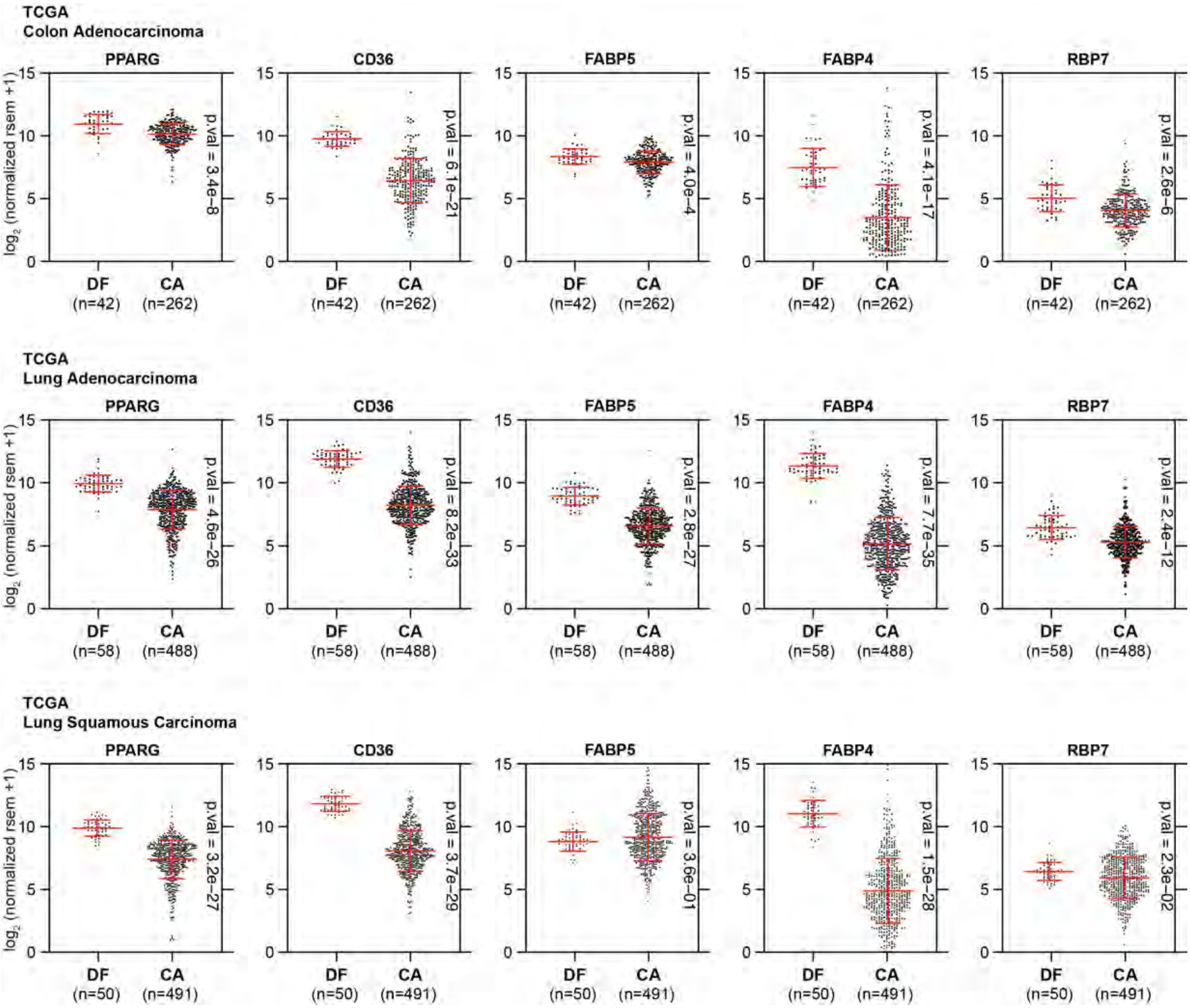

TCGA
Colon Adenocarcinoma
PPARG
CD36
FABP5
FABP4
RBP7
log2 (normalized rsem +1)
p.val = 3.4e−8
p.val = 6.1e−21
p.val = 4.0e−4
p.val = 4.1e−17
p.val = 2.6e−6
DF (n=42)
CA (n=262)
TCGA
Lung Adenocarcinoma
PPARG
CD36
FABP5
FABP4
RBP7
log2 (normalized rsem +1)
p.val = 4.6e−26
p.val = 8.2e−33
p.val = 2.8e−27
p.val = 7.7e−35
p.val = 2.4e−12
DF (n=58)
CA (n=488)
TCGA
Lung Squamous Carcinoma
PPARG
CD36
FABP5
FABP4
RBP7
log2 (normalized rsem +1)
p.val = 3.2e−27
p.val = 3.7e−29
p.val = 3.6e−01
p.val = 1.5e−28
p.val = 2.3e−02
DF (n=50)
CA (n=491)

Supplemental Figure 14. Colon and lung cancer tissues exhibit lower levels of PPARγ target genes compared to disease-free tissues in TCGA RNA-Seq-based transcriptome data. Gene expression profiles for PPARG, CD36, FABP4, FABP5, and RBP7 in disease-free and cancer tissues by RNA-Seq from The Cancer Genome Atlas (TCGA) presented as $\log_2$ RSEM (RNA-Seq by Expectation-Maximization) +1. The mean and standard deviation are shown in red. The Wilcoxon rank-sum test calculated significance. Data for colon adenocarcinoma, lung adenocarcinoma, and lung squamous carcinoma are shown.

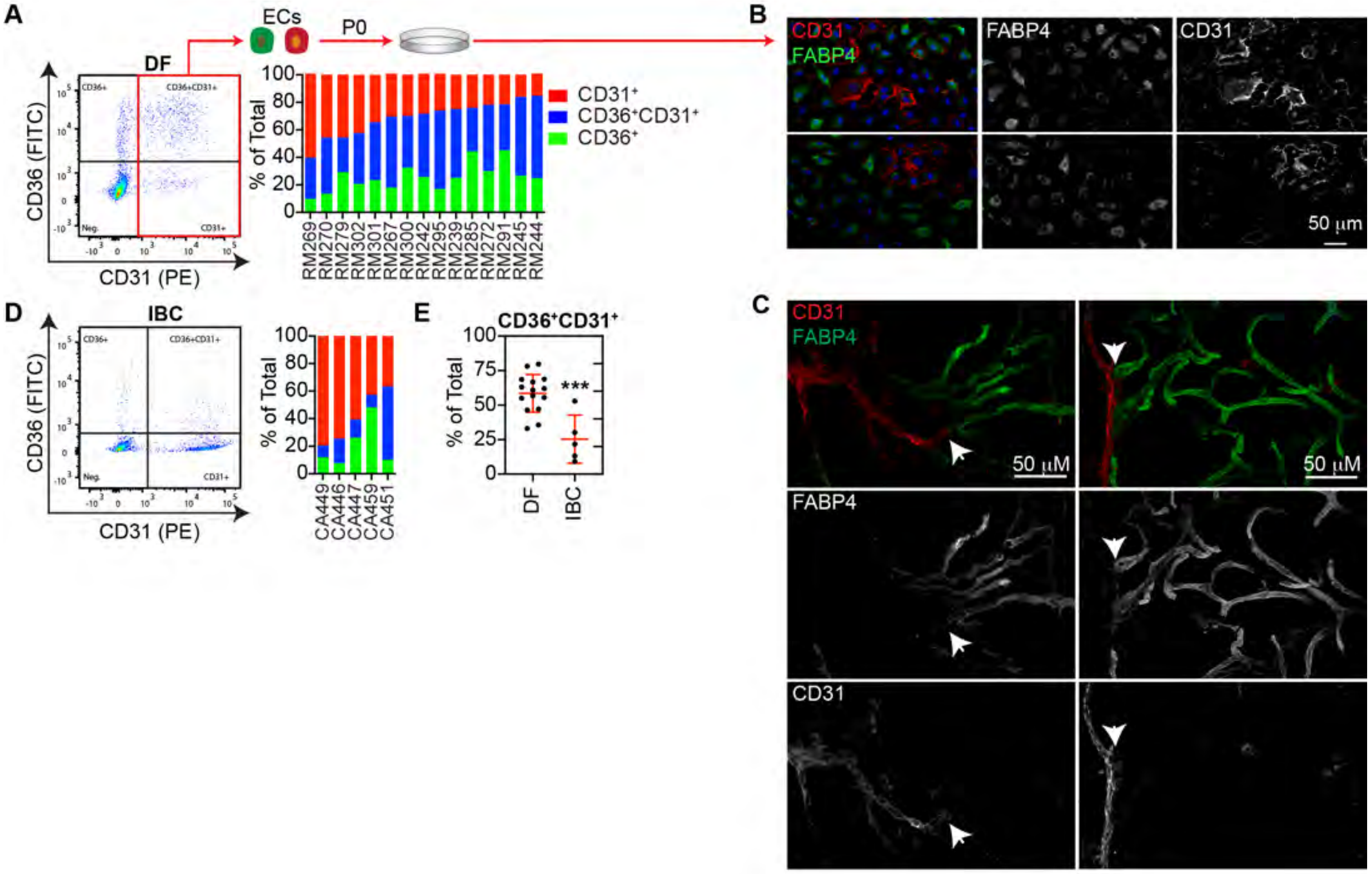
A
B
C
D
E
ECs
P0
DF
CD36+
CD36+CD31+
Neg.
CD31+
CD36 (FITC)
CD31 (PE)
100
80
60
40
20
0
% of Total
CD31⁺
CD36⁺CD31⁺
CD36⁺
RM269
RM270
RM279
RM302
RM301
RM267
RM300
RM242
RM295
RM239
RM285
RM272
RM291
RM245
RM244
CD31
FABP4
50 µm
50 µM
IBC
CA449
CA446
CA447
CA459
CA451
CD36⁺CD31⁺
75
50
25
***
DF
IBC

Supplemental Figure 15: Flow cytometry analyzes CD36 and CD31 expression in disease-free breast and invasive breast cancer (IBC). (A) Human breast tissues from reduction mammoplasties were processed into single cells. Doublets, dead, and immune cells were excluded. For the remaining cells, the expression levels of CD31 and CD36 were displayed on a per-cell basis in the scatter plots. The proportion of each EC phenotype: (1) $CD31^{-}CD31^{+}$ [red], (2) $CD36^{+}CD31^{-}$ [green], and (3) both $CD31^{+}CD36^{+}$ [blue] are displayed as a proportion of all three groups combined (1+2+3). (B) ECs isolated by FACS based on the expression of CD31 were immunostained for CD31 (red) and FABP4 (green), counterstained with DAPI (blue). Due to the high sensitivity of flow cytometry-based methods, we detected more $CD36^{+}CD31^{+}$ ECs in disease-free breast tissues than expected based on our mIHC analysis (Figure 3A). Immunostaining demonstrates a high degree of heterogeneity in CD31 expression in early passage ECs. Notice that the ECs with the highest levels of FABP4 tend to express lower levels of CD31 and vice versa. We chose to stain FABP4 instead of CD36 because it is cytoplasmic and easy to interpret when paired with membrane-associated CD31. (C) Tissue (sectioned to 200 μM thick) from a 21-years-old nulliparous woman (left) and a 39-year-old parous woman (right) were immunostained for FABP4 and CD31 (50-100 μm Z-stacks). The white arrows indicate branch points between larger capacity components of the breast microvasculature (i.e., arterioles or venules) and intralobular capillaries. (D) The same analysis as in A was performed on human breast tumor tissue. (E) The percentage of $CD31^{+}$ ECs that expressed CD36 was compared between disease-free breast and tumor tissue. Similar to our mIHC analysis, flow cytometry demonstrates a loss of CD36 expression in the tumor vasculature.

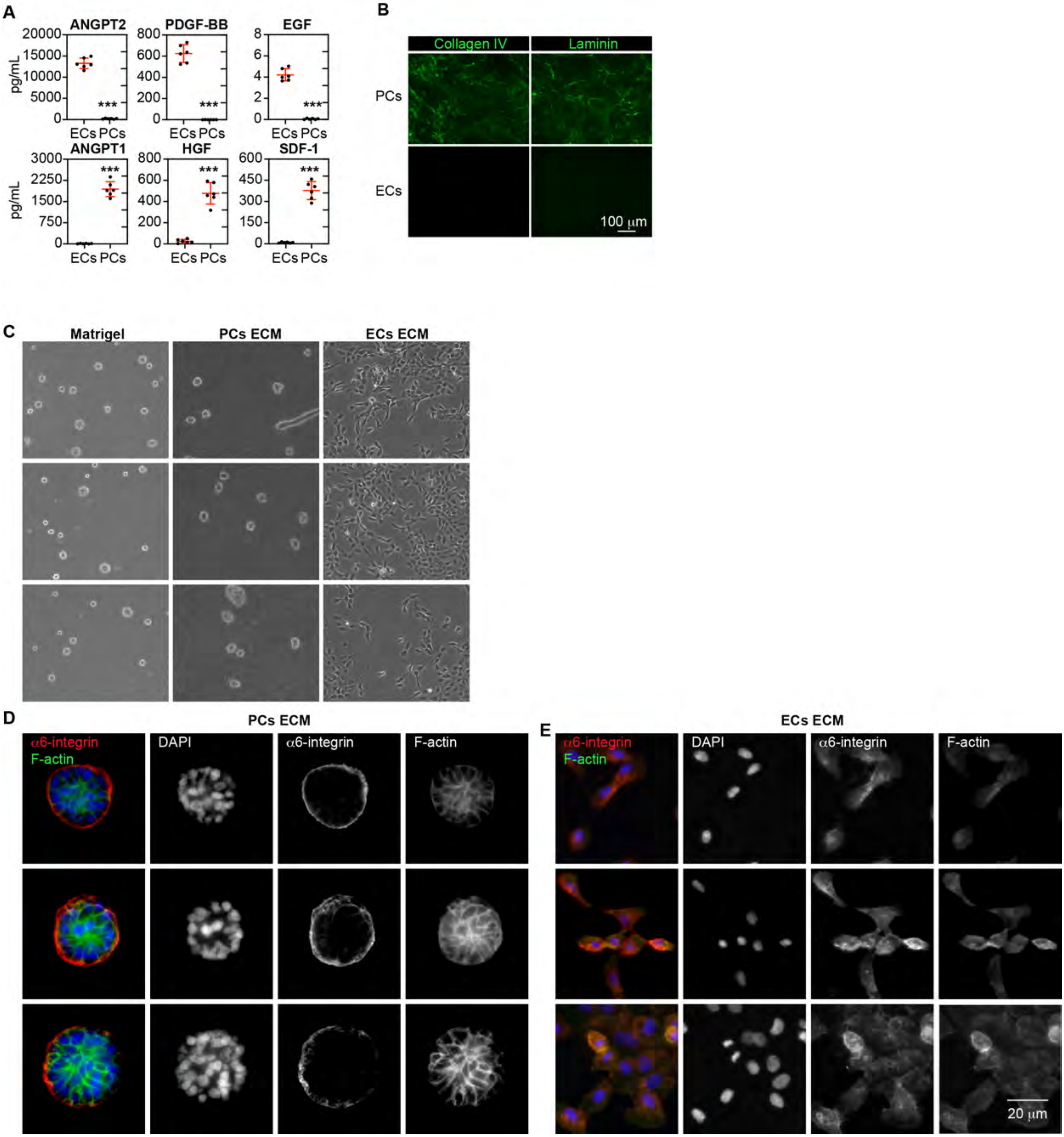

A
ANGPT2
PDGF-BB
EGF
pg/mL
20000
15000
10000
5000
0
800
600
400
200
0
8
6
4
2
0
***
ECs PCs
ANGPT1
HGF
SDF-1
3000
2250
1500
750
600
450
300
150
B
Collagen IV
Laminin
PCs
ECs
100 μm
C
Matrigel
PCs ECM
ECs ECM
D
PCs ECM
α6-integrin
F-actin
DAPI
E
ECs ECM
20 μm

Supplemental Figure 16: Differential expression of soluble factors and basement membrane components by ECs and PCs. (A) Conditioned media (CM) was collected from EC and PC cultures after 24 hours. Multiplexed ELISA quantified the concentration of the indicated secreted factors. (B) To extracellular matrix deposition, PCs and ECs (RM244, RM245, and RM279) were cultured for five days, decellularized, and immunostained for the indicated markers. (C) MCF-10A cells were added to the prepared matrices at a concentration of 20,000 cells for nine days. The resulting epithelial structures were imaged using the 10x objective of a phase contrast microscope. (D) Apicobasal polarization of MCF-10A structures cultured on a decellularized PC matrix was established by staining spheroids with an antibody against α6-integrin (red), F-actin (green), and DAPI (blue). Integrin α6β4 heterodimers connect intermediate filaments to basement membrane components by forming hemidesmosomes. Basal localization of integrin α6 indicates proper polarity. (E) Decellularized breast EC matrix failed to produce polarized epithelial structures when stained as described in D. MCF-10A polarization is a well-established model system highly dependent on laminin. Therefore, MCF-10A is an ideal model to test the functional differences in matrix deposited by vascular cell types. In this experiment, the ECM deposited by PCs could induce MCF-10A polarization, but the ECM deposited by ECs was not.

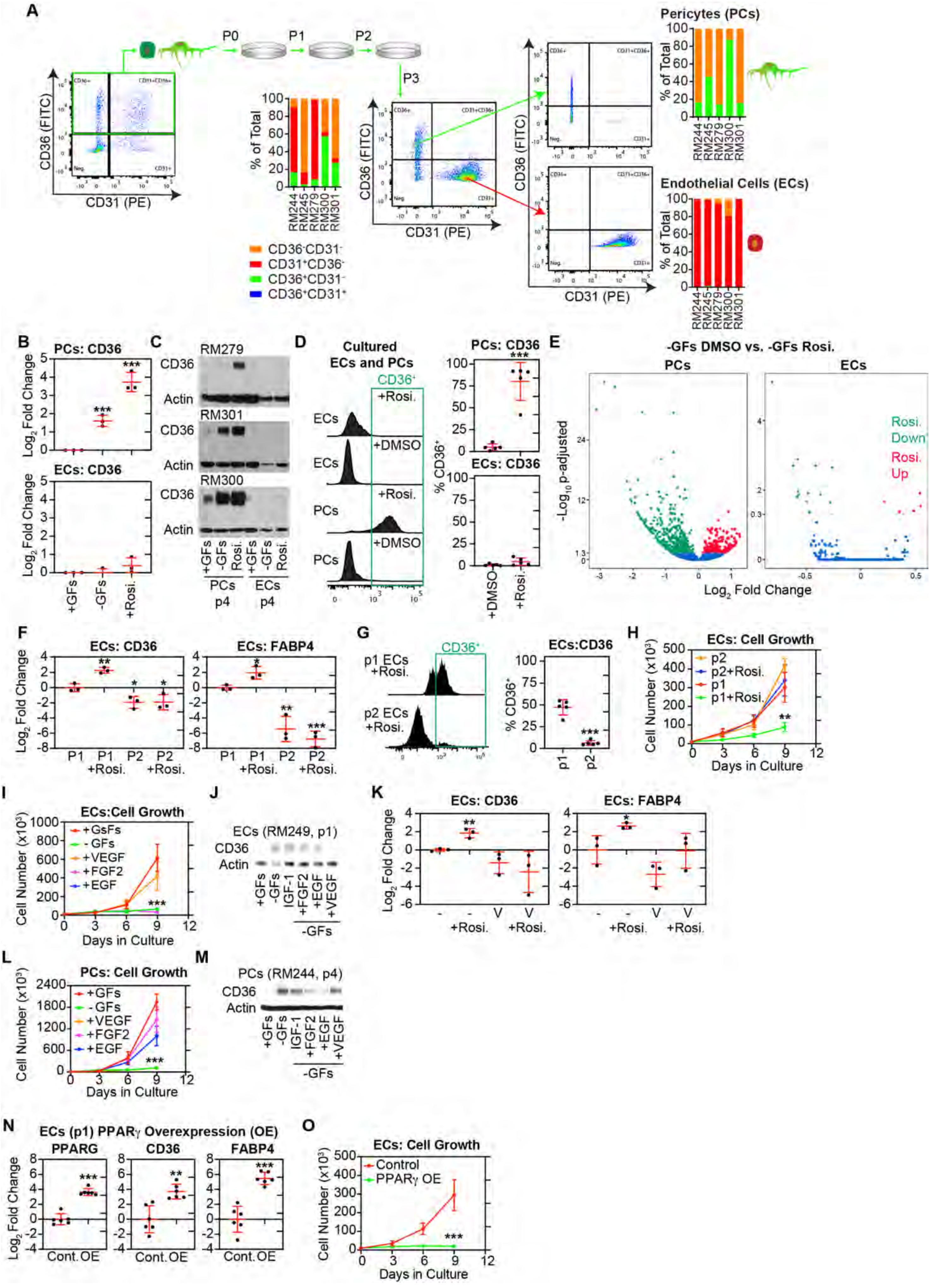

A
P0
P1
P2
P3
CD36 (FITC)
CD31 (PE)
% of Total
RM244
RM245
RM279
RM300
RM301
CD36-CD31-
CD31+CD36-
CD36+CD31-
CD36+CD31+
Pericytes (PCs)
Endothelial Cells (ECs)
B
PCs: CD36
ECs: CD36
Log2 Fold Change
+GFs
-GFs
+Rosi.
C
RM279
RM301
RM300
CD36
Actin
+GFs
-GFs
Rosi.
PCs p4
ECs p4
D
Cultured ECs and PCs
CD36+
+Rosi.
+DMSO
ECs
PCs
PCs: CD36
ECs: CD36
% CD36+
+DMSO
+Rosi.
E
-GFs DMSO vs. -GFs Rosi.
PCs
ECs
-Log10 p-adjusted
Rosi. Down
Rosi. Up
Log2 Fold Change
F
ECs: CD36
ECs: FABP4
P1
P1 +Rosi.
P2
P2 +Rosi.
G
CD36+
p1 ECs +Rosi.
p2 ECs +Rosi.
ECs:CD36
% CD36+
p1
p2
H
ECs: Cell Growth
p2
p2+Rosi.
p1
p1+Rosi.
Cell Number (x10^3)
Days in Culture
I
ECs:Cell Growth
+GsFs
- GFs
+VEGF
+FGF2
+EGF
Cell Number (x10^3)
Days in Culture
J
ECs (RM249, p1)
CD36
Actin
+GFs
-GFs
IGF-1
+FGF2
+EGF
+VEGF
-GFs
K
ECs: CD36
ECs: FABP4
Log2 Fold Change
-
V
+Rosi.
L
PCs: Cell Growth
+GFs
- GFs
+VEGF
+FGF2
+EGF
Cell Number (x10^3)
Days in Culture
M
PCs (RM244, p4)
CD36
Actin
+GFs
-GFs
IGF-1
+FGF2
+EGF
+VEGF
-GFs
N
ECs (p1) PPARγ Overexpression (OE)
PPARG
CD36
FABP4
Log2 Fold Change
Cont.
OE
O
ECs: Cell Growth
Control
PPARγ OE
Cell Number (x10^3)
Days in Culture

Supplemental Figure 17: PPARγ activity is incompatible with endothelial proliferation. (A) CD36-expressing cells were isolated directly from breast tissues by FACS, expanded in EGM2-MV medium, and queried for CD31 and CD36 expression at passage 3. Pure EC and PC populations could be derived by separating $CD31^+$ cells (ECs) from $CD31^-$ cells (PCs). (B) Early passage EC and PC populations (P4) were cultured in complete EGM2-MV growth medium (+GFs), in the absence of supplemental growth factors (-GFs), or with rosiglitazone (10 uM) for 24 hours and were analyzed by (B) RT-qPCR and (C) Western blot. (D) Flow cytometry analysis of ECs and PCs cultured with rosiglitazone or DMSO (vehicle control) was also performed. Compared to cells cultured in +GFs medium, the removal of growth factors (-GFs) and the addition of rosiglitazone increased the expression of CD36 at both mRNA and protein levels in PCs but not in ECs. (E) Volcano plots of the $\log_{10}$ adjusted p-values and $\log_2$ fold change determined by whole transcriptome RNA-sequencing of ECs and PCs cultured with rosiglitazone or DMSO (vehicle control). Bulk RNA-sequencing analysis demonstrated that gene expression in cultured ECs was nearly unaltered by rosiglitazone compared to the robust response in PCs. (F, G) To determine if loss of PPARγ activity in cultured ECs occurred in the earliest passages, ECs isolated based on CD31 and CD36 co-expression were treated with rosiglitazone (10 uM) or DMSO for 24 hours at passages one (P1) or two (P2) and analyzed by (F) RT-qPCR and (G) flow cytometry. Indeed, the ability of ECs to upregulate PPARγ target genes (FABP4 and CD36) in response to the strong agonist, rosiglitazone, was already impaired at P2. (H) Rosiglitazone is known to demonstrate anti-angiogenic activity. Cell number was determined over nine days for P1 or P2 ECs cultured with rosiglitazone (10 uM) or DMSO. In this experiment, the anti-angiogenic effect of rosiglitazone was impaired at P2, correlating with the inability of these cells to activate PPARγ target genes. (I) Based on the sustained disruption of the PPARγ transcriptional network in the intratumoral endothelium and ECs expanded in culture, we hypothesized that pro-angiogenic signaling was responsible for the loss of PPARγ expression in ECs. To determine which growth factors in culture media were required for EC proliferation. Cell number was measured for ECs cultured +GFs, -GFs, or in the presence of only IGF-1, VEGF, FGF-2, or EGF. In this experiment, VEGF was necessary and sufficient for EC proliferation. (J) Analysis of lysates from these cultures examined by Western blot suggested that VEGF was also primarily responsible for CD36 downregulation and, thus, impairment of PPARγ activity in expansion conditions. (K) To provide further evidence of VEGF's role in impairing PPARγ activity during EC culture. P1 ECs were treated with rosiglitazone (10 uM) or DMSO for 24 hours in the presence or absence of VEGF (5 ng ml^{-1}). Gene expression analysis by RT-qPCR of CD36 and FABP4 was performed. Indeed, VEGF impaired PPARγ target gene expression in P1 ECs. This result is significant because high levels of VEGF and other pro-angiogenic factors are commonly observed in the tumor microenvironment. (L, M) (L) Cell number was measured for early passage PCs (P4) cultured +GFs, -GFs, or in the presence of only IGF-1 (20ng ml^{-1}), VEGF (5ng ml^{-1}), FGF-2 (10 ng ml^{-1}), or EGF (5 ng ml^{-1}). The mean and standard deviation are shown in red. Welch's t-test determined significance. (M) Lysates from PCs cultured as in A for 48 hours were subjected to western blot analysis for CD36 and actin. This experiment demonstrated that FGF2 and EGF are the components of EGM2-MV medium required for PC expansion and responsible for the downregulation of CD36 and PPARγ activity in PCs. Isolated PCs were expanded in the same medium as ECs (i.e., EGM2-MV). Supplementation with EGF or FGF2 supported the proliferation of PCs in this medium. Notably, these two growth factors specifically repressed CD36 expression, a surrogate marker of PPARγ activity. However, in contrast with ECs, which exhibited irreversible suppression of CD36 at passage 2, CD36 expression could be mostly restored in PCs. (N, O) Due to the inability of ECs to maintain PPARγ activity in the presence of angiogenic factors, we investigated the effect of PPARγ overexpression in early passage ECs compared to control cells transduced with GFP. P1 ECs were transduced with a lentivirus encoding human PPARγ or GFP (control) and analyzed by RT-qPCR. (N) PPARγ overexpression dramatically induced the transcription of target genes, CD36 and FABP4, without rosiglitazone. (O) Following selection for one week, cell number was determined for GFP-expressing control and PPARγ-overexpressing (OE) ECs. In several attempts, we could not establish ECs overexpressing PPARγ in culture; however, we could establish stable cultures of GFP-expressing ECs. These results suggest that repression of the PPARγ pathway was necessary to expand ECs in culture. For each graph, the mean and standard deviation are shown in red. Welch's t-test determined significance.

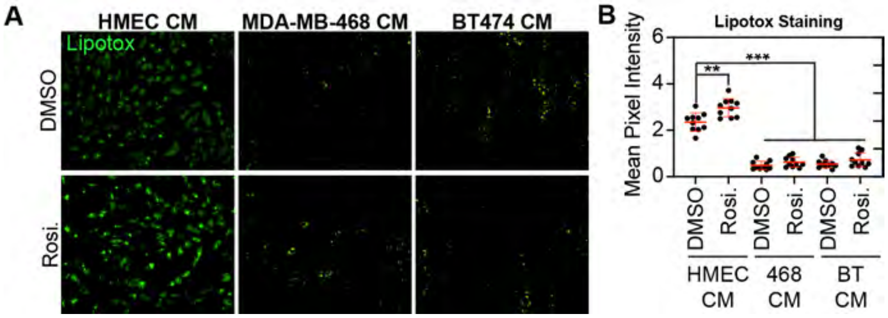
A
HMEC CM
MDA-MB-468 CM
BT474 CM
Lipotox
DMSO
Rosi.
B
Lipotox Staining
Mean Pixel Intensity
**
***
DMSO
Rosi.
DMSO
Rosi.
DMSO
Rosi.
HMEC CM
468 CM
BT CM

Supplemental Figure 18: Conditioned media from tumor cell lines impairs adipocyte lipid uptake. (A) These adipocyte cultures were stained with lipotox to visualize the intracellular accumulation of neutral lipids. (B) The relative abundance of neutral lipid accumulation was quantified by measuring average pixel intensity in ten 20x fields.

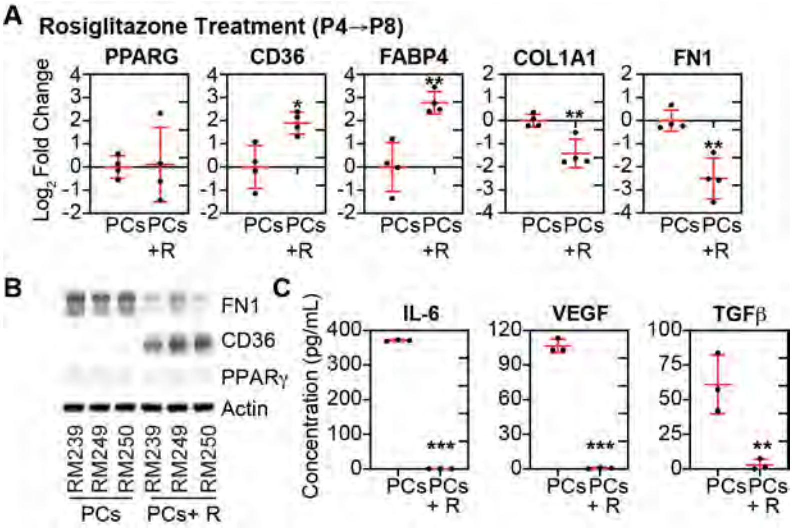
A
Rosiglitazone Treatment (P4→P8)
PPARG
CD36
FABP4
COL1A1
FN1
Log2 Fold Change
PCs
PCs +R
B
FN1
CD36
PPARγ
Actin
RM239
RM249
RM250
PCs
PCs+ R
C
IL-6
VEGF
TGFβ
Concentration (pg/mL)
PCs
PCs + R

Supplemental Figure 19: Rosiglitazone suppresses the acquisition of myofibroblasts properties by PCs (A) PCs were treated with rosiglitazone (10 uM) or DMSO from P4 to P8. PPAR, CD36, FABP4, COL1A1, and FN1 gene expression analysis by RT-qPCR was performed. (B) Lysates from PCs cultured as described above were subjected to western blot analysis for FN1, CD36, PPARγ , and actin. (C) CM was collected after 24 hours from PCs cultured as above and subjected to sandwich ELISA analysis for IL-6, VEGFA, and TGFβ. In this experiment, rosiglitazone treatment of PCs maintained PPARγ target gene activity, reduced the expression of collagen and FN1, and reduced the elaboration of IL-6, VEGF, and TGF-β.

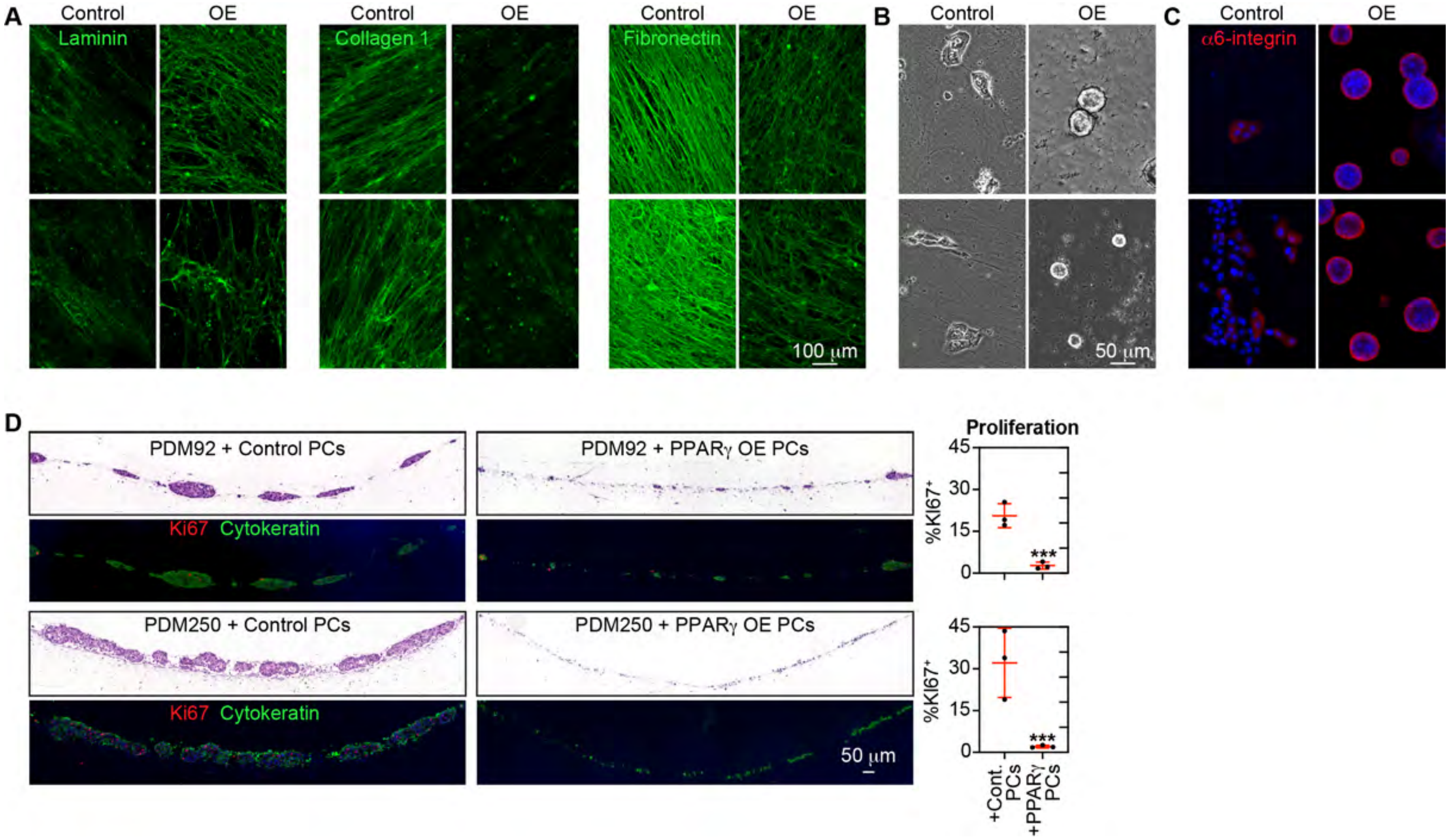
A
Control
OE
Laminin
Collagen 1
Fibronectin
100 μm
B
Control
OE
50 μm
C
Control
OE
α6-integrin
D
PDM92 + Control PCs
PDM92 + PPARγ OE PCs
Ki67 Cytokeratin
PDM250 + Control PCs
PDM250 + PPARγ OE PCs
Ki67 Cytokeratin
50 μm
Proliferation
%KI67+
45
30
15
0
***
+Cont. PCs
+PPARγ PCs

Supplemental Figure 20: PPARγ activation suppressed pro-tumorigenic phenotypes associated with PC-derived myofibroblasts. (A-D) PCs were transduced with a human PPARγ or GFP (control) lentiviral construct at P4 and expanded/selected for four additional passages. At P8, PCs expanded in the presence of growth factors, and the absence of endothelial cells typically transitioned toward a myofibroblast phenotype. (A) Decellularized matrices from GFP and PPARγ-OE PCs were immunostained for Laminin, Collagen 1, and Fibronectin. An increase in highly aligned fibronectin and collagen was notable in the matrix laid down by control PCs compared to PCs overexpressing PPARγ. (B-C) MCF10A cells were cultured on a decellularized matrix, visualized by phase contrast microscopy (B), and immunostained with an antibody against α6-integrin (C). When expanded in culture, PCs overexpressing PPARγ elaborated an ECM more conducive to establishing properly polarized MCF10A acini when compared to control PCs. (D) For two weeks, PDM92 or PDM250 breast cancer organoids (20,000 cells) were cultured in transwell inserts containing previously polymerized collagen with either GFP control PCs or PPAR-OE PCs (60,000 cells). The inserts were fixed and embedded in paraffin, then sections, visualized by H&E staining and subjected to mIHC with antibodies against Ki67 (red) and pan-cytokeratin (green). The percentage of cytokeratin^{+} cells that also expressed Ki67 was quantified. In this experiment, PPARγ OE PCs were less supportive of tumor cell expansion and proliferation when compared to GFP control PCs.

MCF10ADCIS.com with GFP⁺ Pericytes

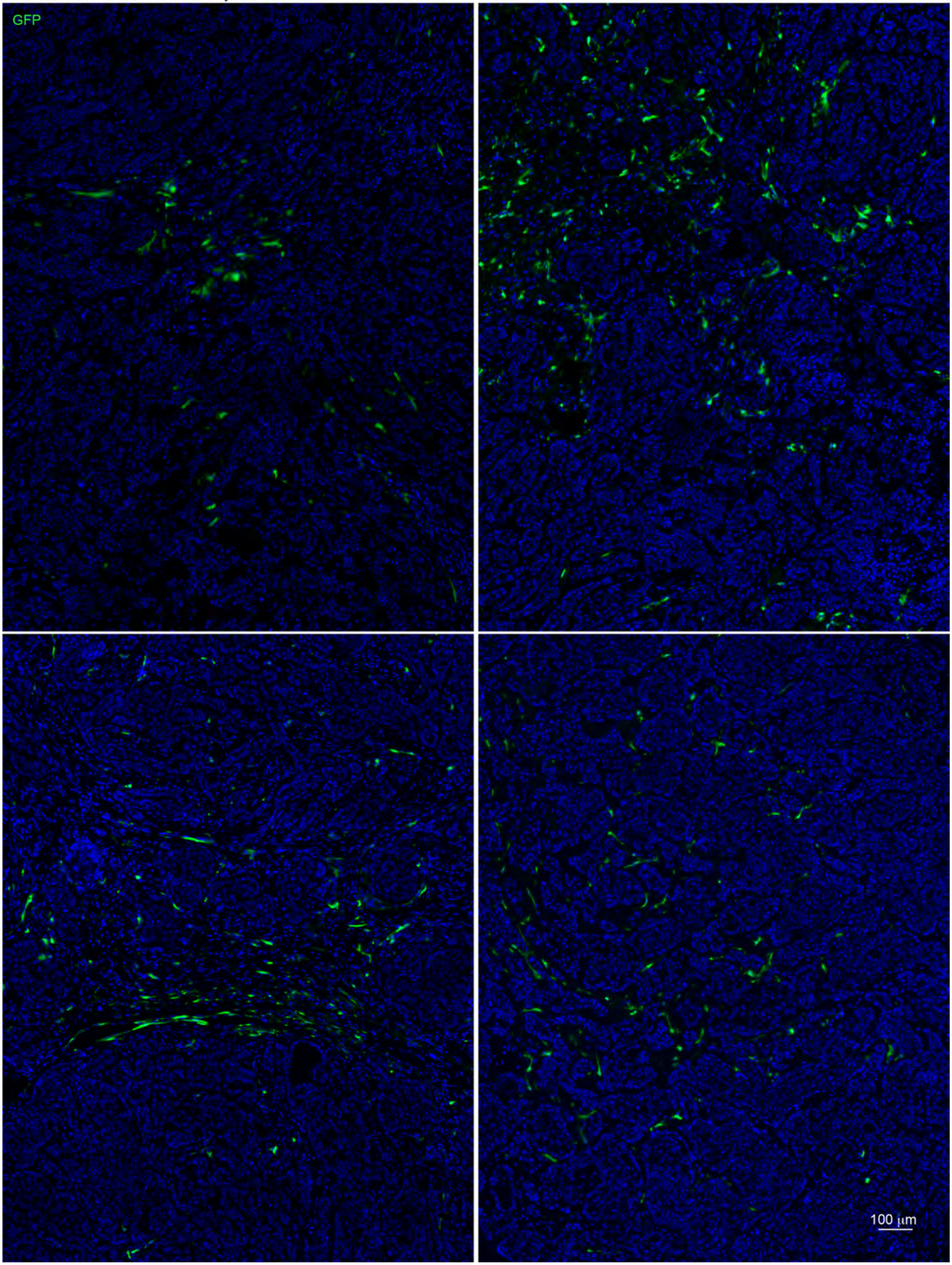

Supplemental Figure 21: PC-derived myofibroblasts integrated into the stroma of MCF10ADCIS.com tumors. PC-derived myofibroblasts (P8; 750,000 cells) were co-injected subcutaneously with MCF10ADCIS.com cells (250,000 cells). The resulting tumors were excised after three weeks. Tumor sections were stained using an antibody against GFP to confirm the presence of GFP-expressing (green) PC-derived myofibroblasts in MCF10ADCIS.com tumor stroma. Images from four tumors are shown. This result confirms that PC-derived myofibroblasts can survive in the MCF10ADCIS.com tumor microenvironment for the duration of the *in vivo* experiment.

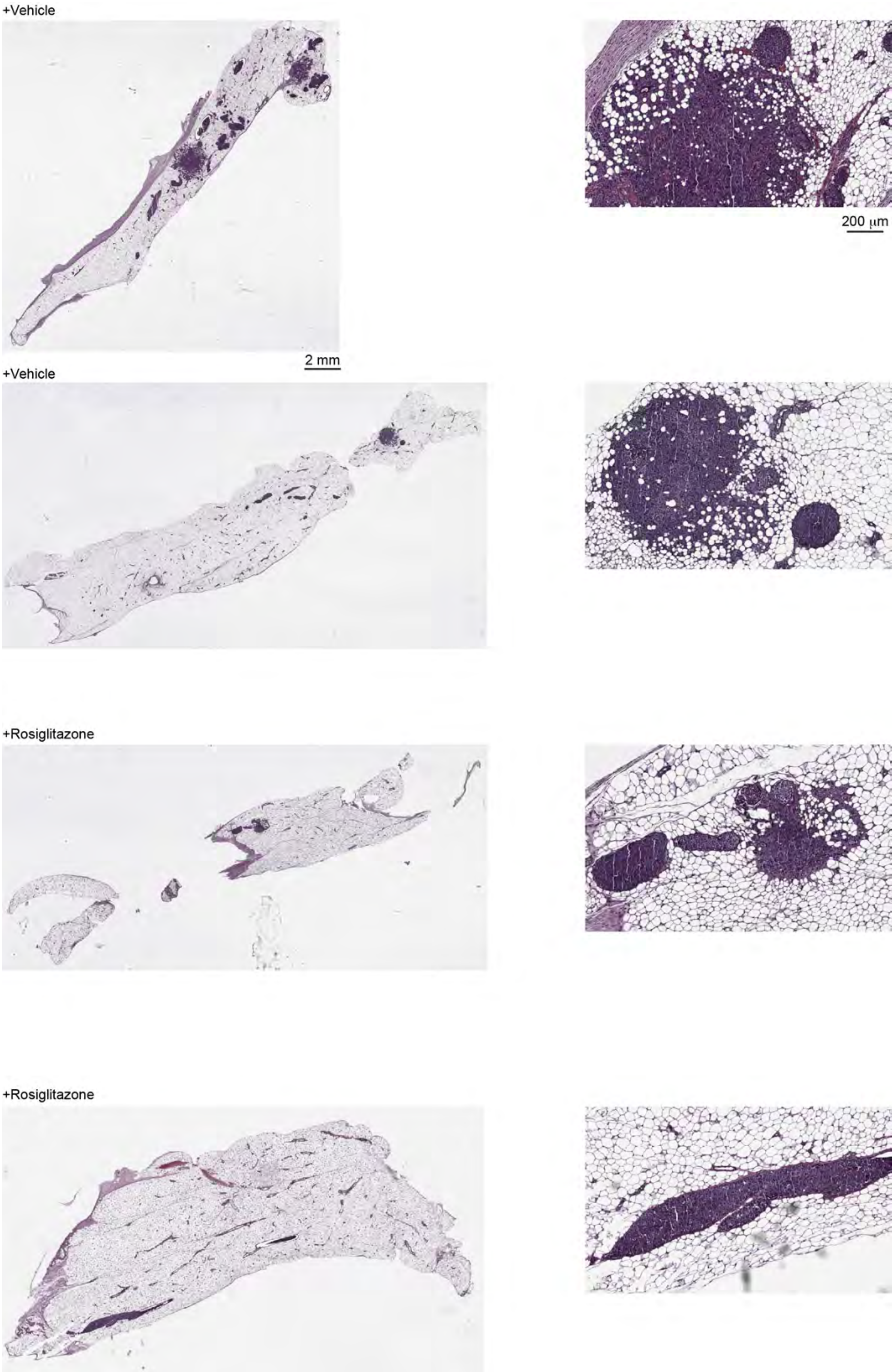

+Vehicle
2 mm
200 μm
+Vehicle
+Rosiglitazone
+Rosiglitazone

Supplemental Figure 22. The anti-tumor efficacy of rosiglitazone in the intraductal model of MDA-MB-468. Triple-negative MDA-MB-468 breast cancer cells were xenotransplanted into NSG mice intraductally through cleaved nipples. The mice were randomized and treated with rosiglitazone (10 mg/kg/day) or vehicle for 31 days. After 45 days, the mammary glands were excised, and lesions were evaluated for invasion into the fat pad. Each mammary gland was sectioned three times at approximately 200 μm intervals to search for invasive growth. This figure shows several examples of the lesions formed in the mouse mammary gland stained by H&E.

+Vehicle

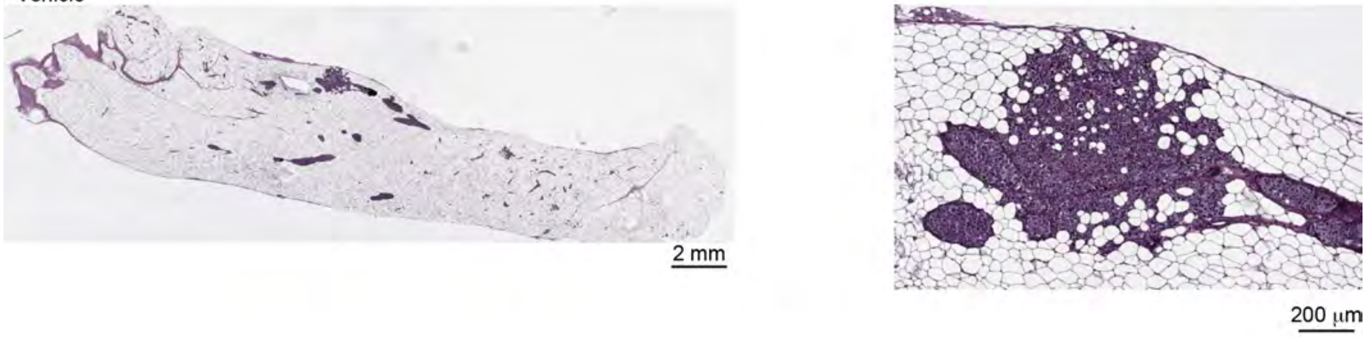

+Vehicle

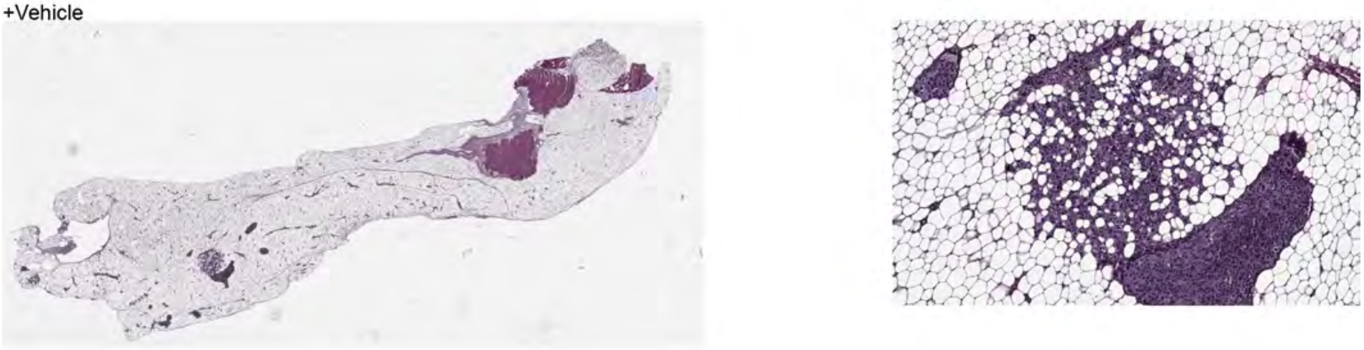

+Rosiglitazone

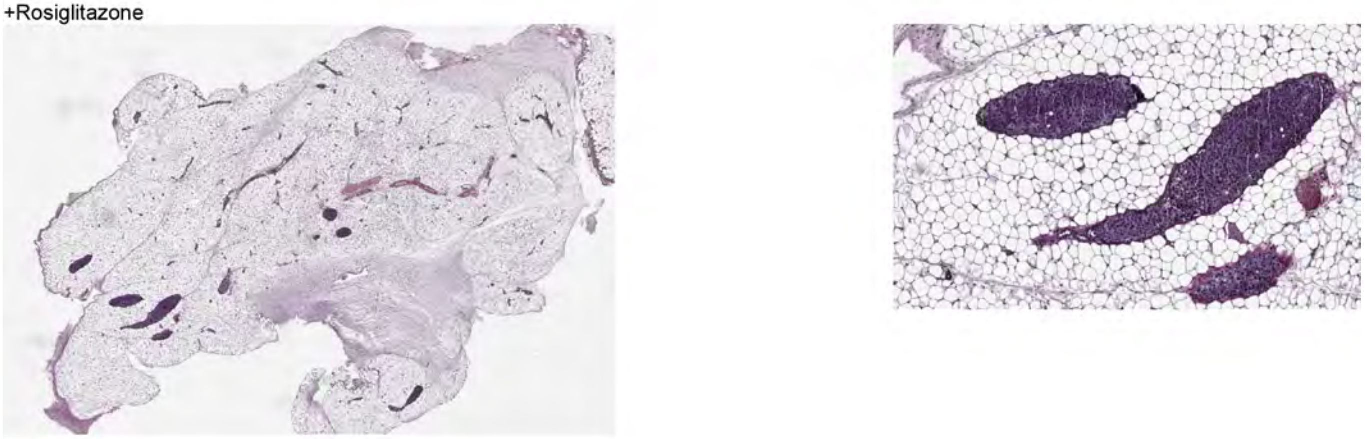

+Rosiglitazone

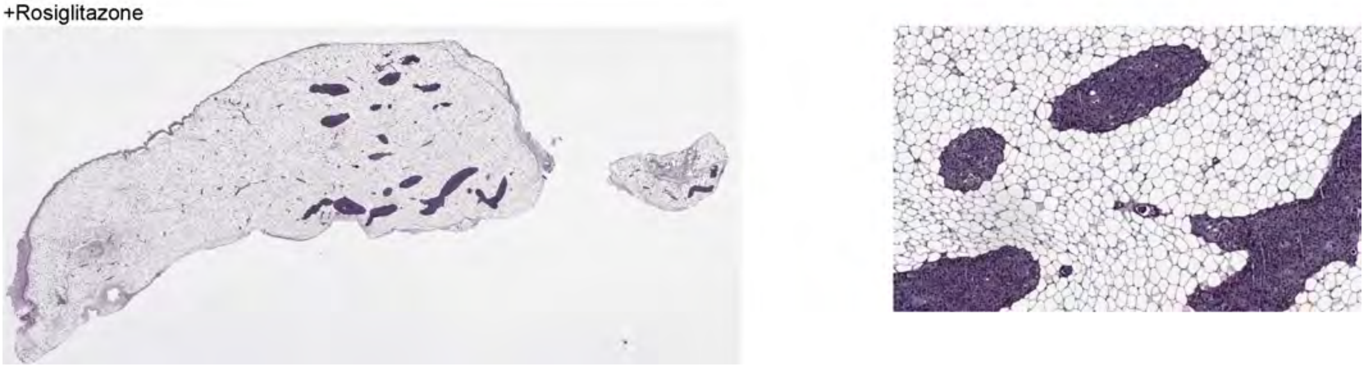

Supplemental Figure 23. The anti-tumor efficacy of rosiglitazone in the intraductal model of BT474. $HER2^+$ BT474 breast cancer cells were xenotransplanted into NSG mice intraductally through cleaved nipples. The mice were randomized and treated with rosiglitazone (10 mg/kg/day) or vehicle for 31 days. After 45 days, the mammary glands were excised, and lesions were evaluated for invasion into the fat pad. Each mammary gland was sectioned three times at approximately 200 μm intervals to search for invasive growth. This figure shows several examples of the lesions formed in the mouse mammary gland stained by H&E.

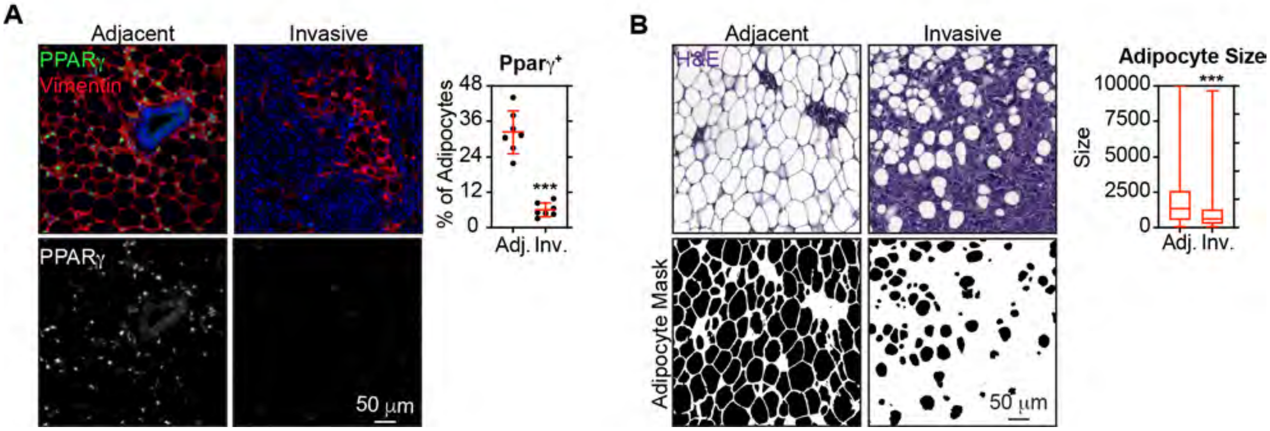
A
Adjacent
Invasive
PPARγ
Vimentin
PPARγ
50 µm
Pparγ+
% of Adipocytes
48
36
24
12
0
***
Adj.
Inv.
B
Adjacent
Invasive
H&E
Adipocyte Mask
50 µm
Adipocyte Size
***
10000
7500
5000
2500
0
Size
Adj.
Inv.

Supplemental Figure S24: Impaired Pparγ expression and lipid uptake in adipocytes surrounded by invasive MDA-MB-468 cells (A) In the intraductal model, Pparγ expression was measured in the stroma (vimentin^{+}, mostly adipocytes) surrounded by invasive tumor cells and compared to adipocytes in unaffected regions of the mouse mammary gland. Shown here for vehicle-treated MDA-MB-468 tumors. (B) The same population of adipocytes as in F was measured in H&E-stained sections.

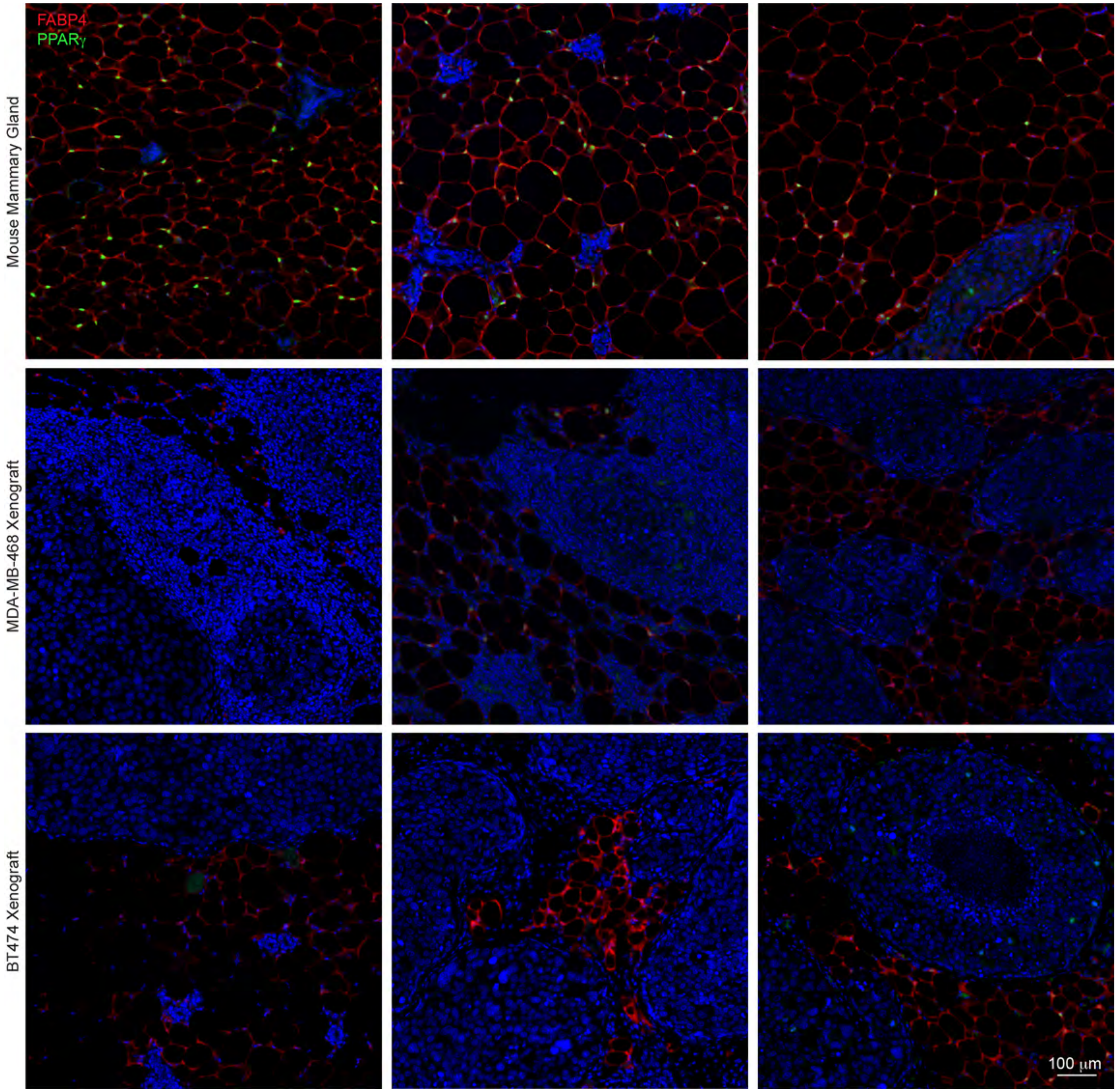

FABP4
PPARγ
Mouse Mammary Gland
MDA-MB-468 Xenograft
BT474 Xenograft
100 µm

Supplemental Figure 25: PPARγ activity is not evident in the MDA-MB-468 nor BT474 models. Xenograft tumor tissues were subjected to mIHC analysis using antibodies against PPARγ and FABP4 and imaged with a 20x objective. No expression was noted in the tumor epithelium.

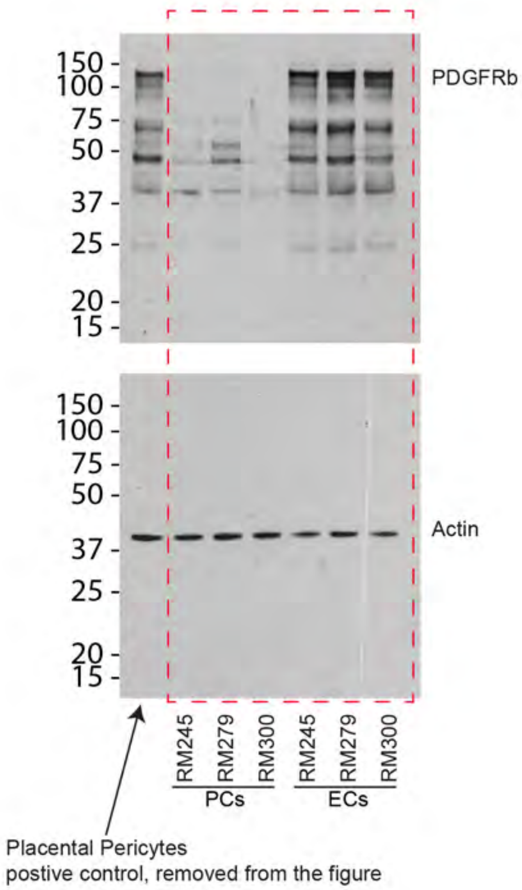
150 -
100 -
75 -
50 -
37 -
25 -
20 -
15 -
PDGFRb
Actin
RM245 RM279 RM300 RM245 RM279 RM300
PCs
ECs
Placental Pericytes
postive control, removed from the figure

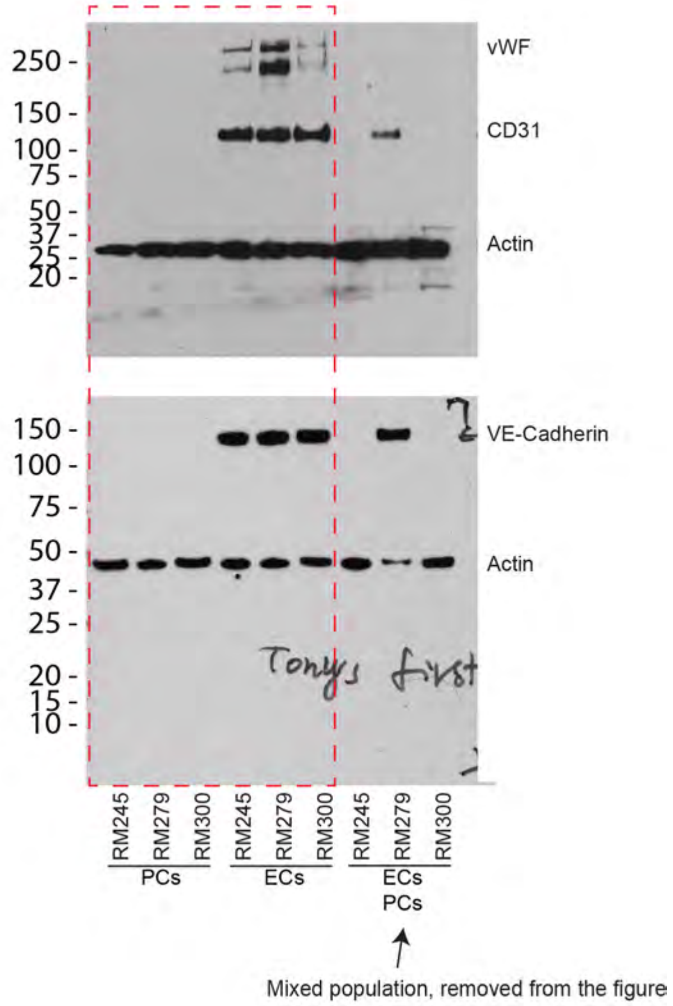
250 -
150 -
100 -
75 -
50 -
37 -
25 -
20 -
15 -
10 -
vWF
CD31
Actin
VE-Cadherin
Actin
Tonys first
RM245 RM279 RM300 RM245 RM279 RM300 RM245 RM279 RM300
PCs
ECs
ECs
PCs
Mixed population, removed from the figure

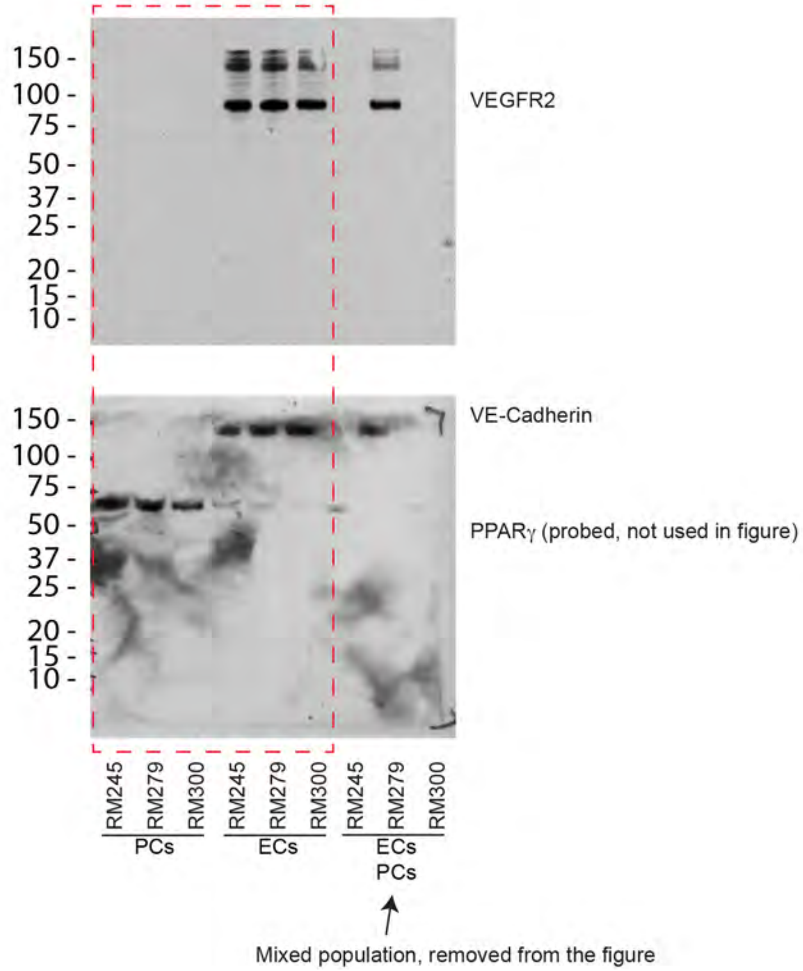
150 -
100 -
75 -
50 -
37 -
25 -
20 -
15 -
10 -
VEGFR2
VE-Cadherin
PPARγ (probed, not used in figure)
RM245 RM279 RM300 RM245 RM279 RM300 RM245 RM279 RM300
PCs
ECs
ECs
PCs
Mixed population, removed from the figure

Supplemental Figure S26: Uncropped western blot images.

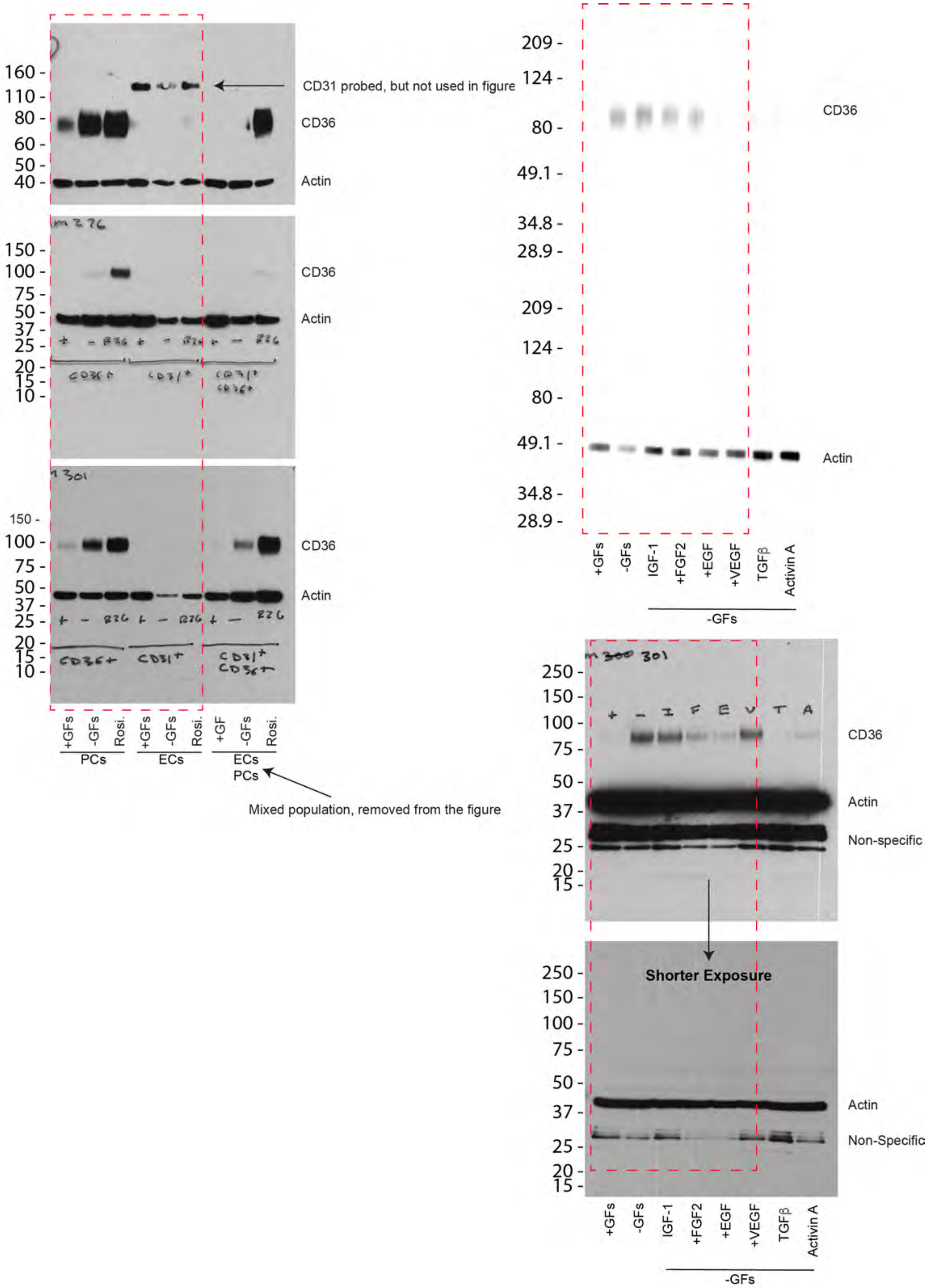
160 -
110 -
80 -
60 -
50 -
40 -
CD31 probed, but not used in figure
CD36
Actin
150 -
100 -
75 -
50 -
37 -
25 -
20 -
15 -
10 -
CD36
Actin
CD36
Actin
+GFs
-GFs
Rosi.
+GFs
-GFs
Rosi.
+GF
-GFs
Rosi.
PCs
ECs
ECs
PCs
Mixed population, removed from the figure
209 -
124 -
80 -
49.1 -
34.8 -
28.9 -
CD36
Actin
+GFs
-GFs
IGF-1
+FGF2
+EGF
+VEGF
TGFβ
Activin A
-GFs
250 -
150 -
100 -
75 -
50 -
37 -
25 -
20 -
15 -
CD36
Actin
Non-specific
Shorter Exposure
Actin
Non-Specific
+GFs
-GFs
IGF-1
+FGF2
+EGF
+VEGF
TGFβ
Activin A
-GFs

Supplemental Figure S27: Uncropped western blot images.

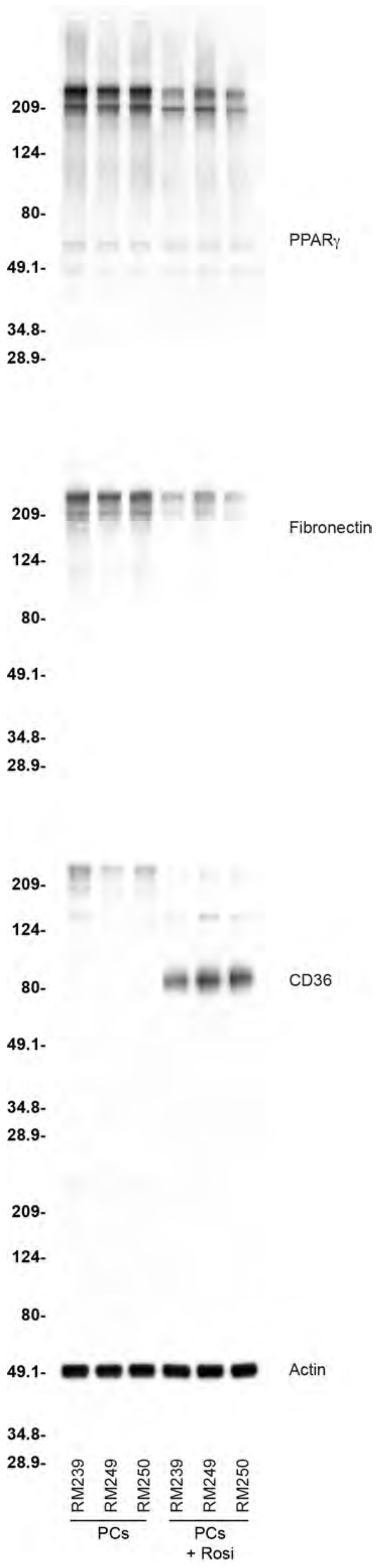
209-
124-
80-
49.1-
34.8-
28.9-
PPARγ
209-
124-
80-
49.1-
34.8-
28.9-
Fibronectin
209-
124-
80-
49.1-
34.8-
28.9-
CD36
209-
124-
80-
49.1-
34.8-
28.9-
Actin
RM239
RM249
RM250
RM239
RM249
RM250
PCs
PCs + Rosi

Supplemental Figure S28: Uncropped western blot images.

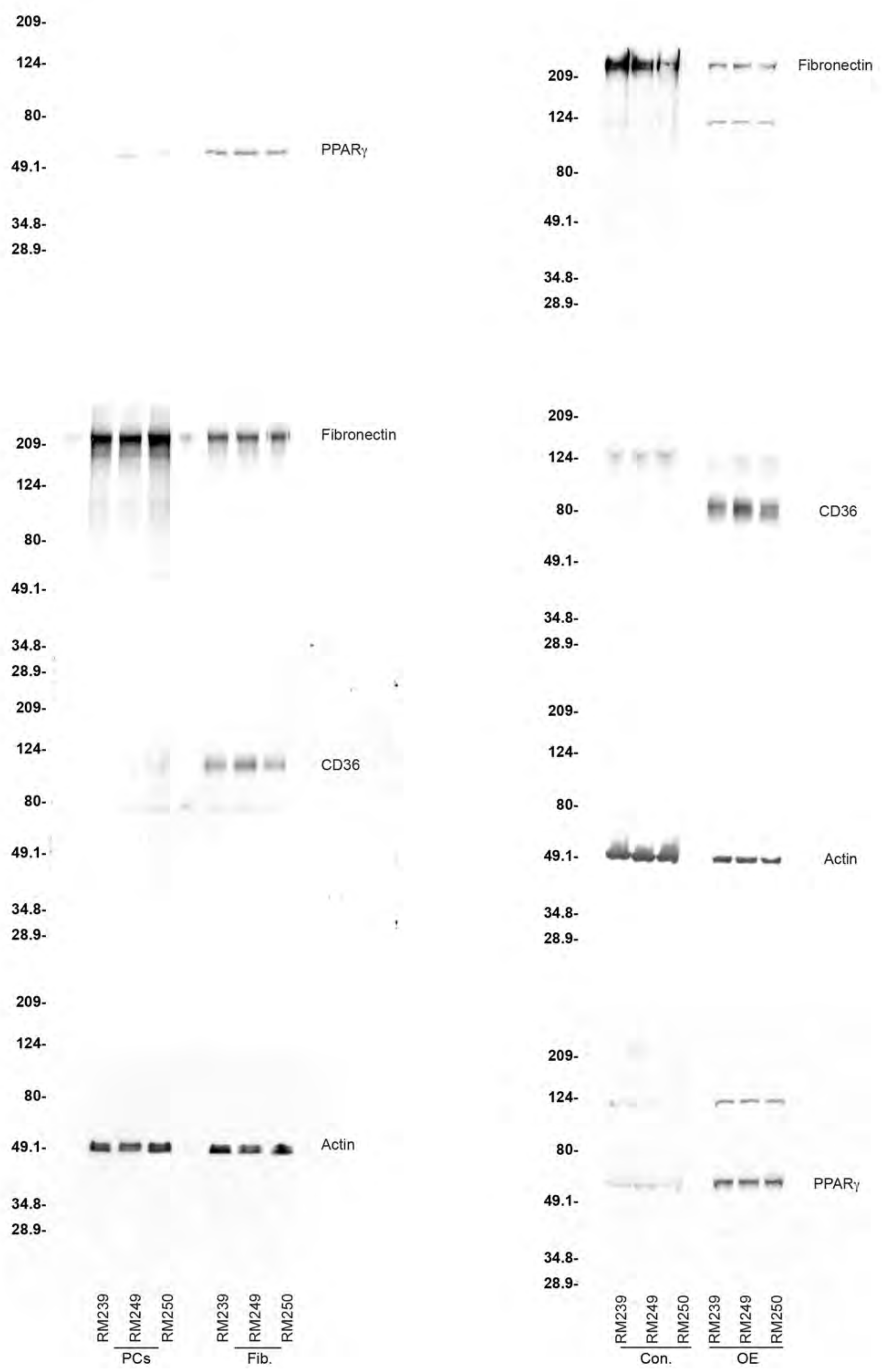
209-
124-
80-
49.1-
34.8-
28.9-
PPARγ
Fibronectin
CD36
Actin
RM239
RM249
RM250
PCs
Fib.
Con.
OE

Supplemental Figure S2: Uncropped western blot images.

Supplemental Datasets:

Dataset S1: Differential gene expression analysis identifies distinguishing features of the vascular EC1 cluster. Differential gene expression analysis was conducted using the Wilcoxon rank-sum test. Columns: (A) gene; (B) p_val = unadjusted p-value; (C) avg_logFC = $\log_2$-transformed fold-change of average gene expression between the vascular EC1 cluster and the other clusters, positive values indicate that the gene is more highly expressed in the vascular EC1 cluster; (D) pct.1 = the proportion of cells in the vascular EC1 cluster where the feature is detected; (E) pct.2 = the proportion of cells in the other clusters where the feature is detected; (F) p_val_adj = adjusted p-value based on Bonferroni correction using all features in the dataset. Rows: genes.

Dataset S2: Differential gene expression analysis identifies distinguishing features of the vascular EC2 cluster. Columns and Rows are the same as in Dataset S1.

Dataset S3: Differential gene expression analysis identifies distinguishing features of lymphatic EC cluster. Columns and Rows are the same as in Dataset S1.

Dataset S4: Differential gene expression analysis identifies distinguishing features of the pericyte cluster. Columns and Rows are the same as in Dataset S1.

Dataset S5: Differential gene expression analysis of PCs cultured with rosiglitazone or DMSO. Columns: (A) Gene_ID = Ensembl gene annotation; (B) PC_DMSO: normalized readcounts from pericytes (PCs) cultured with DMSO; (C) PC_Rosi: normalized readcounts from the PCs cultured with rosiglitazone; (D) $\log_2$FoldChange= $\log_2$-transformed fold-change between groups, positive values indicate that the gene is more highly expressed in the DMSO group; (E) Pvalue = unadjusted p-value; (F) padj = Benjamini and Hochberg adjusted p-value; (G) GeneName = official gene symbol; (H) Chrom = chromosome where the gene is located; (I) Strand = DNA strand, sense (+) or antisense (-); (J) Start = the start position of the gene; (K) End = the end position of the gene; (L) GeneLength = length of the gene; (M) GeneType = type of gene (e.g. protein coding, lincRNA, etc.); (N) GeneDescription = description of gene; (O-Z) readcount = readcount for all samples; (AA-AL) fpkm = Fragments Per Kilobase of transcript per Million mapped reads for all samples. Rows: genes.

Dataset S6: Differential gene expression analysis of ECs cultured with rosiglitazone or DMSO. Columns and Rows are the same as in Dataset S5.

Dataset S7: Proteins detected in conditioned media from ECs and PCs. Columns: (A) LOD = Limit of Detection (pg/mL); (B) MAX = maximum value (pg/mL); (C) Target: protein analyzed; (D-O) Concentration of each target in endothelial cells (ECs) and pericytes (PCs), light blue values were below the assay minimum; (P) Discovery? = Two-stage step-up (Benjamini, Krieger, and Yekutieli) FDR method (Q=1%) was used to decide if each P value is small enough to be designated a discovery: yes or no; (Q) p-value: Welch's t-test; (R) Mean of ECs (pg/mL); (S) Mean of PCs (pg/mL); (T) Difference= between EC and PC; (U) SE of difference = Standard Error; (V) t ratio = ratio of the difference between the mean of EC and PC and the variation that exists within the sample sets; (W) df = degrees of freedom; (X) q value = FDR-adjusted p values. Rows: targets.

Dataset S8: Proteins detected in conditioned media from pericytes (PCs) and pericyte-derived fibroblasts (PC→Fibs). Columns and Rows are the same as in Dataset S7.

Dataset S9. Antibodies utilized. Columns: (A) Target; (B) Species; (C) Manufacturer; (D) Clone: for monoclonal antibodies; (E) Catalog Number; (F) Primary Dilution for mIHC; (G) Primary Dilution for IHC (H) Primary Dilution for confocal microscopy; (I) ul per 1e6 cells: μls of antibody needed to stain 1e6 cells in 100 μL of PBS with 1% BSA for analysis by flow cytometry; (J) Fluorophore or biotin directly conjugated to the antibody.
